# Neddylation of insulin receptor substrate acts as a bona fide regulator of insulin signaling and its implications for cancer cell migration

**DOI:** 10.1038/s41417-024-00729-z

**Published:** 2024-01-25

**Authors:** Jun Bum Park, Geon Ho Moon, Ara Cho, Minji Kwon, Jong-Wan Park, Eugene C. Yi, Haeryoung Kim, Junji Fukuda, Cheol Kwak, Young-Gyu Ko, Yang-Sook Chun

**Affiliations:** 1 Department of Biomedical Sciences, Seoul National University College of Medicine, Seoul, 03080 Republic of Korea; 2 Ischemic/Hypoxic Disease Institute, Seoul National University College of Medicine, Seoul, 03080 Republic of Korea; 3 Department of Molecular Medicine and Biopharmaceutical Sciences, Graduate School of Convergence Science and Technology, and Seoul National University College of Medicine, Seoul, 03080 Republic of Korea; 4 Department of Pathology, Seoul National University Hospital, Seoul National University College of Medicine, Seoul, 03080 Republic of Korea; 5 Faculty of Engineering, Yokohama National University, Yokohama, 240-8501 Japan; 6 Department of Urology, Seoul National University Hospital, Seoul, 03080 Republic of Korea; 7 Division of Life Sciences, Korea University, Seoul, 02841 Republic of Korea; 8 Department of Physiology, Seoul National University College of Medicine, Seoul, 03080 Republic of Korea

**Keywords:** Cancer genetics, Cell biology, RNAi, Gene expression, Metastasis

## Abstract

Irregularities in insulin signaling have significantly increased the risk of various cancers, yet the precise underlying mechanisms remain unclear. Within our study, we observed that inhibiting neddylation enhances cancer cell migration across different cancer types by activating both insulin receptor substrates 1 and 2 (IRS1 and IRS2), along with the PI3K/AKT signaling pathway. Notably, in the context of high-grade serous carcinoma (HGSC) patients, whether they had type 2 diabetes mellitus or not, IRS1 and IRS2 displayed a parallel relationship with each other while exhibiting an inverse relationship with NEDD8. We also identified C-CBL as an E3 ligase responsible for neddylating IRS1 and IRS2, with clinical evidence further confirming a reciprocal relationship between C-CBL and pAKT, thereby reinforcing the tumor suppressive role of C-CBL. Altogether, these findings suggest that neddylation genuinely participates in IRS1 and IRS2-dependent insulin signaling, effectively suppressing cancer cell migration. Thus, caution is advised when considering neddylation inhibitors as a treatment option for cancer patients, particularly those presenting with insulin signaling dysregulations linked to conditions like obesity-related type 2 diabetes or hyperinsulinemia.

## Introduction

The association between cancer and diabetes has garnered significant attention in recent years due to their overlapping pathophysiological mechanisms and epidemiological connections. Epidemiological studies have consistently demonstrated an increased risk in a wide range of cancer types, including frequently reported cases such as pancreatic, colorectal, prostate, and ovarian cancers in type 2 diabetes mellitus (T2DM) patients as well as less studied cancers like renal, bladder, and brain cancer [1–7]. Insulin dysregulation is a key feature of type 2 diabetes that interferes with the insulin signaling pathways and has been identified as a risk factor for the development and progression of various types of cancer [8].

Insulin receptor substrates 1 and 2 (IRS1 and IRS2) play pivotal roles in insulin signaling, acting as intermediaries that transmit insulin’s effects to cellular processes closely associated with cancer. Activation of IRS proteins occurs in cancer cells when insulin binds to the Insulin Receptor (IR), leading to a chain of intricate signaling cascades. IRS proteins are found at higher levels in human cancers compared to normal tissues, implying their potential involvement in tumorigenesis. Most cancer types also exhibit elevated expression levels of IRS proteins, hinting at the potential diverse and context-dependent roles of IRS proteins in cancer [9].

Understanding the diverse mechanisms that activate IRS1 and IRS2 is crucial, calling for extensive research at the protein levels and beyond. Posttranslational modifications are currently being closely examined as a key factor in regulating the activities of IRS proteins and their interactions with downstream effectors. Notably, important modifications such as phosphorylation, ubiquitination, O-GlcNAcylation, and acetylation play critical roles in various cellular processes and disease states [10–14]. Studies have linked high blood sugar to abnormal fat buildup inside cells, which is influenced by protein kinase C affecting IRS serine/threonine phosphorylation. Additionally, acetylation of IRS1 and IRS2 has been observed to influence insulin signaling by reducing their association with IRβl [14, 15]. To gain a deeper understanding of their regulatory functions, further research into the posttranslational modifications of IRS proteins is essential.

Neddylation is a posttranslational modification process that entails the attachment of the small ubiquitin-like molecule NEDD8 to target proteins. This process has become an important regulatory mechanism that controls many cellular processes such as protein degradation, DNA repair, cell migration, and cell cycle progression [16]. Based on our previous research in cancer, we have discovered that neddylation plays a crucial role in regulating cancer cell migration. This is particularly evident in its impact on the PI3K/AKT pathway in multiple types of cancer [17–19]. Despite numerous studies on neddylation and its effects on various cellular processes, a thorough comprehension of its impact on IRS proteins and their role in cancer cell migration is yet to be achieved.

Our study aims to explore the molecular function of IRS1 and IRS2 in relation to neddylation and its effect on cancer cell migration. The inhibition of neddylation led to the increased abundance of both IRS1 and IRS2, resulting in the activation of the PI3K/AKT pathway. Our research underscores the significant role of Cbl proto-oncogene (C-CBL) as a neddylation E3 ligase for IRS1 and IRS2, highlighting the promising potential of C-CBL as a target for regulating cancer metastasis in the context of insulin dysregulation.

## Materials/subjects and methods

### Human tissue

In strict adherence to the Declaration of Helsinki, all human tissue procedures were granted approval by the Institutional Review Board (IRB) of Seoul National University Hospital (SNUH). The study focuses on specimens obtained from high-grade serous carcinoma patients who underwent surgical resection at SNUH’s Pathology Department between 2019 and 2022. Restricted to samples registered in SNUH’s Tissue Bank for Cancer with secondary consent for comprehensive research, the tissue samples are exclusively sourced from human donors who have provided consent through donation agreements. Demographic data (age, gender, BMI, T2DM diagnosis, and FIGO stage) were gathered and anonymized in accordance with the research protocol (Supplementary Table 1). These specimens, collected from SNUH’s Tissue Bank, encompass two groups of high-grade serous carcinoma patients: one with type 2 diabetes mellitus and one without, each group consisting of 15 female patients, aged 36 to 88 years, with an average age of 65.1 ± 11.376 years.

### Cell cultures

Human embryonic kidney (HEK293) cells were obtained from the American Type Culture Collection (Manassas, VA, USA). SKOV3 (human ovarian cancer) and U373 (human glioblastoma) cancer cells were obtained from the Korea Cell Line Bank (Seoul, Republic of Korea). RCC4 (human kidney cancer) cells were kindly provided by Professor Cheol Kwak from Seoul National University Hospital. HEK293 and RCC4 cells were cultured in Dulbecco’s modified Eagle’s medium (DMEM). SKOV3 and U373 cells were cultured in RPMI1640 medium. All media were supplemented with 10% heat-inactivated fetal bovine serum (FBS).

### Volcano plot

To determine differentially expressed genes between low- and high-grade ovarian cancer patients, | log2(fold change) | >1 and *p* value < 0.001 were used as cut-offs. Gene expression profile data GSE73168 was collected from the National Center for Biotechnology Information’s Gene Expression Omnibus (GEO, NCBI). GSE73168 included 15 high-grade serous ovarian cancer samples and 9 low-grade serous ovarian cancer samples. We analyzed gene expression of NAE1(202268_s_at), UBA1(200964_at) and SAE1(241185_at) in gene sets.

### Gene set enrichment analysis

Normalized enrichment score (NES) and nominal *p* value were calculated using the Broad Institute GSEA software. A nominal *p* value < 0.05 was considered significant. Ovarian cancer patients were divided into two groups based on the mRNA level of NAE1(202268_s_at; GSE73168) using k-means clustering with R-package v.4.4.2. *Cellular response to insulin stimulus* gene set and *ovarian cancer LMP DN* gene set were obtained from MsigDB. GSEA was performed using the GSEA software v.4.3.2.

### Survival plot

The survival curves were measured based on the Kaplan-Meier method and compared using the log-rank test in ovarian cancer patients. Gene expression data were obtained from GDC TCGA Ovarian Cancer using the UCSC Xena database (https://xena.ucsc.edu/). A *p* value < 0.05 was considered significant.

### KEGG functional analysis

To identify proteins that regulate cancer malignancy in response to insulin, we screened three independent pathways. IRS1 is a sub-gene commonly belonging to PI3K-AKT signaling pathway (map04151), Insulin signaling pathway (map04910), and Type II diabetes mellitus (map04930). Data were obtained from KEGG database from Kyoto University (http://www.genome.jp/kegg/expression/).

### Sample preparation for LC-MS/MS analysis

Proteins eluted from His_6_-tagged NEDD8 conjugation was reduced with 20 mM dithiothreitol at 95 °C for 10 min, followed by alkylation with 40 mM iodoacetamide at room temperature in the dark for 30 min. Proteins were then acidified by phosphoric acid and loaded onto the S-trap Micro Spin Column (Protifi). After multiple washes with the S-trap binding buffer [90% MeOH, 100 mM of triethylammonium bicarbonate (TEAB)], 1 μg of trypsin in 50 mM TEAB was then added onto the column and incubated at 37 °C overnight. Digested peptides were eluted with each of the following solutions: 50 mM TEAB, 0.1% formic acid (FA) and 50% acetonitrile with 0.1% FA, and combined. The peptides were dried down prior to resuspension in 0.1% FA for liquid chromatography–mass spectrometry (LC-MS) analysis.

### LC-MS/MS analysis

The dried peptides were resuspended in 20 uL of Solvent A (0.1% formic acid in water) and 2 ug of sample was loaded onto an analytic column (PepMapTM, RSLC 75 μm ID*50 cm 2 μm, Thermo Fisher Scientific). The duplicate analysis was conducted and loaded samples were separated with a gradual gradient of 5–35% Solvent B (0.1% formic acid in Acetonitrile) for 90 min at 300 nL/minutes flow; 0–10 min 2% of solvent B, 11–12 min 5% of solvent B, 13–67 min with 5–35% gradual gradient of solvent B, 68–83 min 70% of solvent B, and 84–90 min 2% of solvent B for column re-equilibration. MS spectra were monitored by Q-ExactiveTM (Thermo Fisher Scientific, San Jose, CA), hybrid quadrupole-orbitrap MS coupled with Ultimate-3000 HPLC system (Thermo Fisher Scientific, San Jose, CA). The standard mass spectrometric condition of spray voltage was set to 2.0 kV and the temperature of the heated capillary was set to 250 °C. The full MS scans were investigated in range between 350 and 1400 mass-to-charge ratio (m/z) with 70,000 resolutions followed by MS/MS scans monitored with 17,500 resolutions of high-energy collisional dissociation (HCD) fragmentation by the 27% of normalized collision energy. The automatic gain control (AGC) target was assigned as 1e5, maximum injection time was 120 ms, and isolation window was set to 2 m/z. The whole analysis was conducted in data-dependent acquisition with individual MS scan followed by ten fragmented MS/MS scans in dynamic exclusion time of 20 s.

### Protein identification

Raw files from the LC-MS analysis were imported into Proteome Discoverer 2.3 (Thermo Fisher Scientific) using the Sequest HT search engine against the UniProt *Homo sapiens* database (downloaded on 27 April 2023). Trypsin was set as the protease with two missed cleavages with sequence lengths between 6 and 144 amino acids. Precursor and fragment mass error tolerances were set to 10 ppm and 0.02 Da, respectively. Peptide dynamic modifications allowed during the search were oxidation (M), deamination (N, Q), and acetylation (N-terminus), whereas carbamidomethyl (C) was set as static modifications. The datasets generated in this study are available via ProteomeXchange with identifier PXD044760.

### Immunohistochemical analysis

Human high-grade serous carcinoma tissue slides obtained from the SNUH’s Tissue Bank were incubated at 60 °C to remove paraffin. Dewaxed slides were mircowaved in antigen retrieval solution, followed by treatment with a 3% H_2_O_2_ solution. The slides were incubated overnight at 4 °C with primary antibodies specific to Insulin (sc-8033), IRS1 (Abcam, ab52167), IRS2 (Abcam, ab134101), NEDD8 (Cell Signaling Technology, 2745S), C-CBL (Santa Cruz Biotechnology, sc-1651), pAKT (Cell Signaling Technology, XBP-4060) and then treated with biotinylated secondary antibodies for 1 h at room temperature. The immune complexes were visualized using a Vectastatin ABC kit from Vector Laboratories (Burlingame, CA, USA), and tissue slides were counterstained with hematoxylin at room temperature.

### Plasmids, short interfering RNAs, and transfection

His-tagged NEDD8, His-mutant NEDD8 (His-NEDD8ΔGG, which lacks ability of conjugating with the target protein), HA-ubiquitin, and Myc-SENP8 were conjugated as described [20]. Flag-IRS1 was kindly donated by Professor Young-Gyu Ko. pBABE puro mouse IRS-2 myc was a gift from Ronald Kahn (Addgene plasmid #11373). Transfection of plasmids or siRNAs was performed using Lipofectamine 2000 or Lipofectamine RNAiMAX reagents. Control, IRS1, IRS2, C-CBL, and NEDD8 siRNAs were synthesized by M. Biotech (Hanam-si, Gyeonggi-do, Republic of Korea). The siRNAs were characterized by the following nucleotide sequences:

Human Control: 5’-UUGAGCAAUUCACGUUCAUTT-3’

Human N8: 5’-AGCGGUAGGAGCAGCAAUUUAUCCG-3’

Human IRS1 (#1): 5’-GAGAAAUCAGUACUGAUGUUACATT -3’

Human IRS1 (#2): 5’-ACAAAGAACCUGAUUGGUAUCUACC -3’

Human IRS2: 5’-AUGUCAGAGAGUAUCAUUAAAAGAA -3’

Human CBL (#1): 5’-GAAGUUACCUAAUAAUCCAAAGATG -3’

Human CBL (#2): 5’-GUUUCCUGAUGGACGAAAUCAGAAT -3’

### Transwell migration assay

Cells (2.5–5 × 10^4^) were suspended in 200 μl serum-free medium containing the specified concentration of reagents and seeded into the upper chamber insert coated with 0.5 mg/mL collagen. The bottom chambers were filled with 500 μl of full-medium containing 10% FBS. After 24 h of incubation, the non-invading cells on the upper membrane surface were gently removed using a cotton swab. The migrated cells were fixed with MeOH and stained using a solution of 0.1% crystal violet in 2% methanol. Images were acquired using an inverted microscope, and the number of cells that migrated to four independent areas per filter was quantified using ImageJ software.

### 3D spheroid cell culture

The 3D spheroid cell culture technique originates from the work of Professor Fukuda [21, 22]. SKOV3 (5 × 10^5^), U373 (1 × 10^6^), and RCC4 (5 × 10^5^) cells were seeded on 4% Pluronic coated PDMS plates and incubated in the oxygen permeable plates for 5 days. Average diameter was measured by Image J using the equation below.

$${\rm{Roundness}}( \% )=100-({\rm{R}}-{\rm{r}})/{\rm{R}}\,{\rm{x}}\,100$$

(R = the radius of the minimum circumscribed circle and r = the radius of the maximum inscribed concentric circle)

### Identification of His_6_-tagged NEDD8 conjugates

NEDD8 conjugation was identified following the established protocol [23]. Following transfection with either His_6_-tagged NEDD8 or NEDD8-ΔGG mutant plasmids, the cells were divided into two dishes. One was lysed with 2× SDS sample buffer and subjected to immunoblot analysis to assess protein expression levels (input samples). The remaining dish was mixed with denaturing buffer (6 M guanidine hydrochloride, 0.1 M Na_2_HPO_4_/NaH_2_PO_4_, 0.01 M Tris-Cl (pH 8.0), 10 mM imidazole and 10 mM 2-mercaptoethanol). The lysates were then incubated with Ni^2+^-NTA-agarose beads at 4 °C for 4 h. After incubation, the beads were washed for 2 min at each step with the following solutions: lysis buffer (pH 8.0); washing buffer (pH 8.0, composed of 8 M urea, 0.1 M Na_2_HPO_4_/NaH_2_PO_4_, 0.01 M Tris/HCl, 20 mM imidazole and 10 mM 2-mercaptoethanol); washing buffer (pH 6.3) supplemented with 0.2% Triton X-100; and washing buffer (pH 6.3) containing 0.1% Triton X-100. The bound proteins were then eluted in a 2x SDS denaturing buffer and subjected to western blotting.

### Western blotting and immunoprecipitation

Cell lysates were subjected to SDS-polyacrylamide gel electrophoresis and subsequently transferred to Immobilon-P membranes (Millipore, Bedford, MA, USA). Membranes were blocked with a mixture of 1% skim milk and 1% bovine serum albumin (BSA) or 3% BSA in Tris/saline solution containing 0.1% Tween-20 (TTBS) for 1 hour. Subsequently, they were incubated overnight at 4 °C with primary antibodies specific to IRS1 (Cell Signaling Technology, 2382 S), IRS2 (Cell Signaling Technology, 4502S), pAKT (Cell Signaling Technology, 9271S), AKT (Cell Signaling Technology, 9272S), ERK1/2 (Cell Signaling Technology, 9102S), pERK1/2 (Cell Signaling Technology, 9101S), β-Tubulin (Cell Signaling Technology, 2146 S), Myc (Cell Signaling Technology, 2278S), and Flag (Sigma-Aldrich, F7425) with dilutions from 1:500–1:3000 in the respective blocking solutions. Following the primary antibody incubation, the membranes were incubated with a secondary antibody conjugated with horseradish peroxidase for 1 h. Visualization of the protein bands was achieved using the ECL Plus kit (Thermo Fisher Scientific, Waltham, MA, USA).

For protein interaction analysis, cellular lysis was performed using a buffer comprising 5 mM EDTA, 50 mM Tris-Cl, 100 mM NaCl, 0.1% NP-40, and a protease inhibitor cocktail (Sigma-Aldrich). Subsequently, cell lysates were subjected to incubation with EZview anti-FLAG-M2, HA affinity beads, or Myc affinity beads (Sigma-Aldrich) at 4 °C for 4 h to facilitate the precipitation of the target proteins. In the case of endogenous proteins, precipitation was achieved by overnight incubation at 4 °C with the corresponding antibodies, followed by further incubation with Protein A/G Sepharose beads TM (GE Healthcare Life Sciences, Marlborough, MA, USA) at 4 °C for 4 h. Following incubation, the precipitates were thoroughly washed three times with lysis buffer to remove any nonspecifically bound proteins. Subsequently, the proteins bound to the beads were eluted using 2x SDS denaturing buffer and subjected to western blotting for further analysis.

### RNA isolation and RT-qPCR

Total RNA was extracted from cultured cells using TRIZOL reagent (Invitrogen, CA, USA). Subsequently, cDNA synthesis was carried out using the EasyScript cDNA Synthesis Kit (Applied Biological Materials Inc., Richmond, Canada). RT-qPCR analysis was conducted on 48 well plates using the Evagreen qPCR master mix reagent (Applied Biological Materials) in a StepOne Real-time PCR system (Applied Biosystems, Foster City, CA, USA). The expression levels were normalized to 18 s ribosomal RNA, which served as an internal control. The primer sequences used were characterized by the following sequences:

18s (FOR: TTCGTATTGAGCCGCTAGA

REV: CTTTCGCTCTGGTCCGTCTT)

IRS1 (FOR: CGCCGCTCAAGTGAGGATTTAAGC,

REV: ATGCATCGTACCATCTACTGATGAGG)

IRS2 (FOR: TGCAGGAGCGACGACTACAT

REV: ATGCGCATGTACCCACTGTC)

### Cell counting and MTT assay

Cells were detached using 0.05% trypsin and 0.02% EDTA and then re-suspended in their respective growth medium. Cell counts were determined by applying an aliquot of the cell suspension to a hemocytometer plate. For the MTT assay, cells were treated with MLN4924 (125 nM) or insulin (100 nM) for a duration of 24 h. Following the treatment, cells were incubated with 0.5 mg/mL of MTT (3-(4,5-dimethylthiazol-2-yl)-2,5-diphenyltetrazolium bromide) at 37 °C under 5% CO_2_ atmospheric condition for 2 h. Subsequently, the medium was removed and the purple formazan precipitate was solubilized using 200 μL of dimethyl sulfoxide. Formazan quantification was performed at 570 nm using spectrophotometry.

### Statistical analysis

All experiments were performed independently on three occasions, and data analysis was conducted using Microsoft Excel 2013, ImageJ, SigmaPlot 10.0, or GraphPad Prism 5 software. The results are expressed as means with corresponding standard deviations. Statistical analysis was conducted using Student’s t-test for general comparisons and the Mann-Whitney U test for comparing protein levels. *P* values less than 0.05 were considered statistically significant. Spearman’s p statistics were utilized to analyze correlations between protein expressions. Statistical significance on figures and supplemental figures is labeled as follow: *** <0.001, ** <0.01, * <0.05 and not significant as ns.

## Results

### Neddylation negatively correlates with cancer progression and insulin stimulus in ovarian cancer

**Fig. 1:**
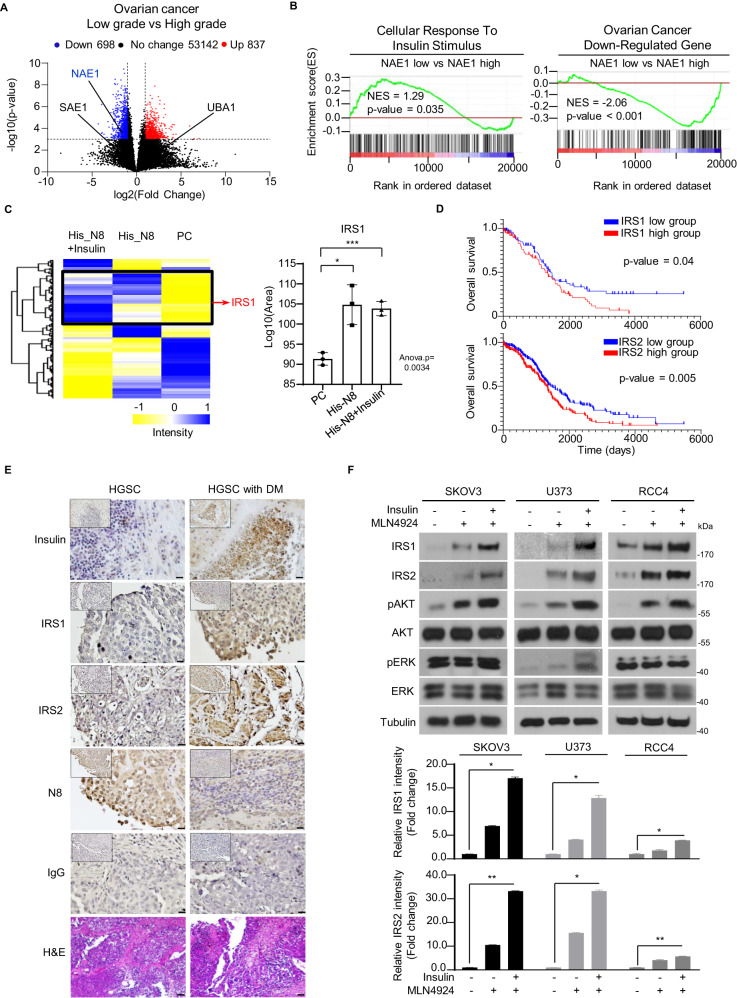
Neddylation is implicated in cancer-associated IRS-dependent insulin signaling. **A** The volcano plot of differential gene expression analysis between low-grade ovarian cancer samples (*n* = 9) and high-grade ovarian cancer samples (*n* = 15). Gene expression data were obtained from GEO (GSE73168). The blue dots represent significantly downregulated genes (log2 Fold Change < −1, −log10 *P* value > 3). The red dots represent significantly upregulated genes (log2 Fold Change > 1, −log10 *P* value > 3). The black dots represent non-significant genes. **B** GSEA was performed with *Cellular response to insulin stimulus* and *Ovarian cancer down-regulated* gene sets using transcriptome data obtained from *NAE1* low groups compared to *NAE1* high groups (GSE73168). These were grouped by conducting *k*-means clustering based on the mRNA levels of NAE1. Normalized enrichment scores(NES) and nominal *P* values are indicated as follows. **C** Hierarchical clustering of quantified proteins among each sample (*p* value < 0.05). Clusters of upregulated proteins are marked in black. Boxplot of relative quantification of IRS1 in each samples. Y axes are represented as relative units. Data were normalized to the total spectral area. (The *p* values were calculated with the student’s t-test. *p < 0.05; ***p < 0.001). **D** The Kaplan-Meier plot shows the overall survival of patients divided into two groups based on the gene expression level of *IRS1* (up panel; positive: *n* = 93; negative: *n* = 94) and *IRS2* (low panel; positive: *n* = 190; negative: *n* = 188). Data sets were obtained from GDC TCGA Ovarian Cancer. Significance was calculated with the two-sided log-rank test. **E** Representative images of immunohistochemistry analysis for Insulin, IRS1, IRS2, and N8 in high-grade serous carcinoma tissues and high-grade serous carcinoma tissues associated with type 2 diabetes mellitus. Scale bar = 100 μm. **F** SKOV3, U373, and RCC4 cell lines were incubated in serum-free media for 24 h, followed by treatment with MLN4924 (125 nM) or insulin (100 nM) for 24 h. The protein levels of IRS1, IRS2, pAKT, AKT, pERK, and ERK were assessed using immunoblot analysis. Quantification of IRS1 and IRS2 immunoblot analysis involved normalizing protein levels to tubulin and expressing them as fold changes relative to the control. Bars represent the means ± SD (*n* = 3). *P < 0.05 ns, not significant.

Given the established link between insulin dysregulation and poor prognosis across different cancer types, our study aimed to elucidate the effects of insulin signaling dysregulation in different tumor contexts. Specifically, we examined ovarian cancer, known for its aggressive behavior associated with insulin signaling dysfunction [24]. To better understand the molecular mechanisms underlying the malignancy of ovarian cancer, we conducted a thorough analysis of post-transcriptional modifications by polypeptide modifiers. By analyzing GSE73168 datasets of low- and high-grade ovarian cancer, we generated volcano plots to identify significant differences in mRNA levels. We observed a marked increase in the mRNA levels of NAE1, E1 enzyme of neddylation, in low-grade ovarian cancer compared to high-grade ovarian cancer, while genes associated with sumoylation (SAE1) and ubiquitination (UBA1) showed no significant differences (Fig. 1A). To gain deeper insights, we conducted GSEA using data from the TCGA database, confirming a strong correlation between lower NAE1 expression and higher insulin stimulus. Notably, we observed that ovarian cancer down-regulated genes were less affected by decreased NAE1 levels, implicating neddylation in insulin signaling with potential implications for poor prognosis in ovarian cancer patients (Fig. 1B). These compelling results underscore the critical involvement of neddylation in insulin signaling and emphasize the need for further investigations to uncover the underlying mechanisms.

### Increased levels of IRS proteins are associated with poor prognosis in ovarian cancer patients

To explore the role of neddylation in insulin signaling, we performed LC-MS/MS analysis to identify relevant proteins. By conjugating His-tagged-NEDD8 in SKOV3 ovarian cancer cells with and without insulin treatment, we identified differentially expressed proteins (DEPs) in the PC vs His-NEDD8 and PC vs His-NEDD8+insulin comparisons. We detected 420 and 478 differentially expressed proteins, respectively, with an overlap of 277 proteins between the two comparisons (Fig. S1A). To gain insights into the functional implications of these DEPS, we conducted pathway analysis using KEGG database, focusing on cancer-related pathways, insulin pathways, and type 2 diabetes mellitus pathways. Notably, this analysis revealed the significance of IRS1 as a critical player in the enrichment of biological functions. Furthermore, hierarchical clustering analysis demonstrated distinct protein expression patterns, with IRS1 displaying significantly increased levels in the His-NEDD8 and His-NEDD8+insulin samples compared to the PC sample (*p* value < 0.05) (Fig. 1C). Given the similarity in structural and functional features between IRS1 and IRS2, we also investigated the potential role of IRS2 neddylation in the insulin signaling cascade. Analysis of data sets from GDC TCGA Ovarian Cancer confirmed the amplification of both IRS1 and IRS2 genes, associated with decreased overall survival rates in ovarian cancer patients (Fig. 1D). We further investigated the clinical relevance by analyzing the expressions of Insulin, IRS1, IRS2, and NEDD8 in ovarian cancer tissues from patients with or without type 2 diabetes mellitus. The results revealed a contrasting pattern, with reduced NEDD8 expression in patients with type 2 diabetes mellitus, accompanied by an induction of IRS1 and IRS2 expression levels (Fig. 1E).

### Prolonged insulin stimuli aggravates cancer cell migration via IRS1 and IRS2 activation under neddylation blockade

Specifically, we examined ovarian cancer, along with glioblastoma and renal cancer, which have received comparatively less attention in the context of insulin signaling dysfunction [8]. Expanding on our investigation, we included glioblastoma (U373) and renal cancer (RCC4) cell lines in our study. Under optimized conditions of MLN4924 (125 nM) and insulin (100 nM) (Fig. S1B), we examined the effects of neddylation inhibition and prolonged insulin secretion on the expression levels of IRS1 and IRS2. The results demonstrated that treatment with MLN4924 led to an increase in IRS1 and IRS2 expression, while co-treatment with MLN4924 and insulin further enhanced their expression levels (Fig. 1F). Given that IRS1 and IRS2 are known to activate key signaling pathways involved in cancer progression, such as the PI3K/AKT and ERK/MAPK pathways, we also examined the protein levels of AKT phosphorylation (pAKT)(S473) and ERK phosphorylation (pERK)(T202/204). While the total protein levels remained unchanged, we observed a parallel increase in pAKT levels in line with the induction of IRS1 and IRS2 expression. However, pERK did not exhibit significant changes. Based on our previous findings demonstrating the impact of neddylation on cancer cell migration through the PI3K/AKT pathway, we hypothesized that neddylation and IRS proteins may influence insulin resistance and cancer cell migration by regulating the PI3K/AKT signaling pathway.

**Fig. 2:**
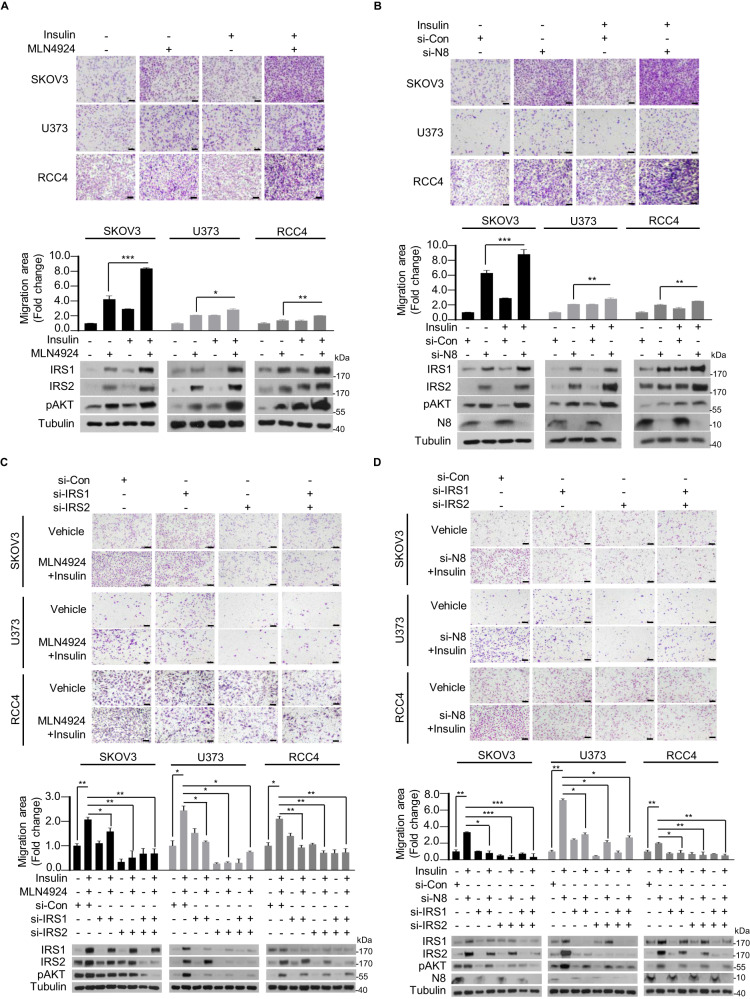
Neddylation of IRS1 and IRS2 suppresses cancer cell migration. **A** SKOV3, U373, and RCC4 cell lines were subjected to Transwell migration assay with or without MLN4924 (125 nM) or insulin (100 nM) treatment. Cell lysates were subjected to immunoblot analysis and protein levels of IRS1, IRS2, and pAKT were assessed. **B** si-Control (si-Con) or si-N8 transfected cells were subjected to Transwell migration assay with or without insulin. Cell lysates were analyzed through immunoblot to verify protein levels of IRS1, IRS2, pAKT and N8. **C** si-Con, si-IRS1 or si-IRS2 transfected cells were subjected to Transwell migration assay with or without MLN4924 or insulin treatment. Cell lysates were subjected to immunoblot analysis and protein levels of IRS1, IRS2, and pAKT were assessed. **D** si-Con, si-N8, si-IRS1, or si-IRS2 transfected cells were subjected to Transwell migration assay with or without insulin treatment. Cell lysates were analyzed through immunoblot to verify protein levels of IRS1, IRS2, pAKT and N8. In each Transwell migration assays, the number of cells in four randomly chosen fields was counted and presented as the means ± SD (*n* = 3). Scale bar represents 100 μm. *P < 0.05; **P < 0.01, ***P < 0.001, *ns* not significant.

We then proposed that increase in IRS1 and IRS2 expression levels promotes cell migration in the presence of insulin treatment-associated neddylation impairment. Having established that the simultaneous application of insulin and MLN4924 has no impact on cancer cell proliferation (Fig. S1C, S1D), we proceeded to conduct cell migration assays using three types of cancer cell lines (SKOV3, U373, and RCC4) treated with MLN4924 or insulin (Fig. 2A, Fig. S2A). Extended treatment with insulin remarkably enhanced cell migration compared to neddylation blockade alone, indicating a synergistic effect. This effect was further validated using NEDD8-targeting siRNA (si-NEDD8) (Fig. 2B, Fig. S2B). Consistent with the observed increase in cell migration, the expression levels of IRS1, IRS2, and pAKT were also elevated. To verify the direct role of IRS1 and IRS2 in cell migration through the PI3K/AKT pathway, we conducted cell migration assays using MLN4924 or si-NEDD8, along with IRS1 and IRS2 targeting si-RNAs (si-IRS1, si-IRS2) by transwell and wound healing assay. Knockdown of either IRS1 or IRS2 significantly reduced the migration effect induced by MLN4924 across all three cancer cell lines (Fig. 2C, Fig. S2C). Similarly, co-transfection of si-NEDD8 and si-RNAs targeting IRS1 and IRS2 resulted in a marked decrease in the migratory effect (Fig. 2D, Fig. S2D). Considering the possibility of IRS1 and IRS2 compensating for each other’s deficiency, we conducted co-transfection of si-IRS1 and si-IRS2 with either si-NEDD8 or treatment with MLN4924. While the double-knockdown of IRS1 and IRS2 led to a decrease in migration effect, it was evident that IRS1 and IRS2 have independent impacts on migration, not solely reliant on each other. Furthermore, the impact of IRS1 and IRS2 on the expression levels of pAKT was validated, showing a reduction. Hence, these findings highlight the critical role of IRS1 and IRS2 neddylation in facilitating cancer cell migration under conditions of insulin signaling dysfunction.

### Insulin and neddylation blockade synergistically activates IRS1 and IRS2 protein stability

**Fig. 3:**
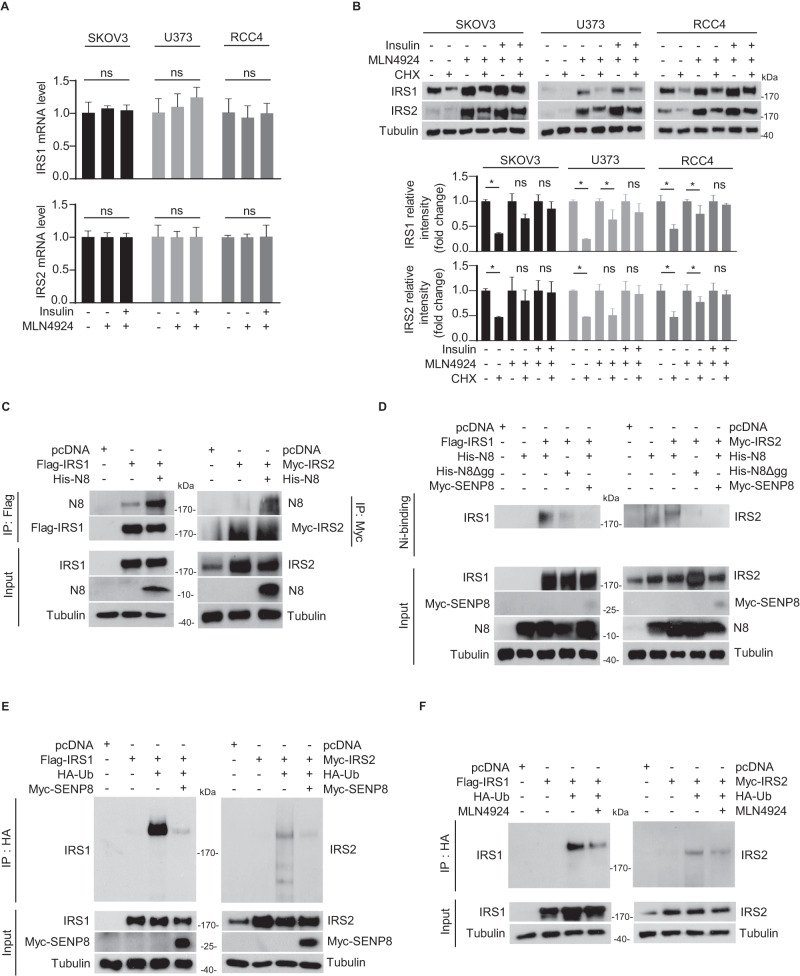
Neddylation of IRS1 and IRS2 is required for their ubiquitination-mediated degradation. **A** SKOV3, U373, and RCC4 cell lines were pre-incubated with serum-free media for 24 h, followed by treatment with MLN4924 (125 nM) or insulin (100 nM) for an additional 24 h. The mRNA levels of IRS1 and IRS2 were analyzed using RT-qPCR. The data are presented as the means ± SD (*n* = 3) and ns indicates “not significant”. **B** All three cancer cell lines were pre-incubated with or without MLN4924 or insulin, followed by incubation with 100 μM cycloheximide (CHX) for 8 h. Cell lysates were subjected to immunoblotting using the indicated antibodies. Band intensities (means ± SD, *n* = 3) on the blots were analyzed using ImageJ and plotted (lower panel). *P < 0.05, *ns* not significant. **C** HEK293T cells were co-transfected with plasmids expressing Flag-IRS1, Myc-IRS2, or His-N8. Cell lysates were subjected to immunoprecipitation using Flag or Myc beads, followed by immunoblotting with the respective target-specific antibodies. **D** Flag-IRS1, Myc-IRS2, His-N8, His-N8ΔGG, or Myc-SENP8 plasmids were co-transfected into HEK293T cells. His-N8 or His-N8ΔGG was pulled down with Ni^2+^ beads under denaturing conditions and then immunoblotted with the target-specific antibodies (IRS1 or IRS2). **E** HEK293T cells were co-transfected with plasmids expressing Flag-IRS1, Myc-IRS2, HA-Ub, or Myc-SENP8. Cell lysates were analyzed by immunoprecipitation using HA beads. Immunoprecipitated samples were then immunoblotted with target-specific antibodies (IRS1 or IRS2). **F** HEK293T cells were co-transfected with plasmids expressing Flag-IRS1, Myc-IRS2, or HA-Ub, and subsequently treated with or without MLN4924. Cell lysates were subjected to immunoprecipitation using HA beads, followed by immunoblotting with the respective target-specific antibodies (IRS1 or IRS2).

Since insulin treatment and neddylation impairment synergistically increased the expression levels of both IRS1 and IRS2, we assessed the gene expression to unravel the molecular mechanism underlying the process. Despite the induction in protein levels, MLN4924 treatment and co-treatment with insulin did not impact the mRNA expression levels of IRS1 and IRS2 (Fig. 3A). Following the disengagement of the transcriptional process, we proceeded to verify the stability of IRS1 and IRS2 proteins by conducting cycloheximide (CHX) treatment. Having established the half-life of IRS1 and IRS2 proteins, we proceeded with the optimized conditions and prioritized investigating the impact of insulin on the stability of IRS1 and IRS2 under neddylation blockade (Fig. 3B, Fig. S3A). Co-treatment with insulin resulted in an even greater induction in stability, surpassing the effect observed with MLN4924 treatment alone. Collectively, these findings suggest that irregularities in insulin signaling significantly contributes to the degradation of IRS1 and IRS2 proteins in the context of neddylation blockade.

### Neddylation of IRS1 and IRS2 is essential for ubiquitination-mediated degradation at protein levels

Drawing upon the precedent of neddylation preceding ubiquitination in c-Src [17], we inferred a similar sequential relationship for IRS1 and IRS2, both known to undergo ubiquitination [12, 25]. To establish the sequential event, we first confirmed the presence of neddylation in IRS1 and IRS2 proteins. Immunoprecipitation assays were carried out in HEK293 cell lysates following the transfection of His-NEDD8 with Flag-IRS1 or Myc-IRS2 (Fig. 3C). To further investigate the covalent conjugation between NEDD8 with IRS1 and IRS2, we conducted a pull-down assay using Ni^2+^ affinity beads. Co-transfection of Flag-IRS1 or Myc-IRS2 with His-NEDD8, His-NEDD8ΔGG (a conjugation-defective mutant), or Myc-SENP8 (a deneddylating enzyme) provided additional evidence of the binding between NEDD8 and IRS1 and IRS2 proteins (Fig. 3D). Then, we investigated the sequential interplay between neddylation and ubiquitination of IRS1 and IRS2 by employing Myc-SENP8 and MLN4924. We utilized the selective capabilities of SENP8 to deneddylate IRS1 and IRS2 while preserving ubiquitinated proteins [26]. Immunoprecipitation assays were performed on samples co-transfected with HA- ubiquitin (Ub) and Myc-SENP8, along with Flag-IRS1 or Myc-IRS2 (Fig. 3E). These experiments demonstrated a reduction in the levels of ubiquitinated IRS1 and IRS2 proteins, which was further confirmed by using MLN4924 (Fig. 3F). These findings strongly suggest that neddylation acts as the primary step preceding the ubiquitin-dependent proteasomal degradation of IRS1 and IRS2, providing valuable insights into the regulatory role of neddylation in stabilizing IRS1 and IRS2 proteins in cellular processes.

### C-CBL engages IRS1 and IRS2 neddylation as an E3 ligase performing tumor suppressive roles

**Fig. 4:**
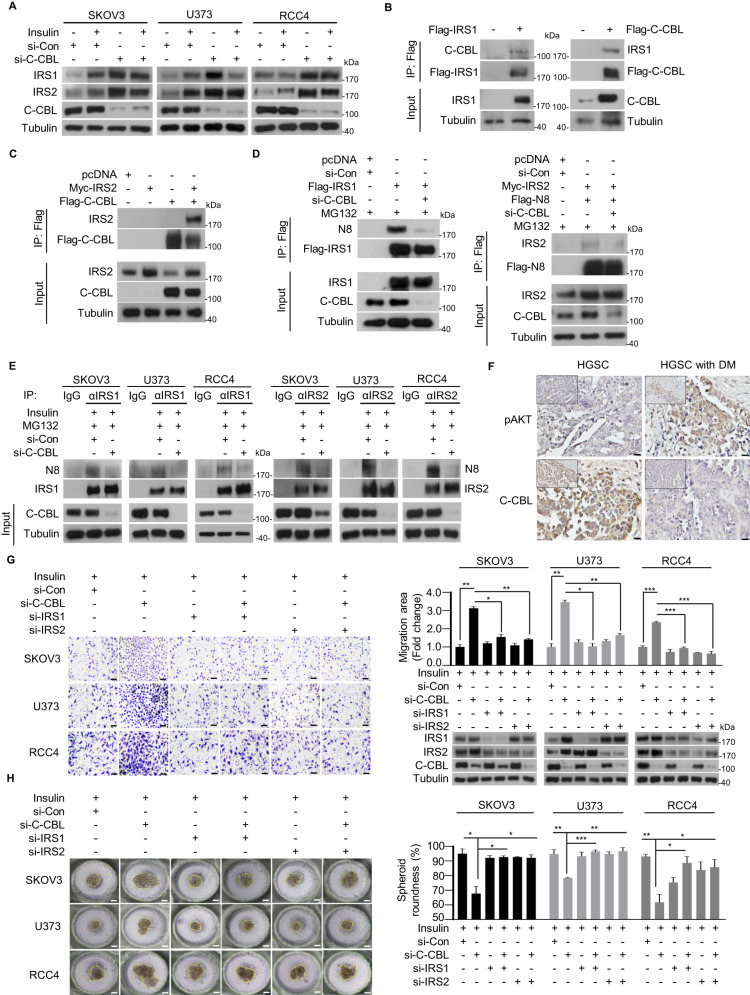
C-CBL functions as a tumor suppressor by facilitating IRS1 and IRS2 neddylation. **A** SKOV3, U373, and RCC4 cell lines were transfected with the si-Con and si-C-CBL, and then treated with or without insulin (100 nM). The protein levels were analyzed using immunoblot. **B** Flag-IRS1 or Flag-C-CBL plasmids were transfected into HEK293T cells and lysates were immunoprecipitated using Flag beads. The results were analyzed by immunoblotting using antibodies specific to C-CBL or IRS1. **C** HEK293T cells were transfected with either Myc-IRS2 or Flag-C-CBL plasmids. Cell lysates were immunoprecipitated using Flag beads, and the immunoblotting analysis was performed using an IRS2-specific antibody. **D** HEK293T cells were co-transfected with si-C-CBL and Flag-IRS1, Myc-IRS2, or Flag-N8 and subsequently treated with MG132 (10 μM). Cell lysates were subjected to immunoprecipitation using Flag beads. The results were analyzed by immunoblotting using target-specific antibodies (N8 or IRS2). **E** Following transfection with si-C-CBL and subsequent treatment with MG132, all three cancer cell lines were subjected to endogenous immunoprecipitation using the indicated target antibodies (IRS1 or IRS2). The immunoblot analysis was performed using the N8-specific antibody. **F** Representative images of immunohistochemistry analysis for pAKT and C-CBL in high-grade serous carcinoma tissues and high-grade serous carcinoma tissues associated with type 2 diabetes mellitus. Scale bar = 100 μm. **G** si-Con, si-C-CBL, si-IRS1, or si-IRS2 co-transfected cancer cells were subjected to Transwell migration assay with or without insulin. Cell lysates were analyzed through immunoblot to verify protein levels of IRS1, IRS2, and C-CBL. The number of cells in four randomly chosen fields was counted and presented as the means ± SD (*n* = 3). The scale bar represents 100 μm. *P < 0.05; **P < 0.01, ***P < 0.001. **H** All three cancer cell lines co-transfected with si-Con, si-C-CBL, si-IRS1, or si-IRS2 were incubated in PDMS 3D culture chips with a culture medium containing vehicle or insulin. Representative optical microscopy images were obtained on day 5. The average diameter of the spheroids was measured using ImageJ, and the results are presented as the means ± SD (*n* = 3). The scale bar represents 100 μm. *P < 0.05; **P < 0.01, ***P < 0.001.

**Fig. 5:**
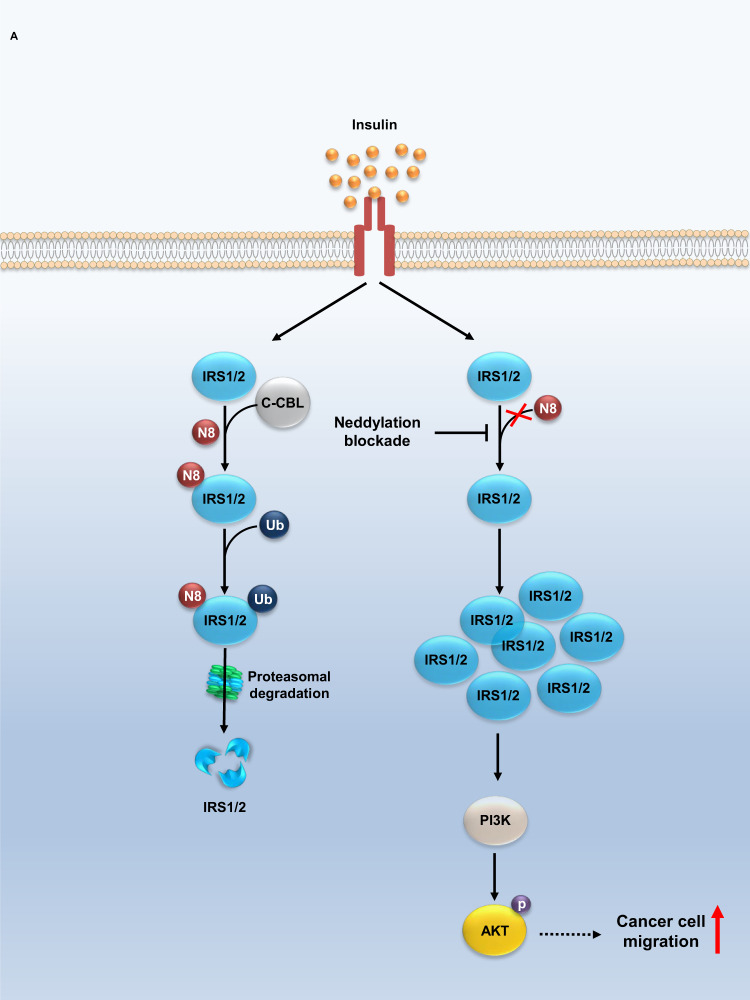
The E3 ligase activity of C-CBL is implicated in insulin signaling and cancer cell migration through IRS neddylation. **A** A proposed model illustrating the role of IRS neddylation in insulin signaling and cancer migration, involving C-CBL and the PI3K/AKT pathway.

Building upon our proteomics data revealing the binding of NEDD8 to C-CBL (data not shown) and our prior discovery of C-CBL’s E3 neddylation ligase activity towards c-Src [17], we postulated a comparable role for C-CBL in modulating the activity of both IRS1 and IRS2. To investigate this, we first validated the endogenous expression levels of IRS1 and IRS2 in all three types of cancer cell lines by using C-CBL targeting si-RNA (si-C-CBL) under prolonged insulin treatment. We observed an increase in both IRS protein expression levels upon C-CBL knockdown (Fig. 4A). Immunoprecipitation experiments in HEK293 cells using ectopically expressed Flag-IRS1, Myc-IRS2 or Flag-C-CBL confirmed the interaction between IRS1 and IRS2 with C-CBL (Fig. 4B, C). Furthermore, we validated a reduction in the interaction between IRS1 and NEDD8, as well as IRS2 and NEDD8, upon C-CBL knockdown (Fig. 4D), indicating the functional role of C-CBL as a neddylation E3 ligase for IRS1 and IRS2. We then validated the endogenous interaction between NEDD8 and IRS1 as well as IRS2 in all three cancer cell lines, affirming the neddylation E3 ligase function of C-CBL (Fig. 4E). Notably, ovarian patients with type 2 diabetes mellitus exhibited reduced C-CBL expression, along with induction in pAKT levels (Fig. 4F). Migration assays conducted with si-C-CBL and si-IRS1 or si-IRS2 along with prolonged insulin treatment, demonstrated that C-CBL knockdown enhanced migration by elevating IRS1 and IRS2 expression levels, while knockdown of IRS1 and IRS2 reduced the migration effect (Fig. 4G, Fig. S4A). To mimic in vivo tumor conditions, we utilized a polydimethylsiloxane (PDMS) three-dimensional spheroid culture system and evaluated cell migration by measuring spheroid roundness on day 5, as described previously [27]. Knockdown of C-CBL promoted cell migration, as evidenced by the altered spheroid roundness, while knockdown of IRS1 and IRS2 rescued the effect, resulting in a more rounded spheroid morphology (Fig. 4H). Collectively, these findings support the negative role of C-CBL in cancer cell migration and suggest its potential tumor suppressive function in cancers characterized by dysregulation in insulin receptor substrates 1 and 2 (Fig. 5).

## Discussion

When considering the relationship between insulin resistance and hyperinsulinemia, the question of which factor precedes the other and drives the associated effects remains a topic of debate [28]. Nonetheless, dysfunction in insulin signaling has been emerging as a prominent contributor to deterioration in cancer progression [29]. Various experimental, epidemiological, and clinical evidence support the notion that insulin dysregulation plays a role in cancer progression. For instance, non-fasting insulin concentrations have shown a positive correlation with the size of colonic aberrant crypt foci [30]. Moreover, continuous efforts to replace insulin with insulin analogs emphasize the significance of understanding the mechanisms of irregularities in insulin signaling and their potential impact on cancer mortality risk [31]. In many cancers, dysregulation of insulin receptors (INSR) is a common occurrence, with insulin resistance in metabolic tissues often leading to downregulation of INSR expression. Reports have linked the upregulation of genes encoding INSR with the dysfunction of tumor suppressor genes. In response to insulin through INSR, ligand activation of INSR initiates signaling via the IRS-PI3K-AKT pathway and MAPK pathway [32]. This has prompted a growing number of reports investigating the role of IRS proteins in mediating insulin response, not only in the context of metabolic disorders but also in relation to cancer.

IRS proteins often play significant roles in the dysfunction of the insulin cascade. Activation by insulin leads to the phosphorylation of tyrosine residues in the C-termini of IRS proteins, generating binding sites that recruit downstream effectors and propagate the signaling response [10, 11]. These effectors, such as PI3K and Grb-2, play crucial roles in diverse cancer-associated processes, encompassing cell survival, proliferation, and migration [33, 34]. IRS1 is frequently upregulated in malignant epithelial ovarian tumors, and its overexpression in the mouse mammary gland leads to tumorigenesis through tyrosine phosphorylation, activating AKT and ERK1/2 [35, 36]. It is critical to note that the inhibition of IRS1 and IRS2 can have repressive effects on malignant behaviors observed in ovarian cancers and glioblastoma cell proliferation [37, 38]. While IRS2 has traditionally been associated more with cell migration than proliferation, recent reports highlight the significant role of IRS1 in promoting migration as well. Studies have shown that malignant behaviors, including migration and invasion, of ovarian cancers depend on both IRS1 and IRS2 through the activation of the PI3K/AKT pathway [37]. Moreover, overexpression of IRS1 has been found to promote migration in cholangiocarcinoma and regulate invasion in pancreatic cells via both MAPK and PI3K signaling pathways [39]. These findings suggest that overexpression of both IRS1 and IRS2 emerges as crucial factors in cancer cell migration, warranting a more comprehensive understanding of the precise mechanism by which insulin dysfunction-dependent activated IRS proteins contribute to cancers.

Neddylation, a complex enzymatic process, orchestrates a symphony of molecular events in diverse biological processes. Beginning with the neddylation-activating enzyme E1 (NAE1), which comprises APPBP1 and UBA3, NEDD8 is activated and subsequently transferred to target substrates via the E2 enzyme UBC12 and E3 ligases [40]. While cullin proteins have been extensively studied as neddylation targets, there is growing interest in understanding the role of neddylation in non-cullin proteins, which reveals additional biological functions. Of particular interest are the contrasting effects of neddylation on protein stability, as it exhibits both ubiquitination-dependent and ubiquitination-independent effects on distinct substrates [41]. In some cases, neddylation enhances protein stability by preventing ubiquitin-dependent proteasomal degradation, as exemplified by murine double minute-2 protein (MDM2), peroxisome proliferator-activated receptor gamma (PPARγ), and sterol regulatory element-binding protein 1c (SREBP1c) [20, 42, 43]. On the other hand, neddylation facilitates the degradation of specific substrates, such as c-Src, epidermal growth factor (EGFR), and histone deacetylase 2 (HDAC2), by facilitating enhanced ubiquitination and subsequent proteasomal degradation [17, 44, 45]. Neddylation is also involved in ubiquitination-independent degradation processes, as observed with substrates including HIF-1α, HIF-2α, E2F, PB2, and SRSF3 [46–49].

The role of IRS posttranslational modifications in cancer progression is not fully understood. Limited information is available on this topic, with only two reports specifically linking IRS modifications to cancer activity. For instance, TRAF4 has been reported to mediate IRS1 ubiquitination, regulating IRS1 binding with IGF-1R and subsequent IRS1 phosphorylation, leading to the stimulation of proliferation in breast cancer cells [50]. Also, the association of Nedd4 and IRS2 through monoubiquitination has been reported to activate IGF-1 signaling and induce cell proliferation in prostate cancer cells [25]. Our findings regarding the neddylation of IRS1 and IRS2 align with the observed pattern of neddylation promoting degradation through enhanced ubiquitination and subsequent proteasomal degradation. While previous studies have primarily focused on cullin-dependent neddylation of IRS1 protein, our study reveals a novel aspect by demonstrating neddylation of IRS1 and IRS2 mediated by a non-cullin E3 ligase, C-CBL [12, 51, 52]. While Cbl-b, another member of the Cbl family, is known to regulate IRS1 as a ubiquitination E3 ligase in unloading-induced muscle atrophy, the specific role of C-CBL with IRS proteins in the context of cancer remains unexplored. In contrast, C-CBL has been identified as a significant proto-oncogene in various cancers and linked to the downregulation of PDK1 in EGFR wild-type non-small cell lung cancers, suggesting its potential role in immune responses [53]. Moreover, our previous research has revealed C-CBL’s tumor suppressive role as an E3 ligase for c-Src neddylation [17]. Considering the diverse proto-oncogenic roles of the Cbl family and the critical roles of the IRS family in insulin signaling, we speculate that C-CBL may play an important role in mediating IRS protein neddylation, which could have implications for cancer patients with T2DM or hyperinsulinemia.

Our research findings indicate that C-CBL mediated neddylation acts as an antagonist against insulin-associated cancer cell migration involving IRS1 and IRS2 in diverse cancer types. Amidst the growing interest in neddylation as a promising therapeutic target for cancer treatments, it is significant to emphasize that our research findings do not contradict the well-established anticancer effects of MLN4924, a known neddylation inhibitor [54]. As we advance in our understanding of the intricate interplay between insulin dysregulation and neddylation in cancer progression, careful evaluation of neddylation inhibitors in cancer patients with T2DM or hyperinsulinemia is necessary.

### Supplementary information

Supplementary Figures

Dataset 1

Dataset 2

Dataset 3

Dataset 4

Supplementary Dataset

## Data Availability

All data are available in the main text or the supplementary materials. The proteomics datasets generated in this study are available via ProteomeXchange with the identifier PXD044760.
