# Supplementary material for: Neddylation of insulin receptor substrate acts as a bona fide regulator of insulin signaling and its implications for cancer cell migration
Source: Cancer Gene Ther. 2024 Jan 25;31(4):599–611. doi: 10.1038/s41417-024-00729-z (PMC11016467; doi:10.1038/s41417-024-00729-z)
Supplement: Supplementary file 1 — Supplementary Figures [file 41417_2024_729_MOESM1_ESM.pdf]

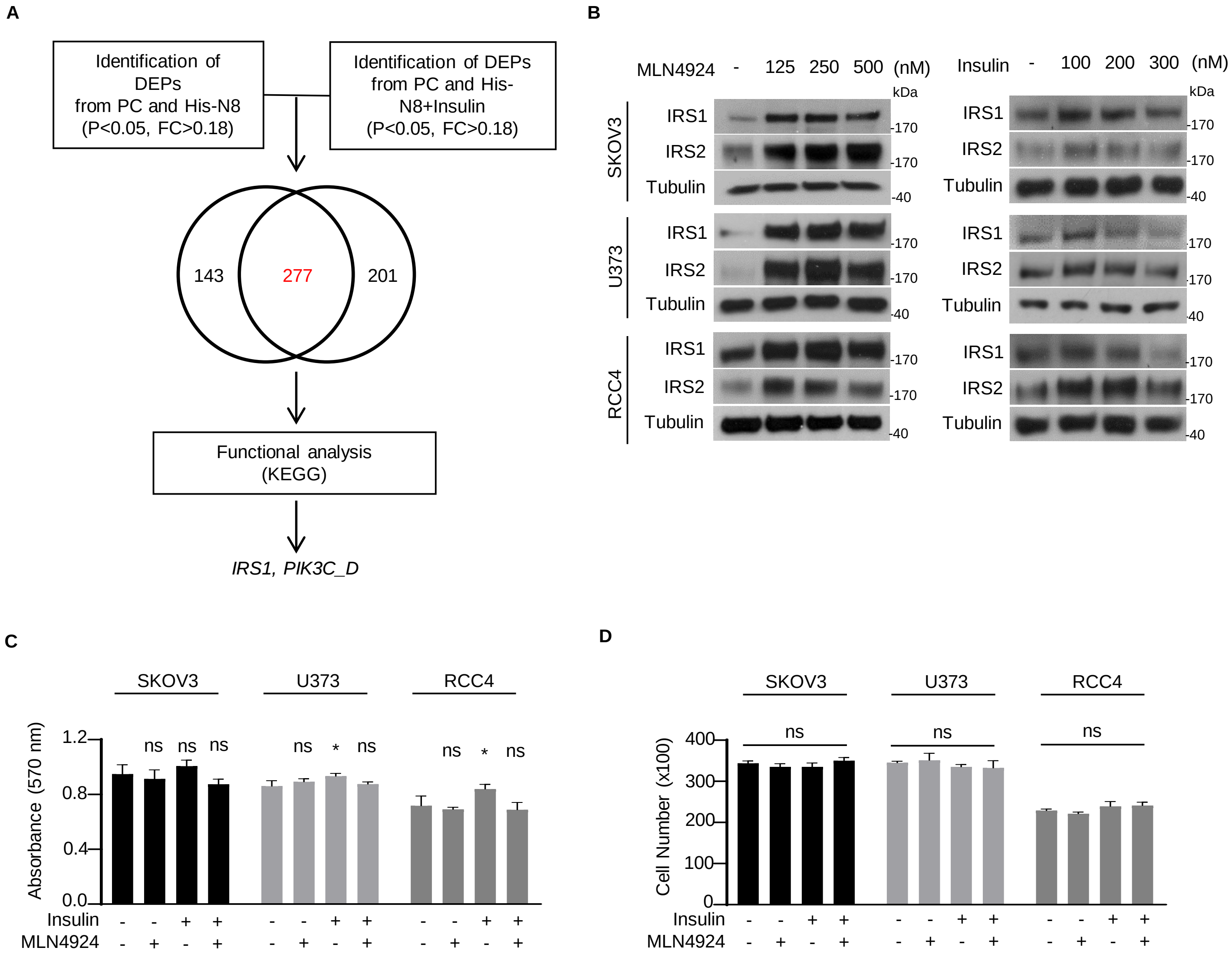

Fig. S1. Prolonged insulin secretion and neddylation blockade does not affect cell proliferation
(A) The flowchart of the selection process for the target protein utilizing KEGG analysis of three independent pathways. Genes shared between the identified DEPs and KEGG analysis are listed under the Venn diagram.
(B) SKOV3, U373, and RCC4 cell lines were pre-incubated with serum-free media for 24 hours, followed by treatment with MLN4924 or insulin for an additional 24 hours with the indicated concentrations. Cell lysates were analyzed with immunoblot with the following antibodies. (C) The cells were pre-incubated with serum-free media for 24 hours then treated with or without 125 nM MLN4924 or 100 nM insulin. After 24 hours, cells were incubated with MTT solution to measure the cell proliferation. (D) Following treatment with either 125 nM MLN4924 or 100 nM insulin, cell counting was performed using a hemocytometer. Bars represent the means ± SD (n=3). *, $P < 0.05$ ns, not significant

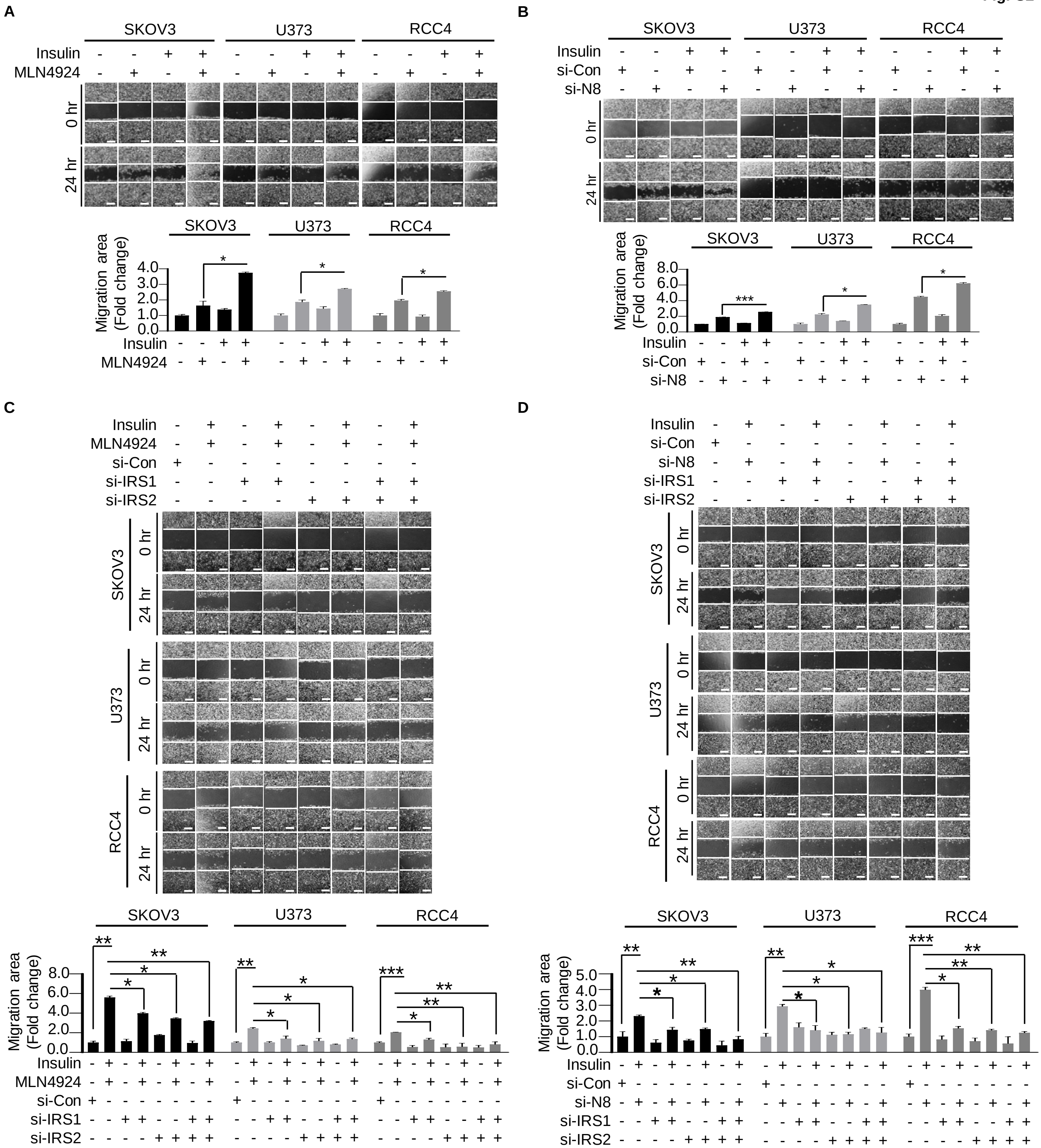

Fig. S2. Neddylation of IRS1 and IRS2 inhibits cancer cell migration under prolonged insulin secretion
(A) SKOV3, U373, RCC4 cell lines were pre-incubated with serum-free media for 24 hours then subjected to wound healing assay with the treatment of either 125 nM MLN4924 or 100 nM insulin. (B) Cells transfected with si-Con or si-N8 were pre-incubated with serum-free media for 24 hours then subjected to wound healing assay with or without treating insulin. (C) si-Con, si-IRS1 or si-IRS2 transfected cells were subjected to wound healing assay with or without MLN4924 or insulin treatment. (D) si-Con, si-N8, si-IRS1, or si-IRS2 transfected cells were subjected to wound healing assay with or without insulin treatment. Whole areas were measured using the ImageJ software and data are presented as the means ± SD (n=3). *, $P < 0.05$; **, $P < 0.01$, ***, $P < 0.001$

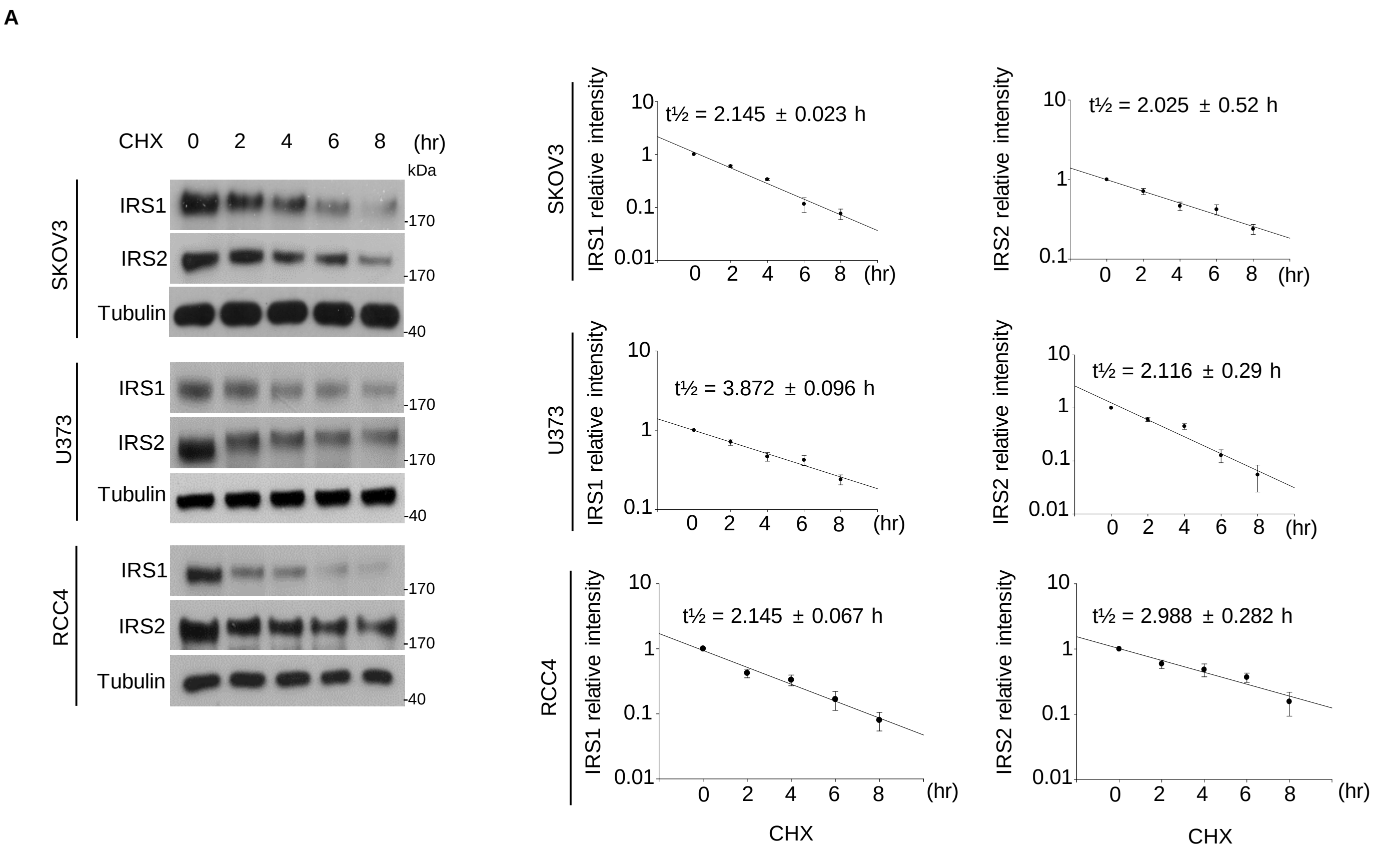

Fig. S3. Half-life of IRS1 and IRS2 in each cancer cell line

(A) SKOV3, U373, RCC4 cell lines were pre-incubated with serum-free medium for 24 hours, followed by incubation with 100 μM cycloheximide (CHX) for the indicated hours. Cell lysates were subjected to immunoblotting using the indicated antibodies. Relative intensities (means ± SD, n=3) on the graph were analyzed using ImageJ and plotted using SigmaPlot (right panel).

A

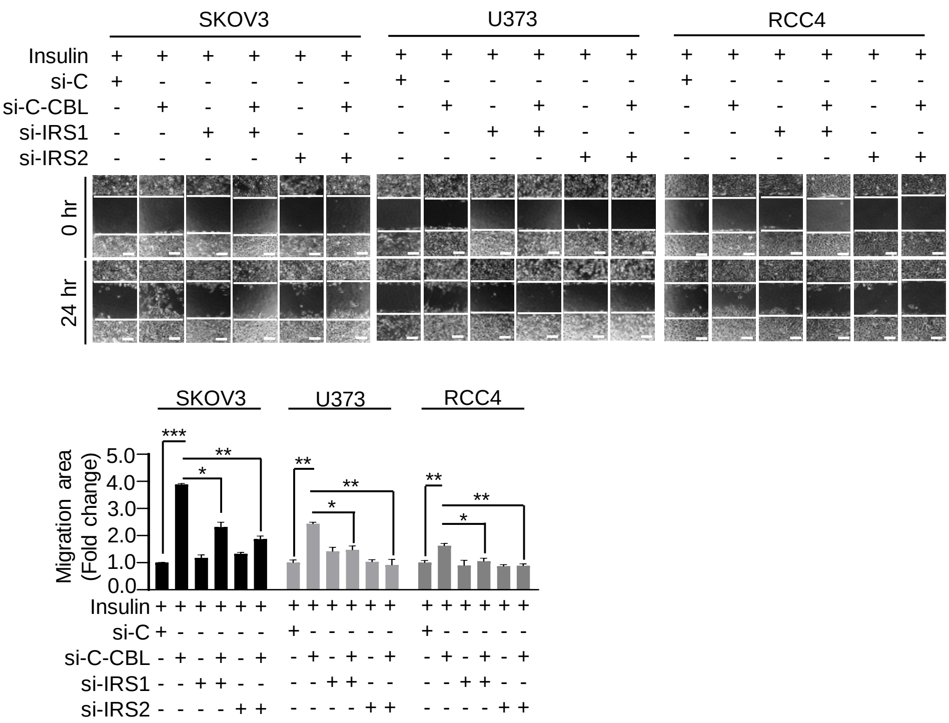

Fig. S4. C-CBL suppresses IRS1 and IRS2 neddylation dependent cancer cell migration

(A) si-Con, si-C-CBL, si-IRS1 or si-IRS2 transfected cell lines were subjected to wound healing assay with or without insulin (100 nM) treatment. Whole areas were measured using the ImageJ software and data are presented as the means ± SD (n=3). *, $P < 0.05$; **, $P < 0.01$, ***, $P < 0.001$

	Pathology ID	Block	T2DM(Y/N)	Gender	Age	Diagnosis	FIGO stage	BMI
1	S 220015098	4	Y	F	71	HGSC	IIIC	25
2	S 210076901	1	Y	F	79	HGSC	IVB	25.8
3	S 210070175	1	Y	F	69	HGSC	IIIC	18.4
4	S 210041057	2	Y	F	60	HGSC	IVB	26.1
5	S 210030216	6	Y	F	72	HGSC	IVB	23.7
6	S 210029848	3	Y	F	71	HGSC	IIIC	21.6
7	S 210023364	2	Y	F	81	HGSC	IA	23.2
8	S 210013371	1	Y	F	88	HGSC	IIIC	24.4
9	S 200063835	13	Y	F	81	HGSC	IVB	17
10	S 200052822	9	Y	F	71	HGSC	IIIC	22.1
11	S 200050299	2	Y	F	77	HGSC	IIB	25.7
12	S 200045400	5	Y	F	55	HGSC	IIIC	22.5
13	S 200032434	A	Y	F	77	HGSC	IVB	29.7
14	S 200021892	1	Y	F	76	HGSC	IC	23.6
15	S 200005918	12	Y	F	58	HGSC	IIIC	24
16	S 220061678	14	N	F	36	HGSC	IIIC	23.9
17	S 220058502	2	N	F	60	HGSC	IIIC	23.5
18	S 220040893	16	N	F	58	HGSC	IVB	24.1
19	S 220038434	2	N	F	54	HGSC	IIA	27
20	S 220038368	2	N	F	73	HGSC	IVB	23.4
21	S 220037773	4	N	F	61	HGSC	IVB	24.5
22	S 220033875	1	N	F	67	HGSC	IIIC	24.8
23	S 220019312	4	N	F	62	HGSC	IIIA2	22.8
24	S 220014909	3	N	F	64	HGSC	IA	23.7
25	S 220012492	2	N	F	55	HGSC	IVB	18.9
26	S 220007754	2	N	F	55	HGSC	IC2	22.9
27	S 220006980	2	N	F	51	HGSC	IIIC	23.6
28	S 220006379	1	N	F	58	HGSC	IIIC	24.3
29	S 210073578	5	N	F	52	HGSC	IIIB	24.6
30	S 210071033	7	N	F	61	HGSC	IIIC	20

Table S1. Clinical information for tissues utilized in immunohistochemistry
