# Supplementary figures and images for: Neddylation of insulin receptor substrate acts as a bona fide regulator of insulin signaling and its implications for cancer cell migration (part 1 of 3)
Source: Cancer Gene Ther. 2024 Jan 25;31(4):599–611. doi: 10.1038/s41417-024-00729-z (PMC11016467; doi:10.1038/s41417-024-00729-z)

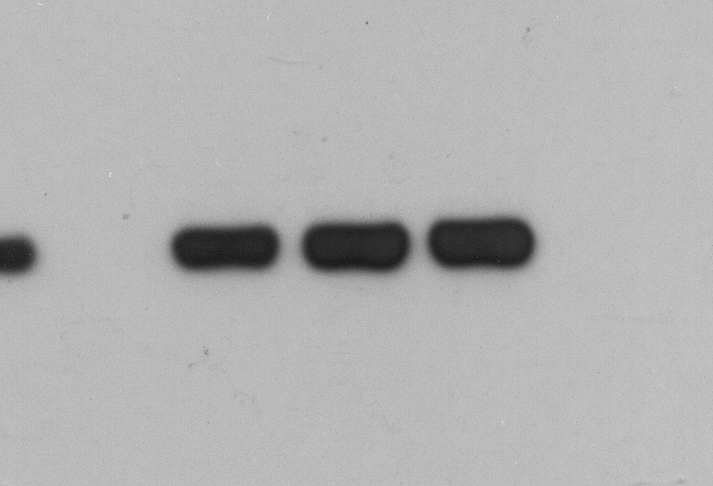

Supplement: Supplementary file 2 — Dataset 1 [file 41417_2024_729_MOESM2_ESM.zip › Dataset 1/Figure 1F/RCC4 AKT.tif]

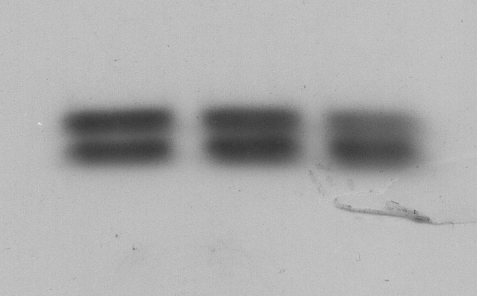

Supplement: Supplementary file 2 — Dataset 1 [file 41417_2024_729_MOESM2_ESM.zip › Dataset 1/Figure 1F/RCC4 ERK.tif]

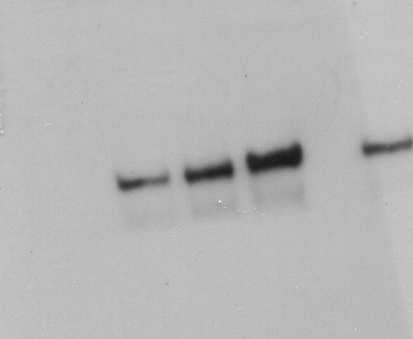

Supplement: Supplementary file 2 — Dataset 1 [file 41417_2024_729_MOESM2_ESM.zip › Dataset 1/Figure 1F/RCC4 IRS1.tif]

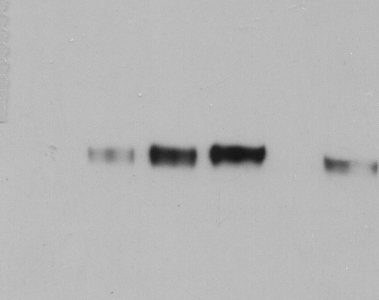

Supplement: Supplementary file 2 — Dataset 1 [file 41417_2024_729_MOESM2_ESM.zip › Dataset 1/Figure 1F/RCC4 IRS2.tif]

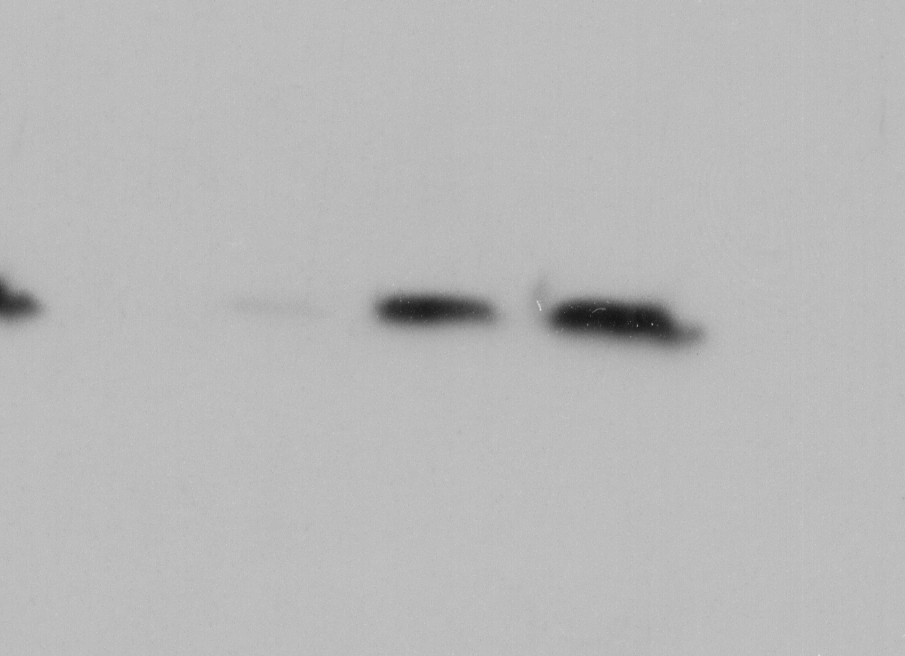

Supplement: Supplementary file 2 — Dataset 1 [file 41417_2024_729_MOESM2_ESM.zip › Dataset 1/Figure 1F/RCC4 pAKT.tif]

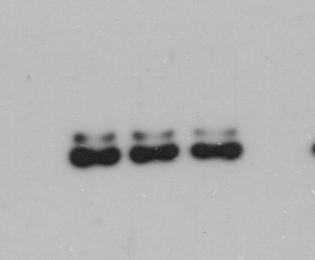

Supplement: Supplementary file 2 — Dataset 1 [file 41417_2024_729_MOESM2_ESM.zip › Dataset 1/Figure 1F/RCC4 pERK.tif]

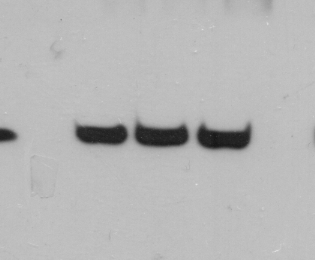

Supplement: Supplementary file 2 — Dataset 1 [file 41417_2024_729_MOESM2_ESM.zip › Dataset 1/Figure 1F/RCC4 TUB.tif]

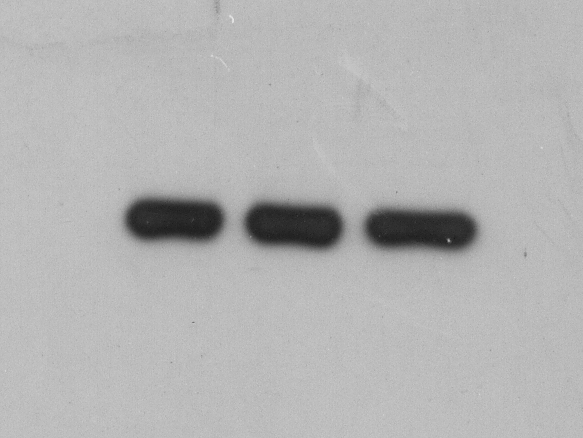

Supplement: Supplementary file 2 — Dataset 1 [file 41417_2024_729_MOESM2_ESM.zip › Dataset 1/Figure 1F/SKOV3 AKT.tif]

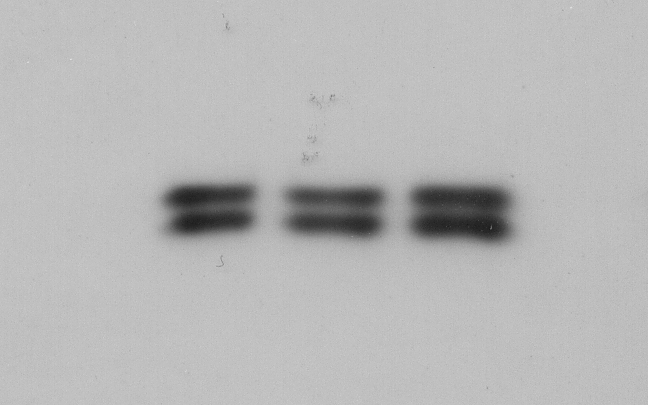

Supplement: Supplementary file 2 — Dataset 1 [file 41417_2024_729_MOESM2_ESM.zip › Dataset 1/Figure 1F/SKOV3 ERK.tif]

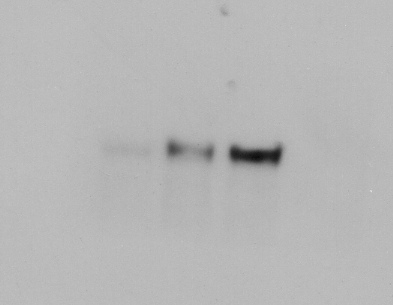

Supplement: Supplementary file 2 — Dataset 1 [file 41417_2024_729_MOESM2_ESM.zip › Dataset 1/Figure 1F/SKOV3 IRS1.tif]

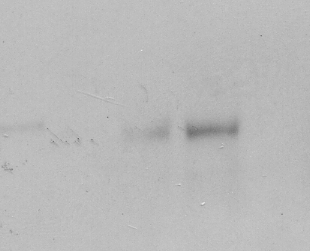

Supplement: Supplementary file 2 — Dataset 1 [file 41417_2024_729_MOESM2_ESM.zip › Dataset 1/Figure 1F/SKOV3 IRS2.tif]

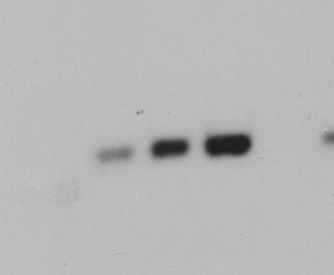

Supplement: Supplementary file 2 — Dataset 1 [file 41417_2024_729_MOESM2_ESM.zip › Dataset 1/Figure 1F/SKOV3 pAKT.tif]

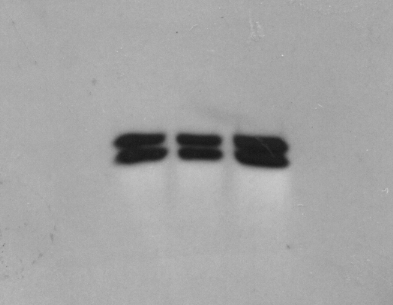

Supplement: Supplementary file 2 — Dataset 1 [file 41417_2024_729_MOESM2_ESM.zip › Dataset 1/Figure 1F/SKOV3 pERK.tif]

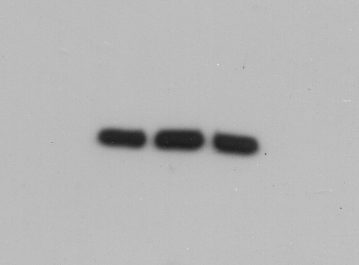

Supplement: Supplementary file 2 — Dataset 1 [file 41417_2024_729_MOESM2_ESM.zip › Dataset 1/Figure 1F/SKOV3 tub.tif]

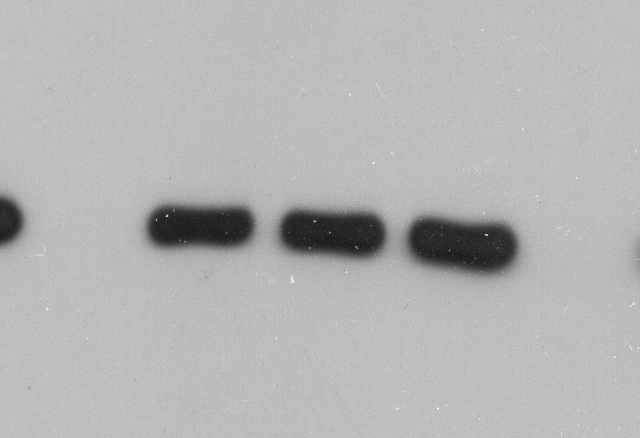

Supplement: Supplementary file 2 — Dataset 1 [file 41417_2024_729_MOESM2_ESM.zip › Dataset 1/Figure 1F/U373 AKT.tif]

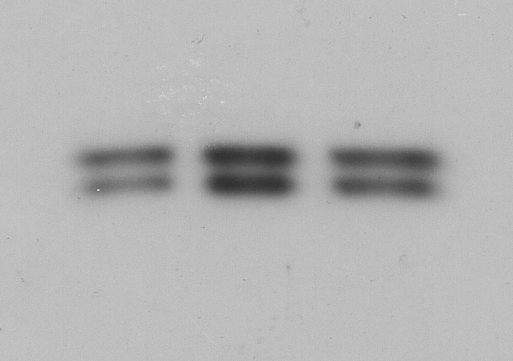

Supplement: Supplementary file 2 — Dataset 1 [file 41417_2024_729_MOESM2_ESM.zip › Dataset 1/Figure 1F/U373 ERK.tif]

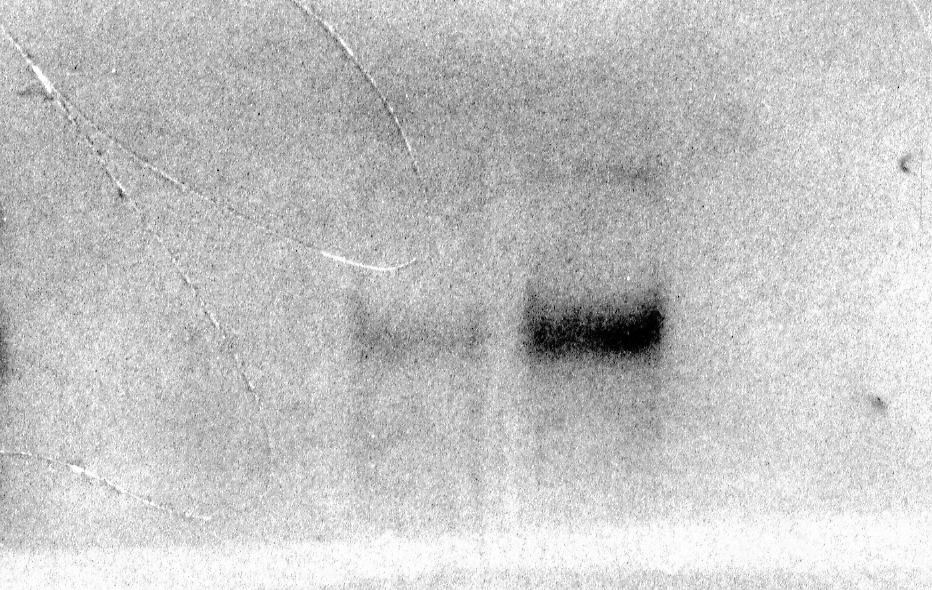

Supplement: Supplementary file 2 — Dataset 1 [file 41417_2024_729_MOESM2_ESM.zip › Dataset 1/Figure 1F/U373 IRS1.tif]

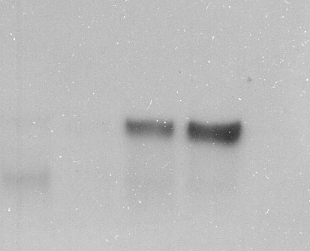

Supplement: Supplementary file 2 — Dataset 1 [file 41417_2024_729_MOESM2_ESM.zip › Dataset 1/Figure 1F/U373 IRS2.tif]

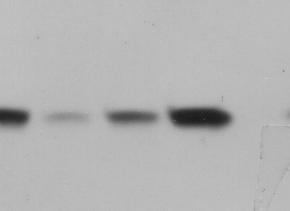

Supplement: Supplementary file 2 — Dataset 1 [file 41417_2024_729_MOESM2_ESM.zip › Dataset 1/Figure 1F/U373 pAKT.tif]

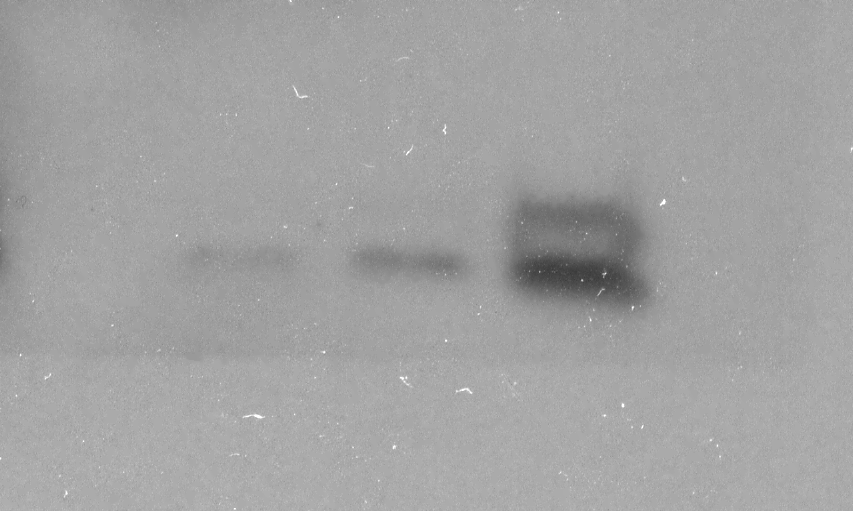

Supplement: Supplementary file 2 — Dataset 1 [file 41417_2024_729_MOESM2_ESM.zip › Dataset 1/Figure 1F/U373 pERK.tif]

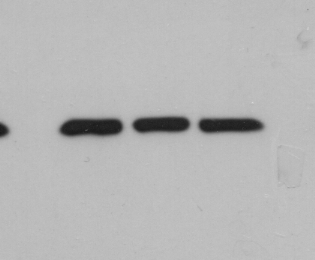

Supplement: Supplementary file 2 — Dataset 1 [file 41417_2024_729_MOESM2_ESM.zip › Dataset 1/Figure 1F/U373 TUB.tif]

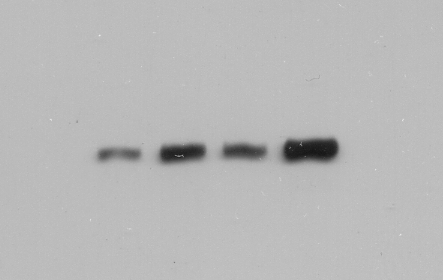

Supplement: Supplementary file 3 — Dataset 2 [file 41417_2024_729_MOESM3_ESM.zip › Dataset 2/Dataset 2/Figure 2/Figure 2a/rcc4/RCC4 IRS1.tif]

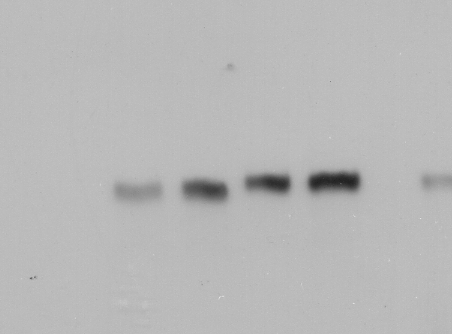

Supplement: Supplementary file 3 — Dataset 2 [file 41417_2024_729_MOESM3_ESM.zip › Dataset 2/Dataset 2/Figure 2/Figure 2a/rcc4/RCC4 IRS2.tif]

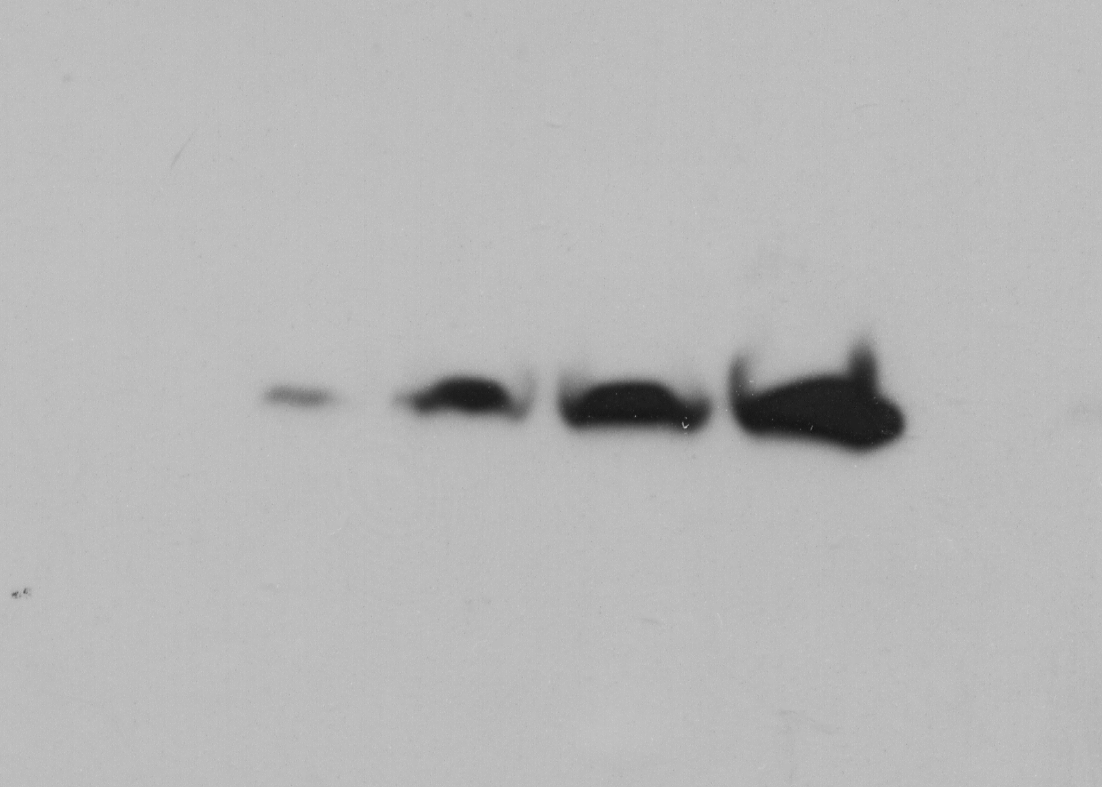

Supplement: Supplementary file 3 — Dataset 2 [file 41417_2024_729_MOESM3_ESM.zip › Dataset 2/Dataset 2/Figure 2/Figure 2a/rcc4/rcc4 pakt.tif]

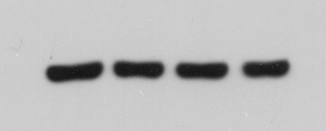

Supplement: Supplementary file 3 — Dataset 2 [file 41417_2024_729_MOESM3_ESM.zip › Dataset 2/Dataset 2/Figure 2/Figure 2a/rcc4/RCC4 tub.tif]

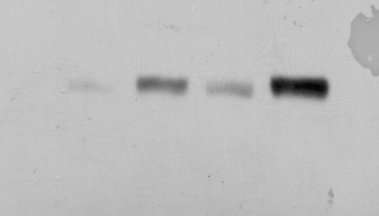

Supplement: Supplementary file 3 — Dataset 2 [file 41417_2024_729_MOESM3_ESM.zip › Dataset 2/Dataset 2/Figure 2/Figure 2a/skov3/SKOV3 IRS1.tif]

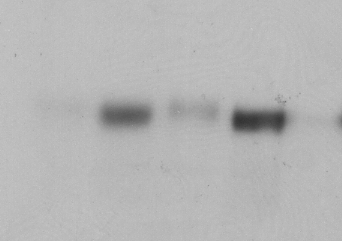

Supplement: Supplementary file 3 — Dataset 2 [file 41417_2024_729_MOESM3_ESM.zip › Dataset 2/Dataset 2/Figure 2/Figure 2a/skov3/SKOV3 IRS2.tif]

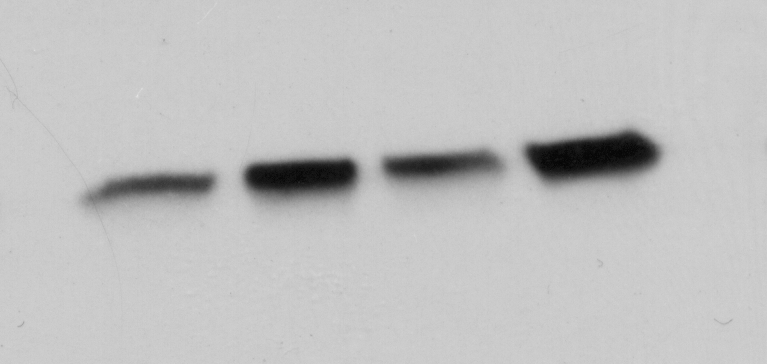

Supplement: Supplementary file 3 — Dataset 2 [file 41417_2024_729_MOESM3_ESM.zip › Dataset 2/Dataset 2/Figure 2/Figure 2a/skov3/SKOV3 pAKT.tif]

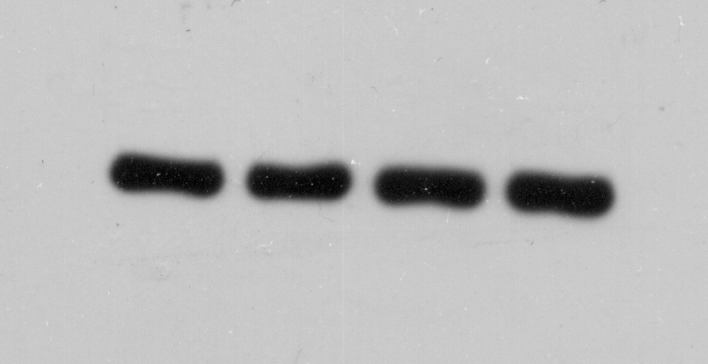

Supplement: Supplementary file 3 — Dataset 2 [file 41417_2024_729_MOESM3_ESM.zip › Dataset 2/Dataset 2/Figure 2/Figure 2a/skov3/SKOV3 TUB.tif]

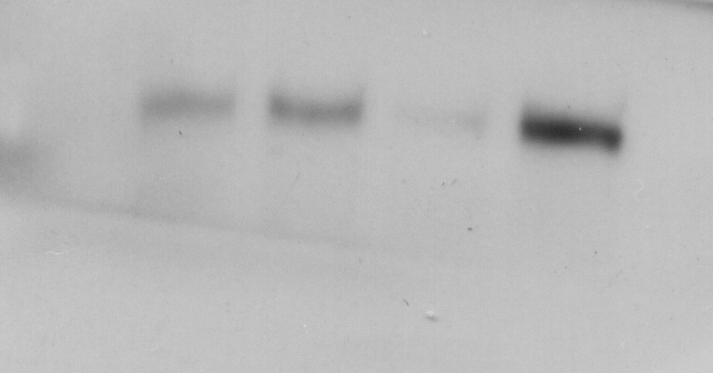

Supplement: Supplementary file 3 — Dataset 2 [file 41417_2024_729_MOESM3_ESM.zip › Dataset 2/Dataset 2/Figure 2/Figure 2a/u373/U373 IRS1.tif]

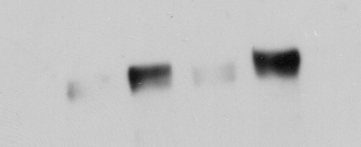

Supplement: Supplementary file 3 — Dataset 2 [file 41417_2024_729_MOESM3_ESM.zip › Dataset 2/Dataset 2/Figure 2/Figure 2a/u373/U373 IRS2.tif]

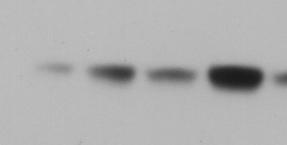

Supplement: Supplementary file 3 — Dataset 2 [file 41417_2024_729_MOESM3_ESM.zip › Dataset 2/Dataset 2/Figure 2/Figure 2a/u373/U373 pakt.tif]

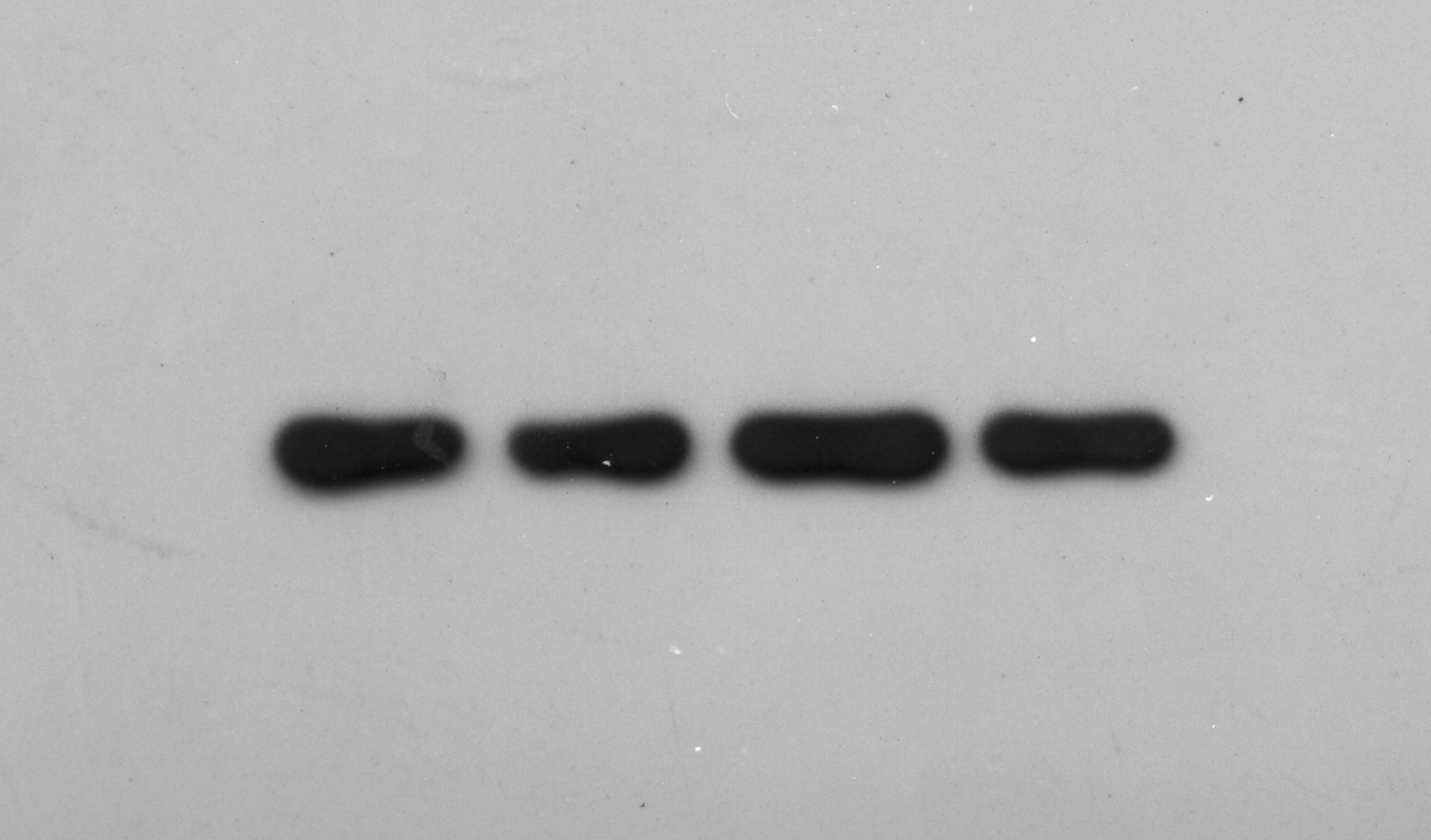

Supplement: Supplementary file 3 — Dataset 2 [file 41417_2024_729_MOESM3_ESM.zip › Dataset 2/Dataset 2/Figure 2/Figure 2a/u373/U373 tub.tif]

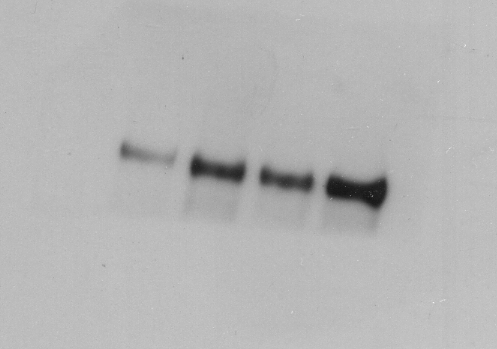

Supplement: Supplementary file 3 — Dataset 2 [file 41417_2024_729_MOESM3_ESM.zip › Dataset 2/Dataset 2/Figure 2/Figure 2b/rcc4/rcc4 IRS1.tif]

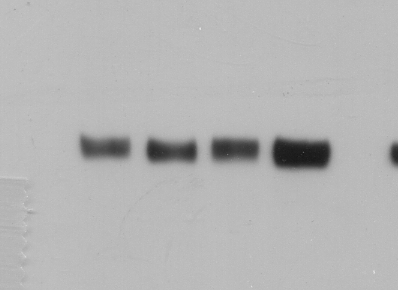

Supplement: Supplementary file 3 — Dataset 2 [file 41417_2024_729_MOESM3_ESM.zip › Dataset 2/Dataset 2/Figure 2/Figure 2b/rcc4/RCC4 IRS2.tif]

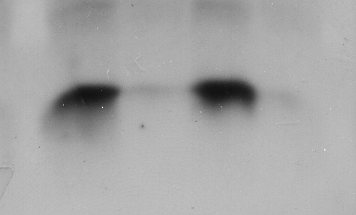

Supplement: Supplementary file 3 — Dataset 2 [file 41417_2024_729_MOESM3_ESM.zip › Dataset 2/Dataset 2/Figure 2/Figure 2b/rcc4/rcc4 N8.tif]

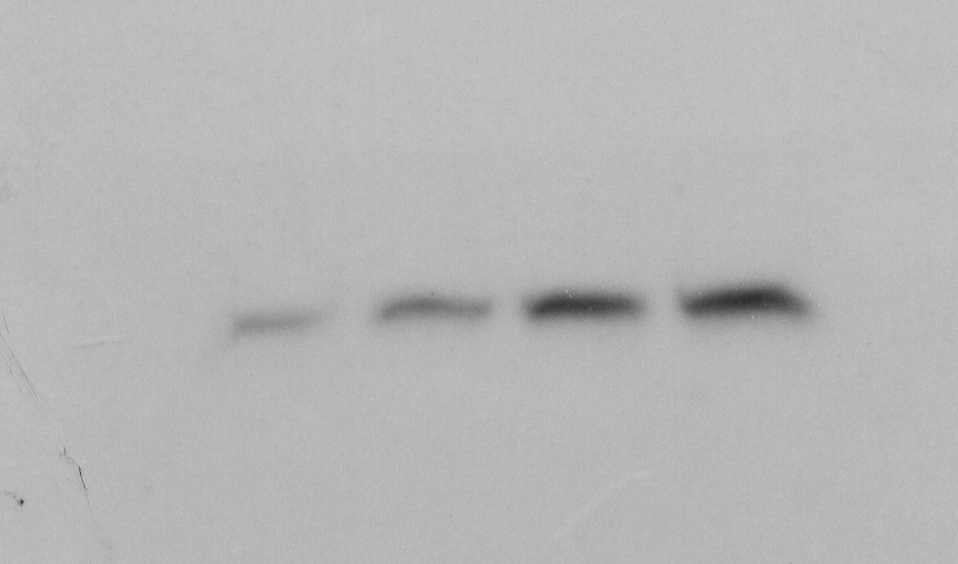

Supplement: Supplementary file 3 — Dataset 2 [file 41417_2024_729_MOESM3_ESM.zip › Dataset 2/Dataset 2/Figure 2/Figure 2b/rcc4/rcc4 pakt.tif]

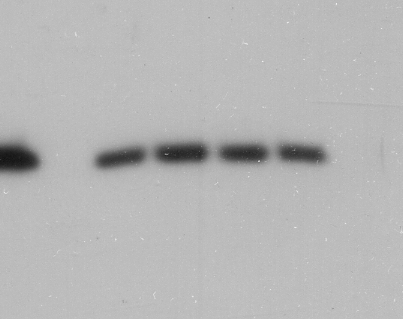

Supplement: Supplementary file 3 — Dataset 2 [file 41417_2024_729_MOESM3_ESM.zip › Dataset 2/Dataset 2/Figure 2/Figure 2b/rcc4/rcc4 tub.tif]

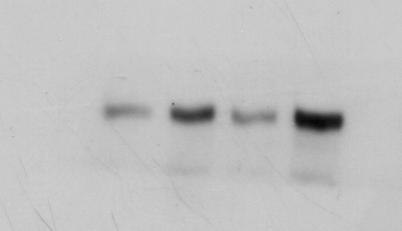

Supplement: Supplementary file 3 — Dataset 2 [file 41417_2024_729_MOESM3_ESM.zip › Dataset 2/Dataset 2/Figure 2/Figure 2b/skov3/SKOV3 IRS1.tif]

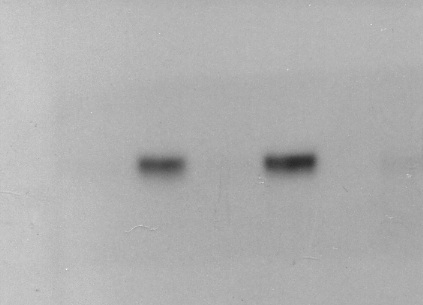

Supplement: Supplementary file 3 — Dataset 2 [file 41417_2024_729_MOESM3_ESM.zip › Dataset 2/Dataset 2/Figure 2/Figure 2b/skov3/SKOV3 IRS2.tif]

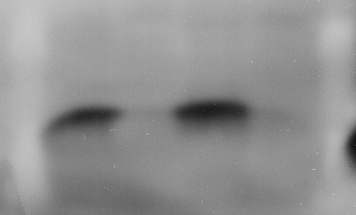

Supplement: Supplementary file 3 — Dataset 2 [file 41417_2024_729_MOESM3_ESM.zip › Dataset 2/Dataset 2/Figure 2/Figure 2b/skov3/SKOV3 N8.tif]

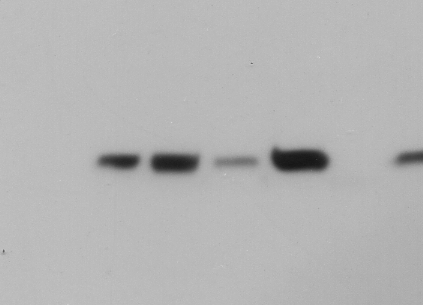

Supplement: Supplementary file 3 — Dataset 2 [file 41417_2024_729_MOESM3_ESM.zip › Dataset 2/Dataset 2/Figure 2/Figure 2b/skov3/SKOV3 pAKT.tif]

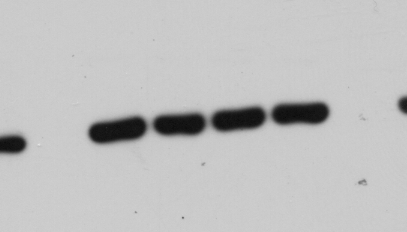

Supplement: Supplementary file 3 — Dataset 2 [file 41417_2024_729_MOESM3_ESM.zip › Dataset 2/Dataset 2/Figure 2/Figure 2b/skov3/SKOV3 Tub.tif]

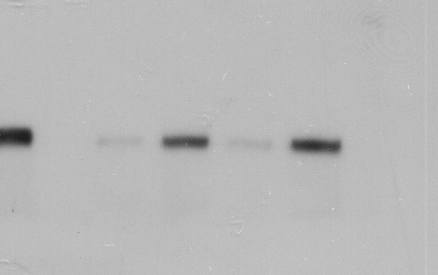

Supplement: Supplementary file 3 — Dataset 2 [file 41417_2024_729_MOESM3_ESM.zip › Dataset 2/Dataset 2/Figure 2/Figure 2b/u373/U373 IRS1.tif]

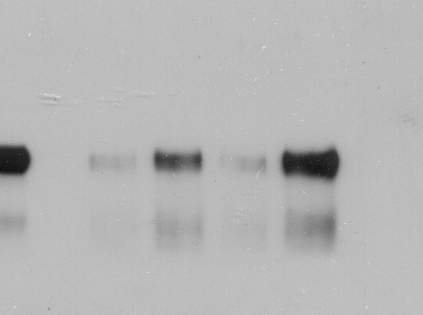

Supplement: Supplementary file 3 — Dataset 2 [file 41417_2024_729_MOESM3_ESM.zip › Dataset 2/Dataset 2/Figure 2/Figure 2b/u373/U373 IRS2.tif]

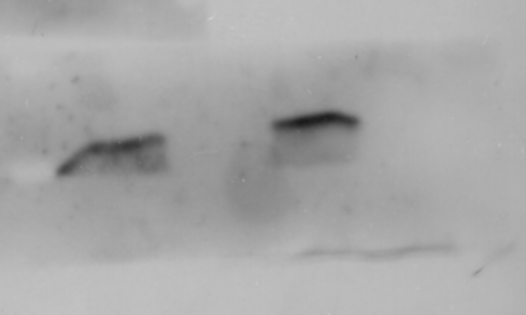

Supplement: Supplementary file 3 — Dataset 2 [file 41417_2024_729_MOESM3_ESM.zip › Dataset 2/Dataset 2/Figure 2/Figure 2b/u373/U373 N8.tif]

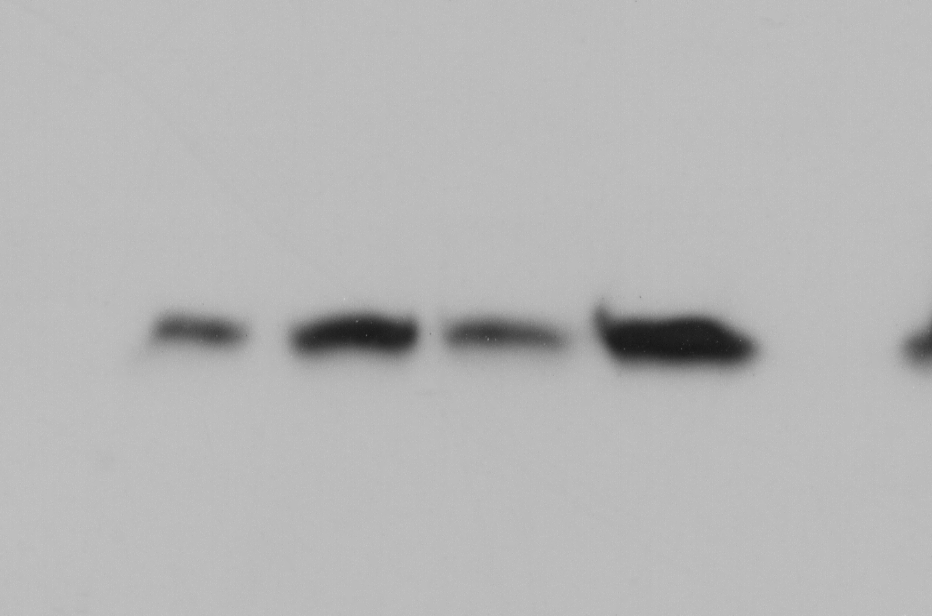

Supplement: Supplementary file 3 — Dataset 2 [file 41417_2024_729_MOESM3_ESM.zip › Dataset 2/Dataset 2/Figure 2/Figure 2b/u373/U373 pakt.tif]

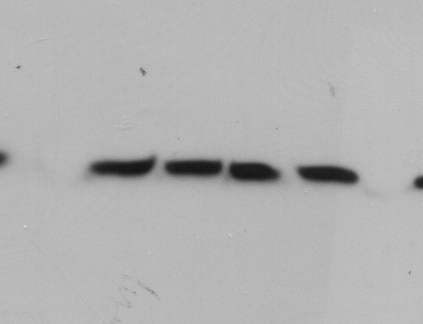

Supplement: Supplementary file 3 — Dataset 2 [file 41417_2024_729_MOESM3_ESM.zip › Dataset 2/Dataset 2/Figure 2/Figure 2b/u373/U373 tub.tif]

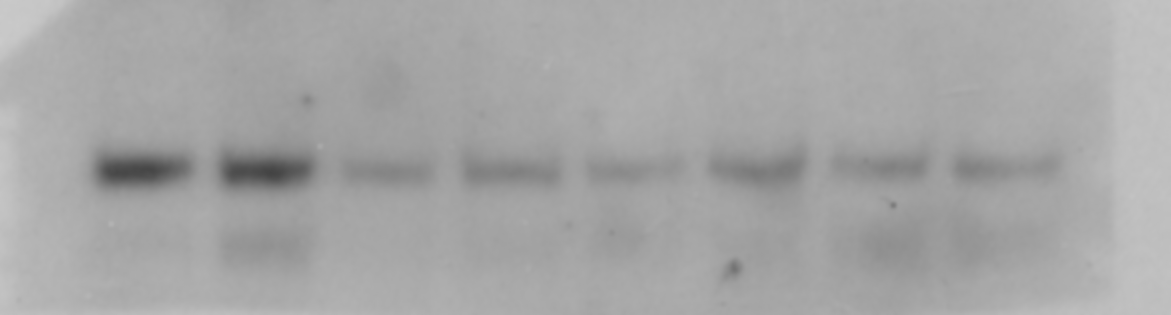

Supplement: Supplementary file 3 — Dataset 2 [file 41417_2024_729_MOESM3_ESM.zip › Dataset 2/Dataset 2/Figure 2/Figure 2c/rcc4 siirs1siirs2mlnins western/rcc4 IRS1.tif]

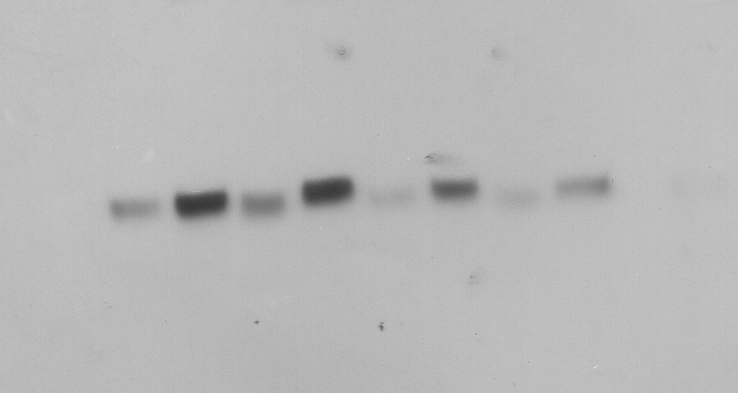

Supplement: Supplementary file 3 — Dataset 2 [file 41417_2024_729_MOESM3_ESM.zip › Dataset 2/Dataset 2/Figure 2/Figure 2c/rcc4 siirs1siirs2mlnins western/rcc4 IRS2.tif]

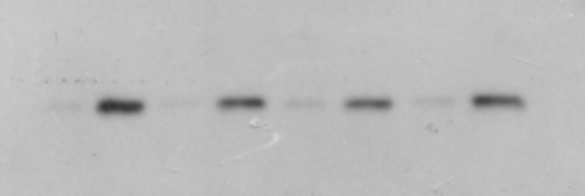

Supplement: Supplementary file 3 — Dataset 2 [file 41417_2024_729_MOESM3_ESM.zip › Dataset 2/Dataset 2/Figure 2/Figure 2c/rcc4 siirs1siirs2mlnins western/rcc4 pakt.tif]

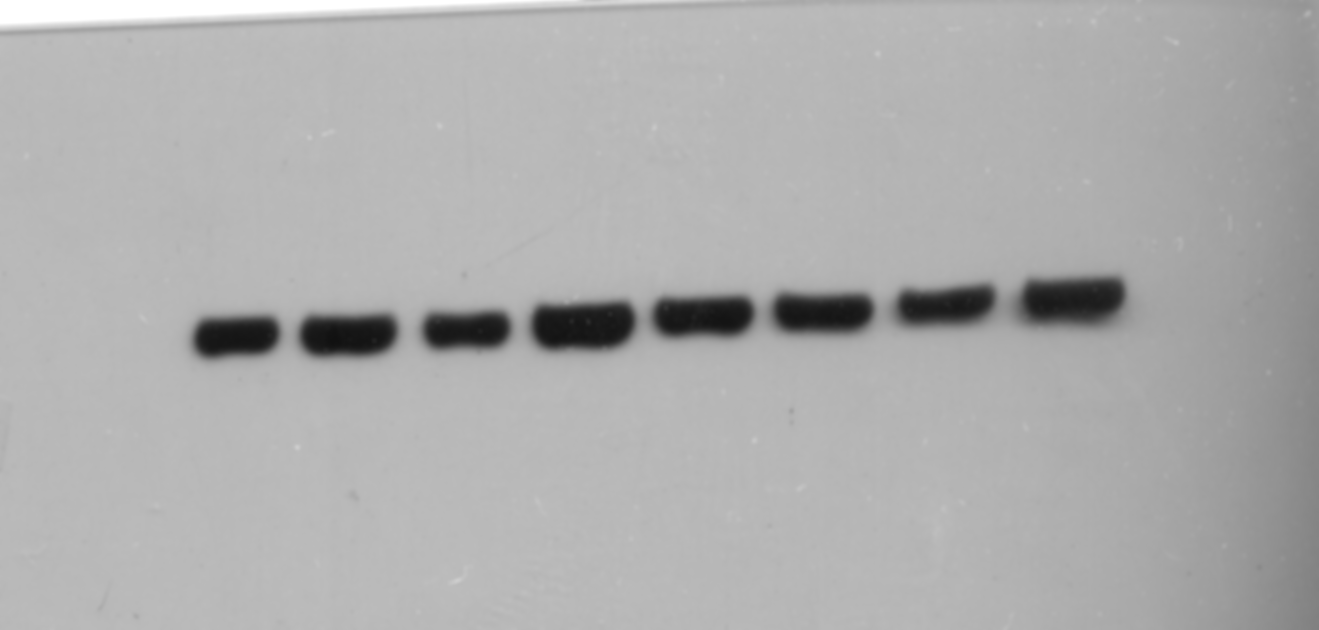

Supplement: Supplementary file 3 — Dataset 2 [file 41417_2024_729_MOESM3_ESM.zip › Dataset 2/Dataset 2/Figure 2/Figure 2c/rcc4 siirs1siirs2mlnins western/rcc4 tub.tif]

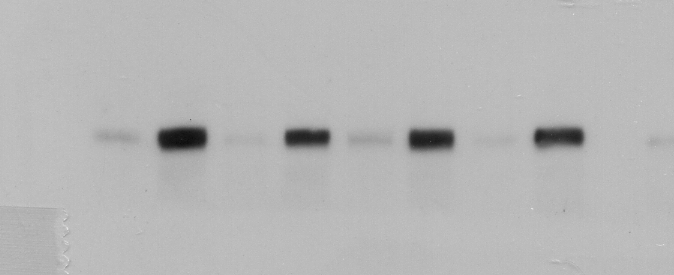

Supplement: Supplementary file 3 — Dataset 2 [file 41417_2024_729_MOESM3_ESM.zip › Dataset 2/Dataset 2/Figure 2/Figure 2c/skov3 siirs1siirs2mlnins western/SKOV3 IRS1.tif]

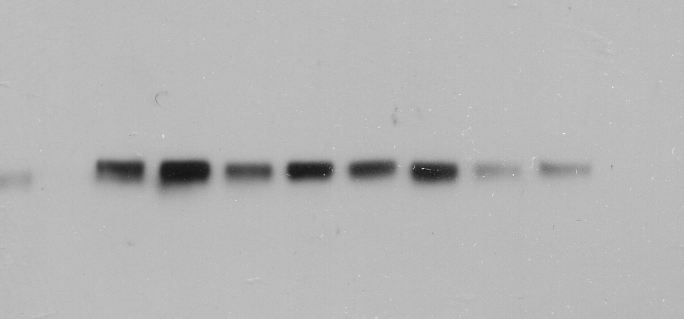

Supplement: Supplementary file 3 — Dataset 2 [file 41417_2024_729_MOESM3_ESM.zip › Dataset 2/Dataset 2/Figure 2/Figure 2c/skov3 siirs1siirs2mlnins western/SKOV3 IRS2.tif]

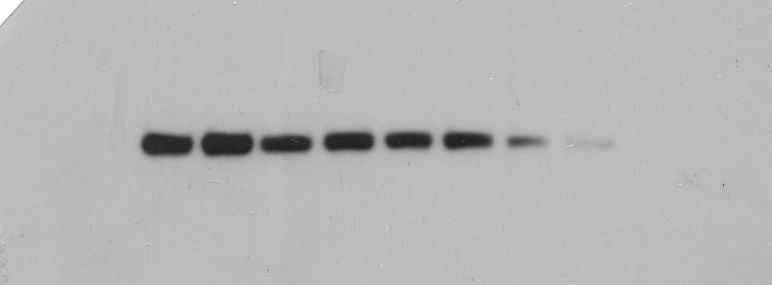

Supplement: Supplementary file 3 — Dataset 2 [file 41417_2024_729_MOESM3_ESM.zip › Dataset 2/Dataset 2/Figure 2/Figure 2c/skov3 siirs1siirs2mlnins western/SKOV3 pAKT.tif]

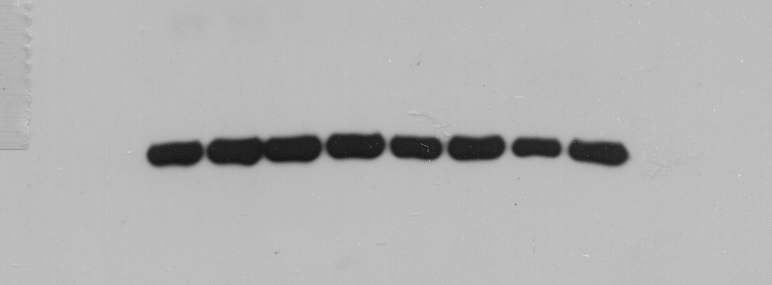

Supplement: Supplementary file 3 — Dataset 2 [file 41417_2024_729_MOESM3_ESM.zip › Dataset 2/Dataset 2/Figure 2/Figure 2c/skov3 siirs1siirs2mlnins western/SKOV3 tub.tif]

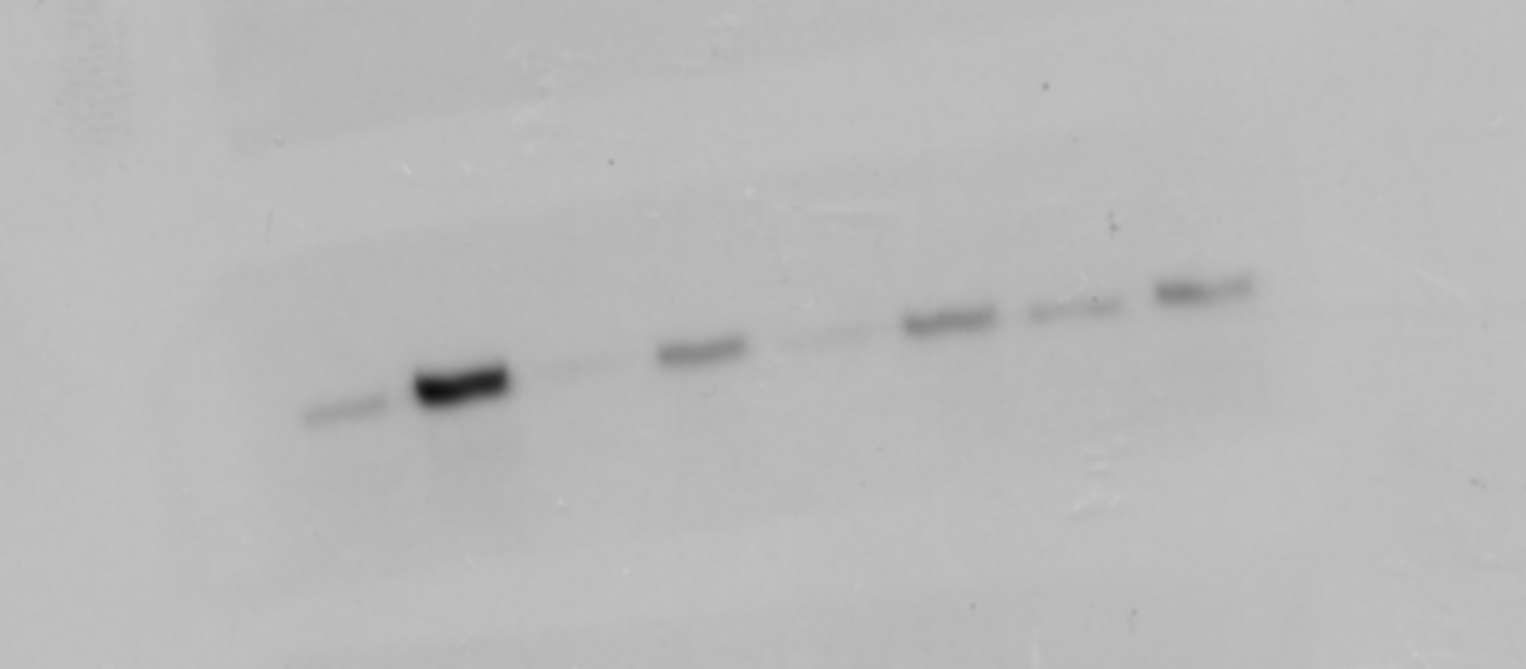

Supplement: Supplementary file 3 — Dataset 2 [file 41417_2024_729_MOESM3_ESM.zip › Dataset 2/Dataset 2/Figure 2/Figure 2c/u373 siirs1siirs2mlnins western/U373 IRS1.tif]

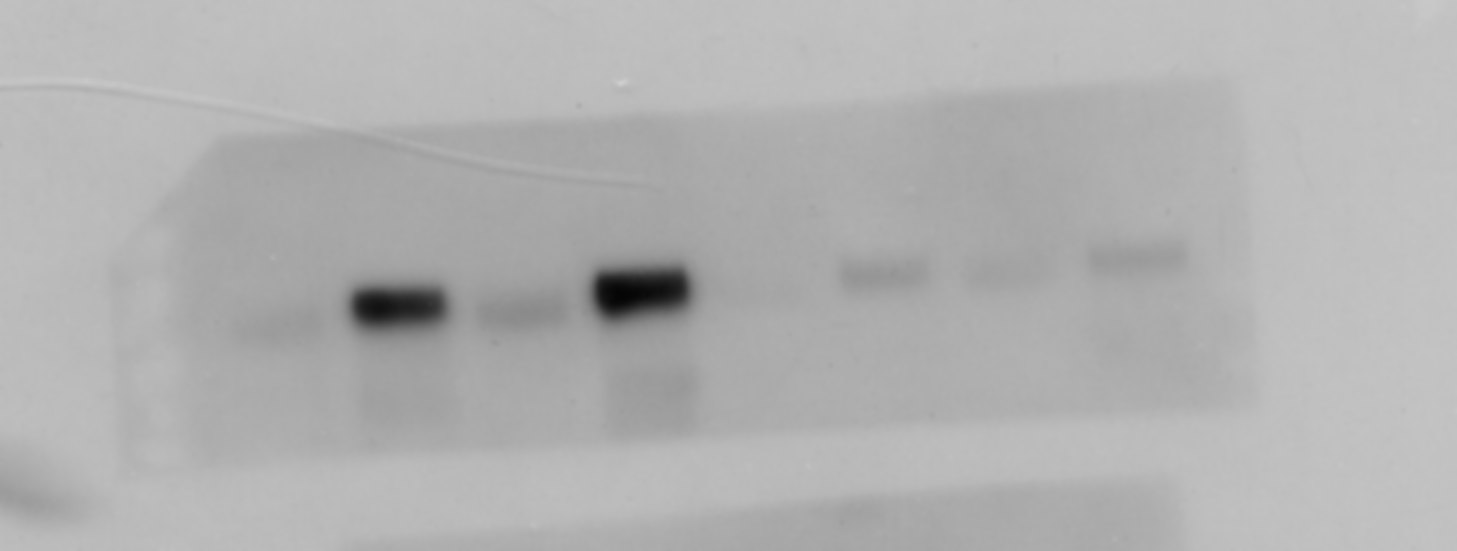

Supplement: Supplementary file 3 — Dataset 2 [file 41417_2024_729_MOESM3_ESM.zip › Dataset 2/Dataset 2/Figure 2/Figure 2c/u373 siirs1siirs2mlnins western/U373 IRS2.tif]

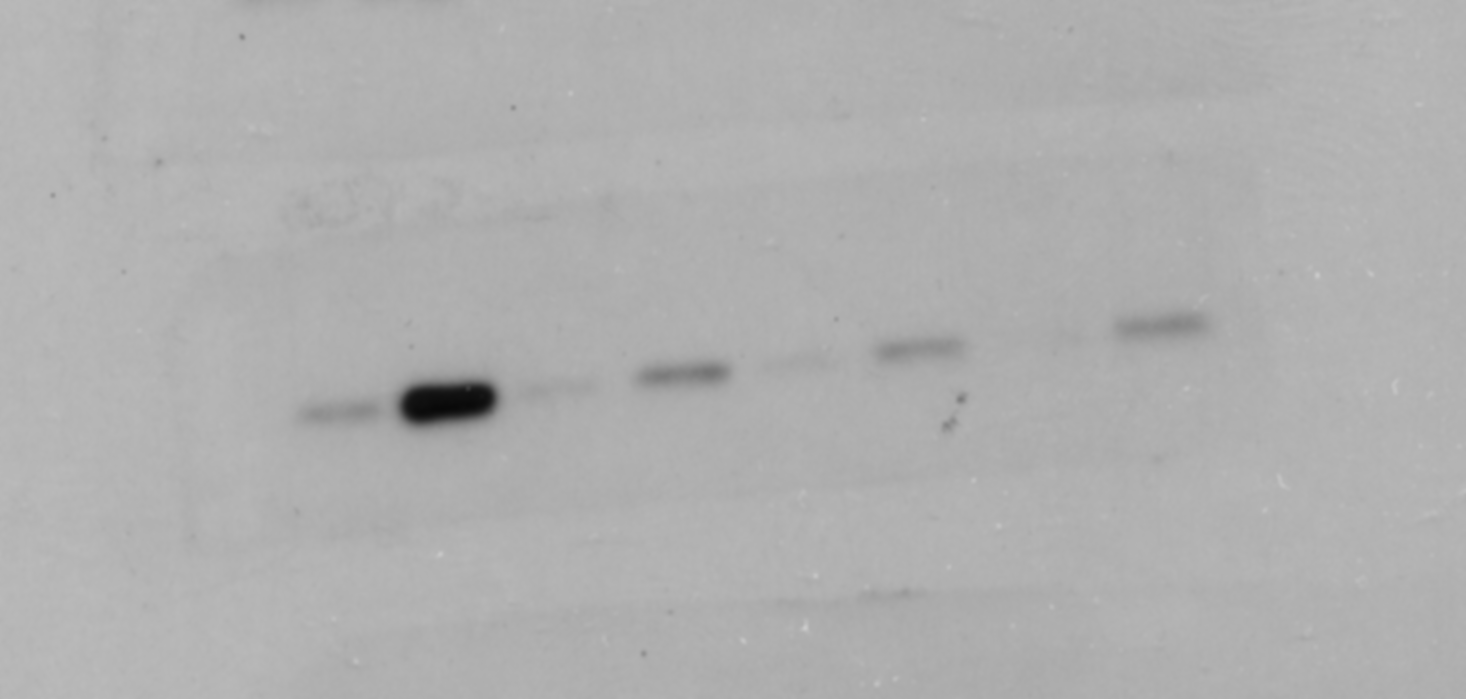

Supplement: Supplementary file 3 — Dataset 2 [file 41417_2024_729_MOESM3_ESM.zip › Dataset 2/Dataset 2/Figure 2/Figure 2c/u373 siirs1siirs2mlnins western/U373 pakt.tif]

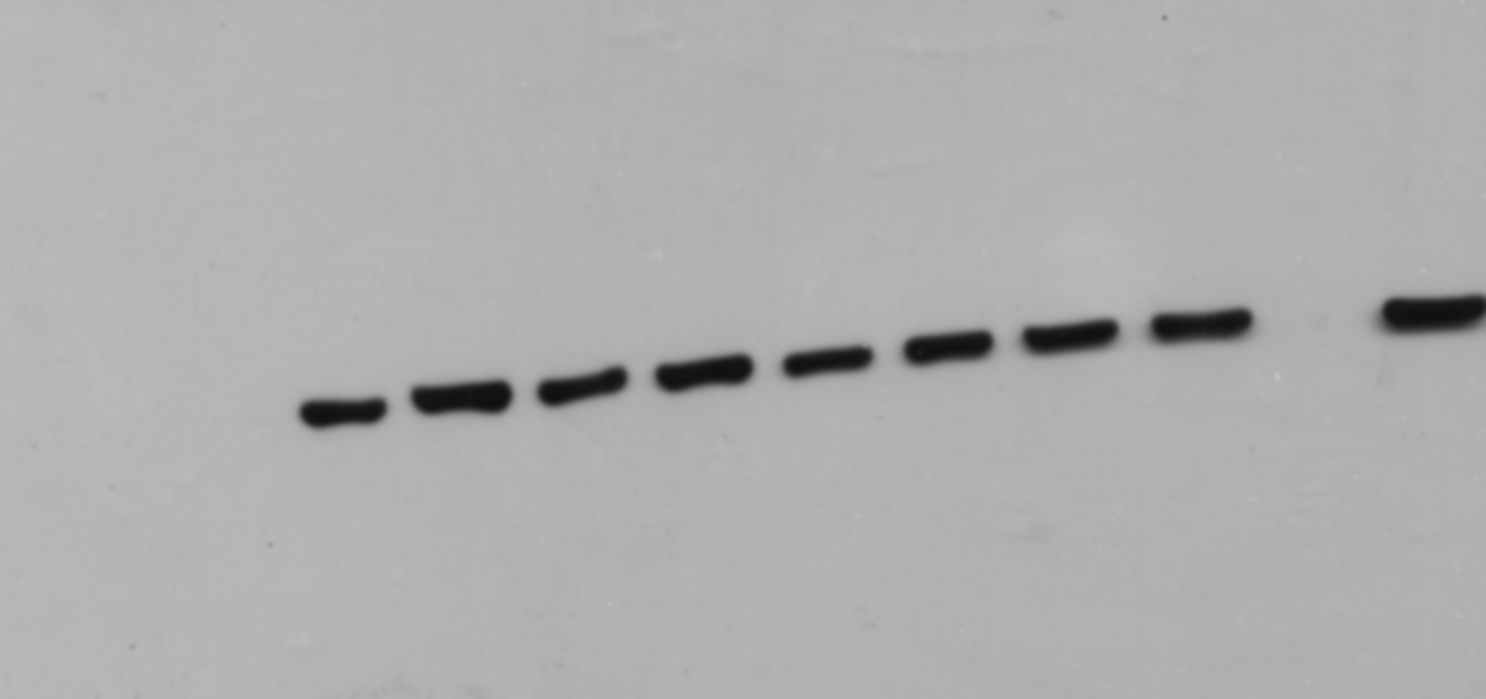

Supplement: Supplementary file 3 — Dataset 2 [file 41417_2024_729_MOESM3_ESM.zip › Dataset 2/Dataset 2/Figure 2/Figure 2c/u373 siirs1siirs2mlnins western/U373 tub.tif]

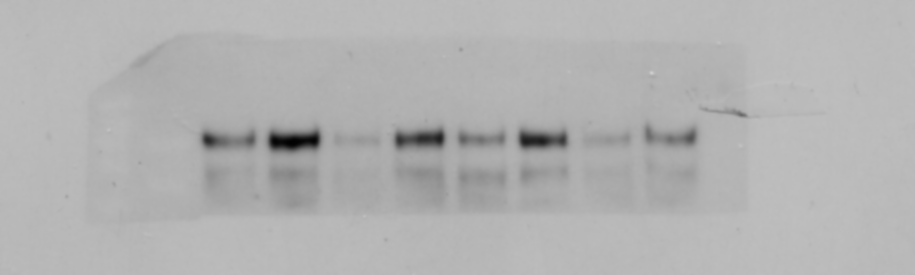

Supplement: Supplementary file 3 — Dataset 2 [file 41417_2024_729_MOESM3_ESM.zip › Dataset 2/Dataset 2/Figure 2/Figure 2d/rcc4 sin8siirs1siirs2ins/rcc4 IRS1.tif]

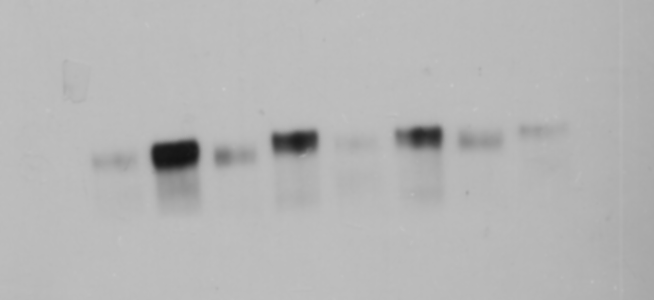

Supplement: Supplementary file 3 — Dataset 2 [file 41417_2024_729_MOESM3_ESM.zip › Dataset 2/Dataset 2/Figure 2/Figure 2d/rcc4 sin8siirs1siirs2ins/rcc4 IRS2.tif]

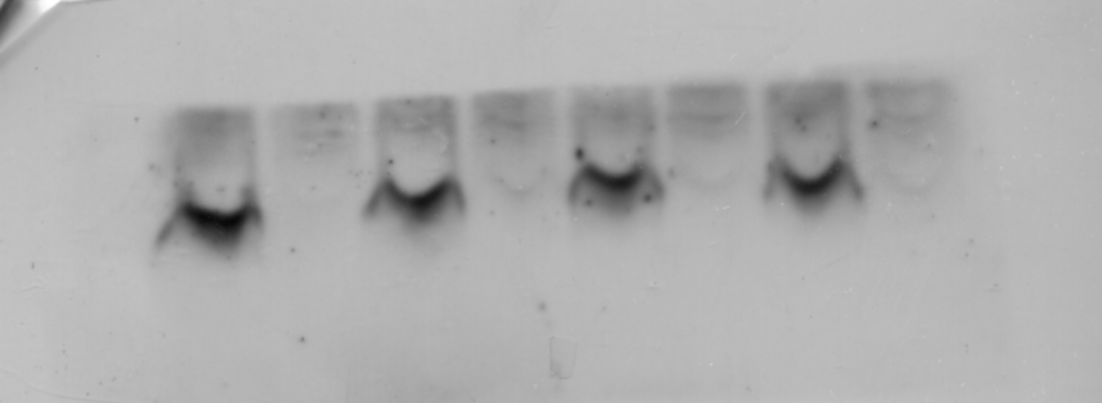

Supplement: Supplementary file 3 — Dataset 2 [file 41417_2024_729_MOESM3_ESM.zip › Dataset 2/Dataset 2/Figure 2/Figure 2d/rcc4 sin8siirs1siirs2ins/rcc4 n8.tif]

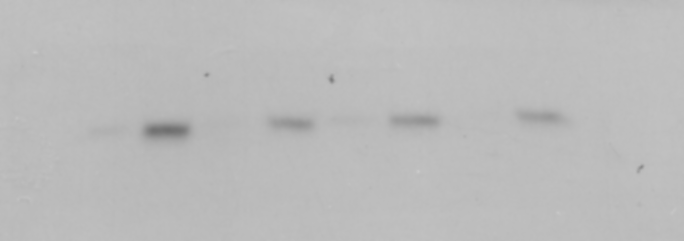

Supplement: Supplementary file 3 — Dataset 2 [file 41417_2024_729_MOESM3_ESM.zip › Dataset 2/Dataset 2/Figure 2/Figure 2d/rcc4 sin8siirs1siirs2ins/rcc4 pakt.tif]

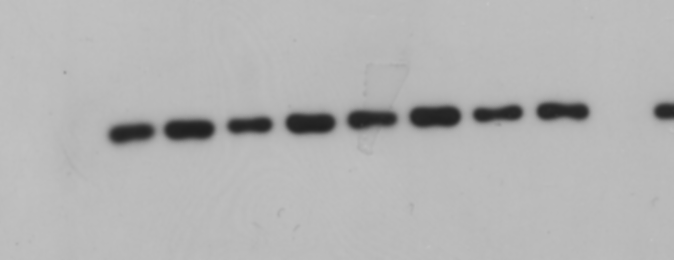

Supplement: Supplementary file 3 — Dataset 2 [file 41417_2024_729_MOESM3_ESM.zip › Dataset 2/Dataset 2/Figure 2/Figure 2d/rcc4 sin8siirs1siirs2ins/rcc4 tub.tif]

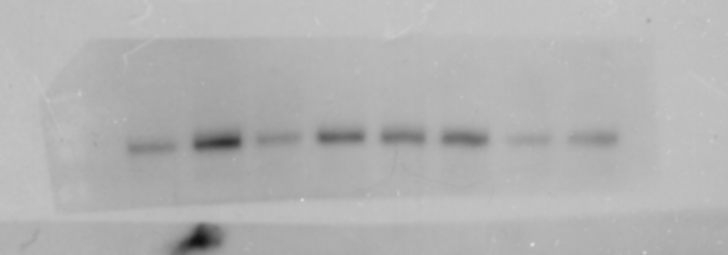

Supplement: Supplementary file 3 — Dataset 2 [file 41417_2024_729_MOESM3_ESM.zip › Dataset 2/Dataset 2/Figure 2/Figure 2d/skov3 sin8siirs1siirs2ins/SKOV3 IRS1.tif]

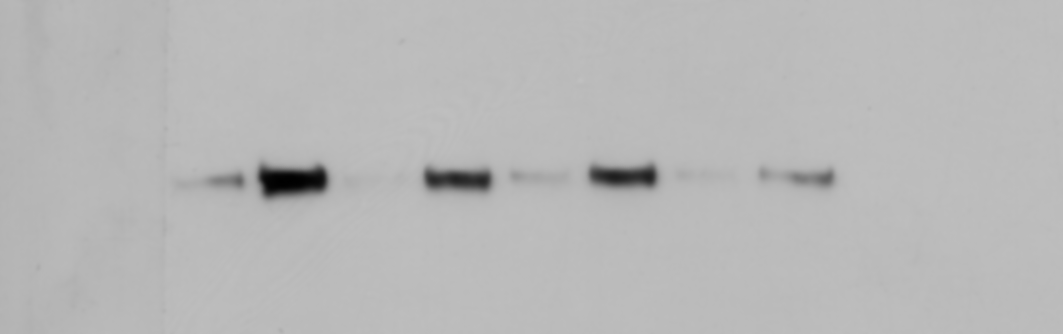

Supplement: Supplementary file 3 — Dataset 2 [file 41417_2024_729_MOESM3_ESM.zip › Dataset 2/Dataset 2/Figure 2/Figure 2d/skov3 sin8siirs1siirs2ins/SKOV3 irs2.tif]

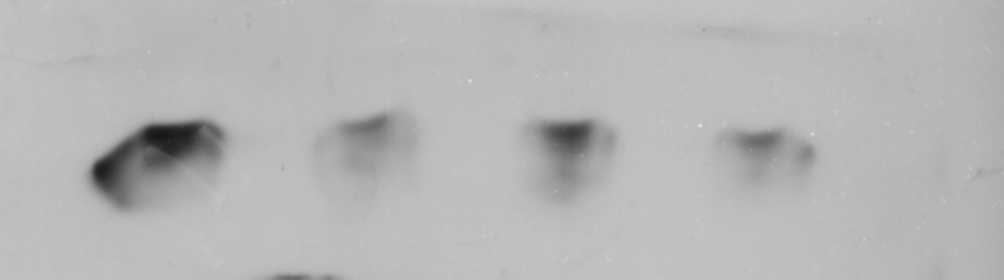

Supplement: Supplementary file 3 — Dataset 2 [file 41417_2024_729_MOESM3_ESM.zip › Dataset 2/Dataset 2/Figure 2/Figure 2d/skov3 sin8siirs1siirs2ins/SKOV3 N8.tif]

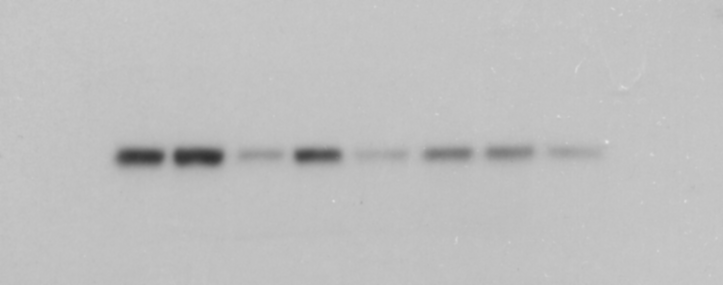

Supplement: Supplementary file 3 — Dataset 2 [file 41417_2024_729_MOESM3_ESM.zip › Dataset 2/Dataset 2/Figure 2/Figure 2d/skov3 sin8siirs1siirs2ins/SKOV3 pAKT.tif]

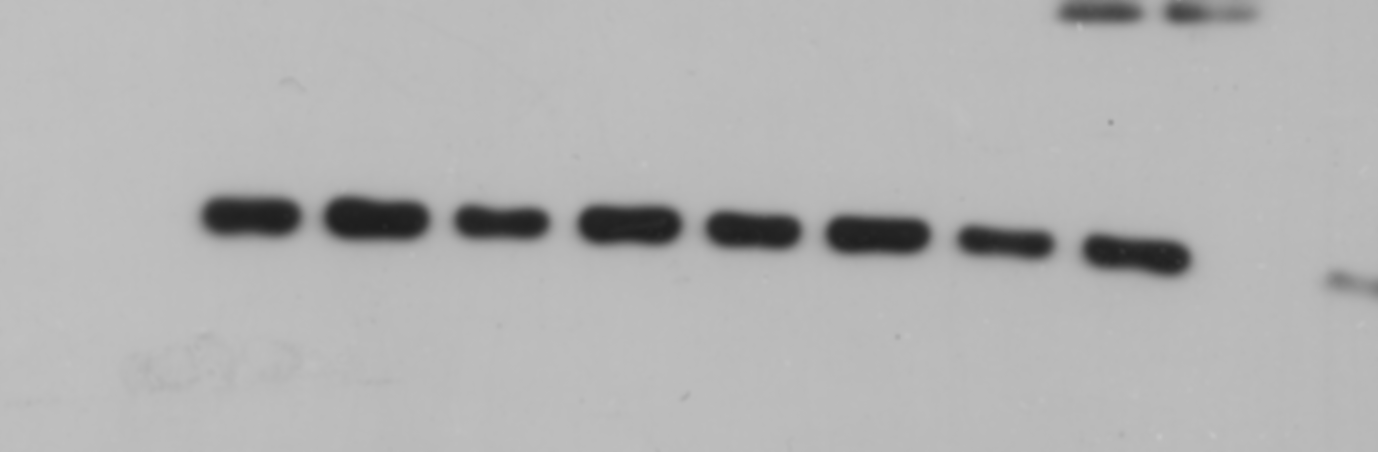

Supplement: Supplementary file 3 — Dataset 2 [file 41417_2024_729_MOESM3_ESM.zip › Dataset 2/Dataset 2/Figure 2/Figure 2d/skov3 sin8siirs1siirs2ins/SKOV3 tub.tif]

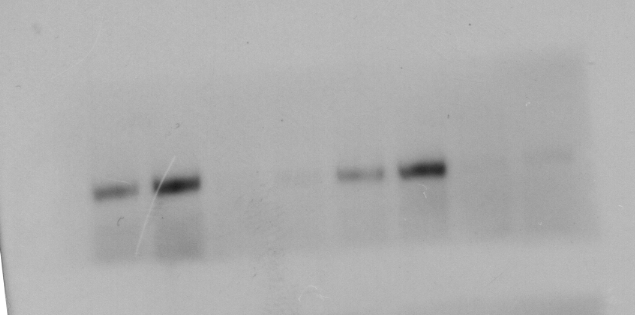

Supplement: Supplementary file 3 — Dataset 2 [file 41417_2024_729_MOESM3_ESM.zip › Dataset 2/Dataset 2/Figure 2/Figure 2d/u373 sin8siirs1siirs2ins/U373 IRS1.tif]

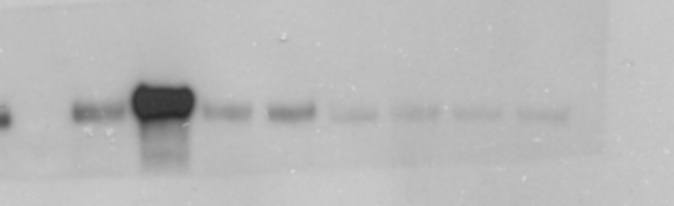

Supplement: Supplementary file 3 — Dataset 2 [file 41417_2024_729_MOESM3_ESM.zip › Dataset 2/Dataset 2/Figure 2/Figure 2d/u373 sin8siirs1siirs2ins/U373 IRS2.tif]

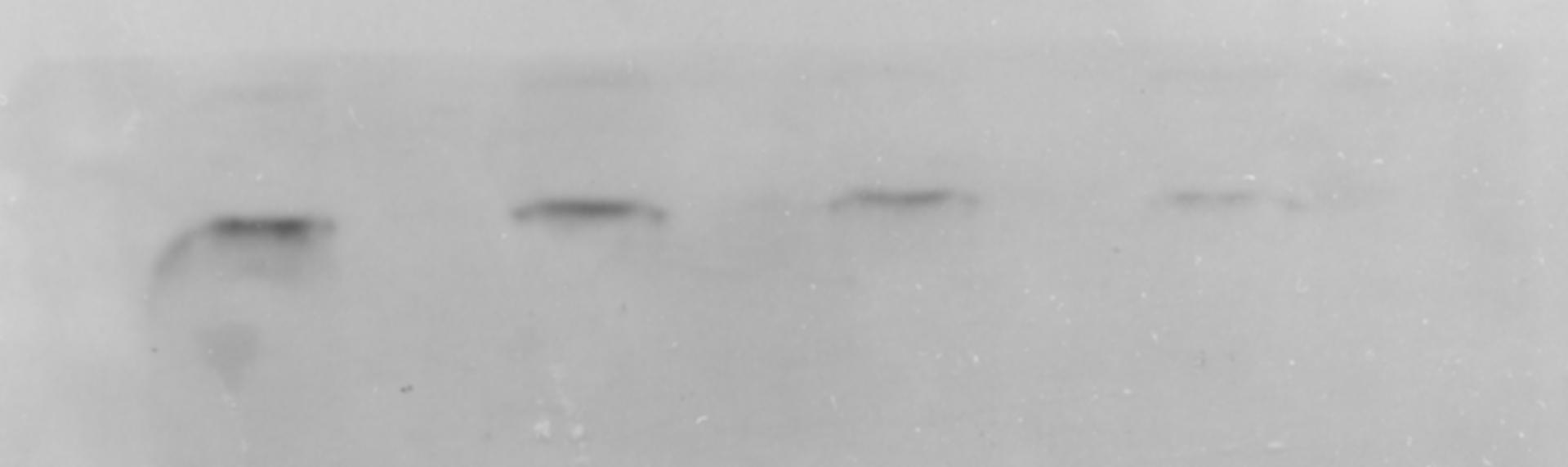

Supplement: Supplementary file 3 — Dataset 2 [file 41417_2024_729_MOESM3_ESM.zip › Dataset 2/Dataset 2/Figure 2/Figure 2d/u373 sin8siirs1siirs2ins/U373 n8.tif]

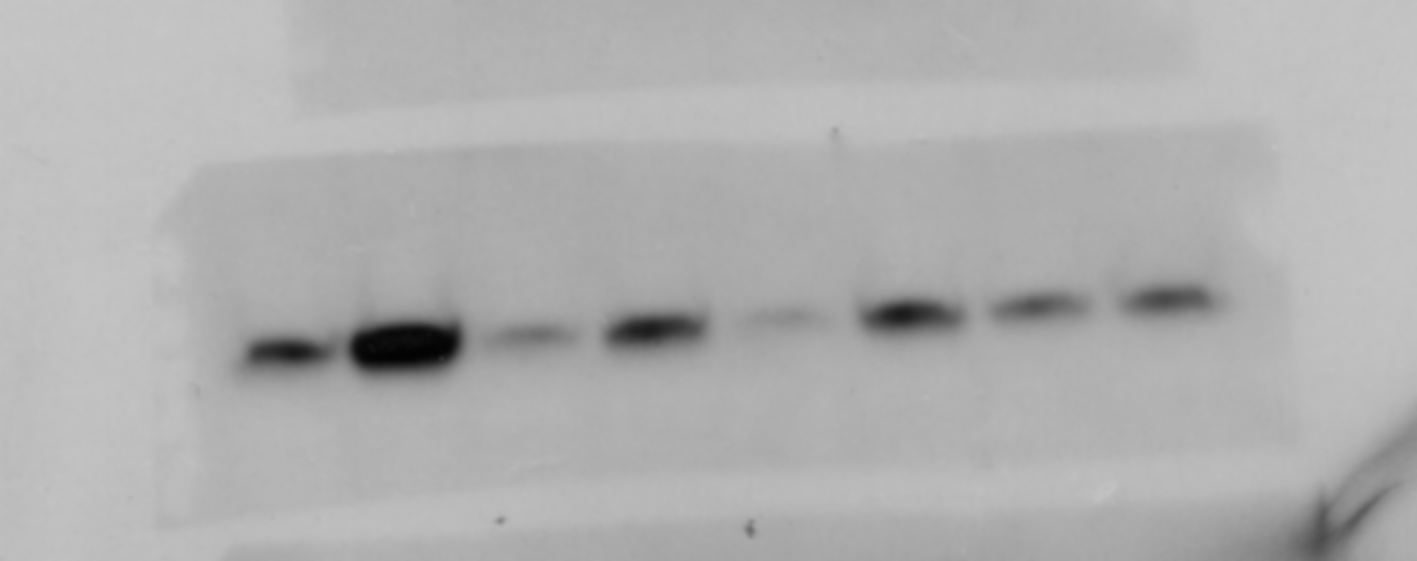

Supplement: Supplementary file 3 — Dataset 2 [file 41417_2024_729_MOESM3_ESM.zip › Dataset 2/Dataset 2/Figure 2/Figure 2d/u373 sin8siirs1siirs2ins/U373 pakt.tif]

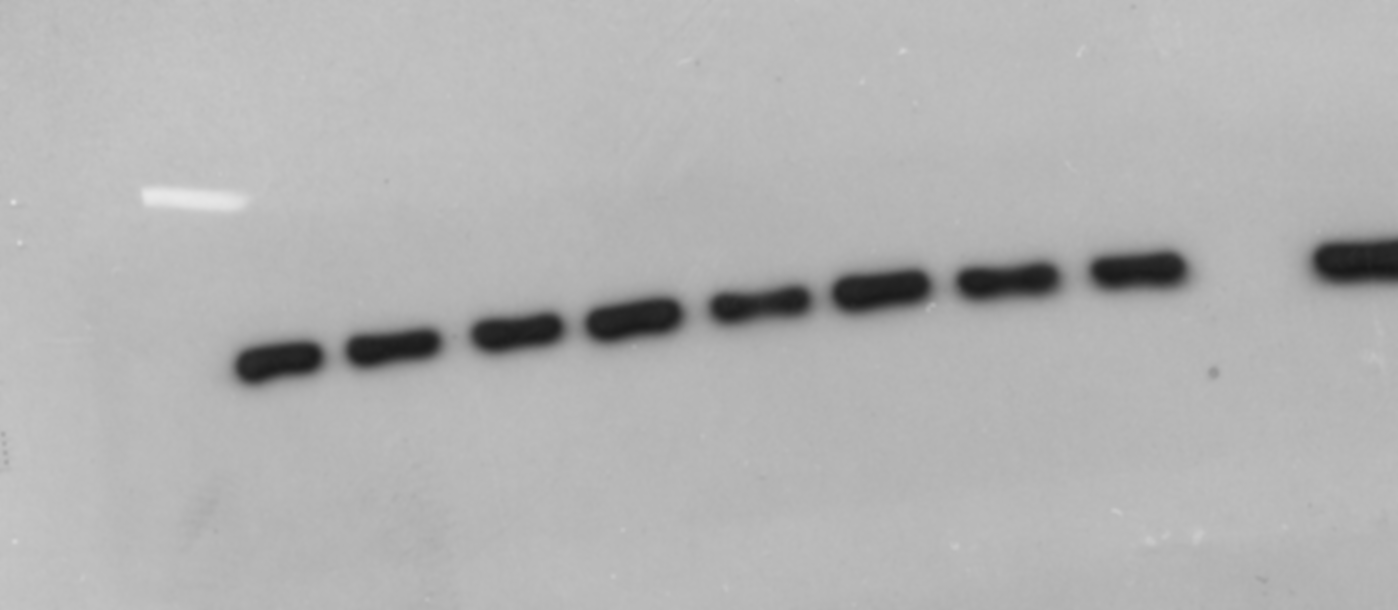

Supplement: Supplementary file 3 — Dataset 2 [file 41417_2024_729_MOESM3_ESM.zip › Dataset 2/Dataset 2/Figure 2/Figure 2d/u373 sin8siirs1siirs2ins/U373 Tub.tif]

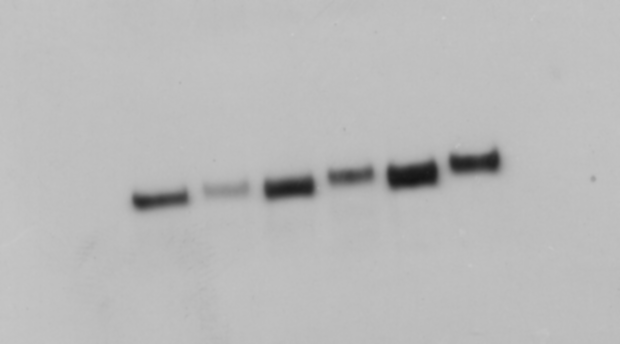

Supplement: Supplementary file 4 — Dataset 3 [file 41417_2024_729_MOESM4_ESM.zip › Figure 3/Figure 3b/RCC4/rcc4 irs1.tif]

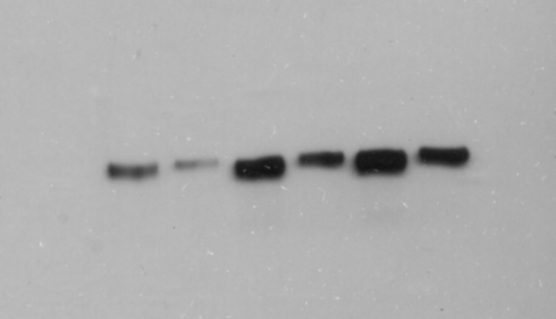

Supplement: Supplementary file 4 — Dataset 3 [file 41417_2024_729_MOESM4_ESM.zip › Figure 3/Figure 3b/RCC4/rcc4 irs2.tif]

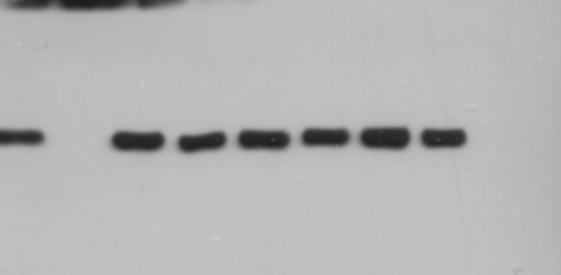

Supplement: Supplementary file 4 — Dataset 3 [file 41417_2024_729_MOESM4_ESM.zip › Figure 3/Figure 3b/RCC4/rcc4 tub.tif]

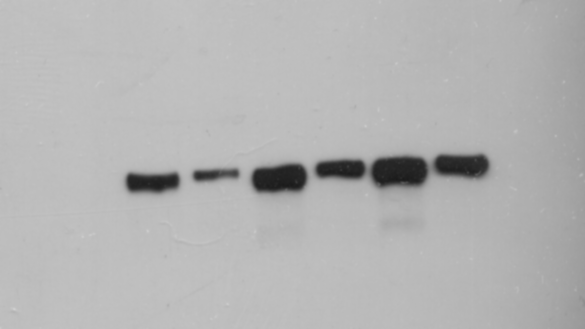

Supplement: Supplementary file 4 — Dataset 3 [file 41417_2024_729_MOESM4_ESM.zip › Figure 3/Figure 3b/SKOV3/SKOV3 IRS1.tif]

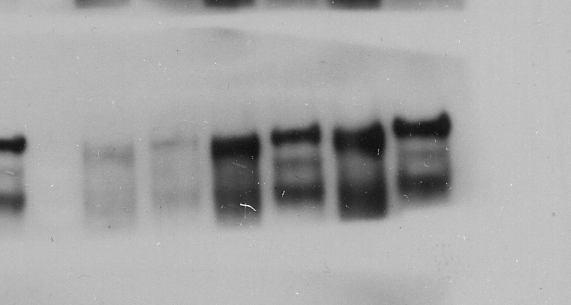

Supplement: Supplementary file 4 — Dataset 3 [file 41417_2024_729_MOESM4_ESM.zip › Figure 3/Figure 3b/SKOV3/SKOV3 IRS2.tif]

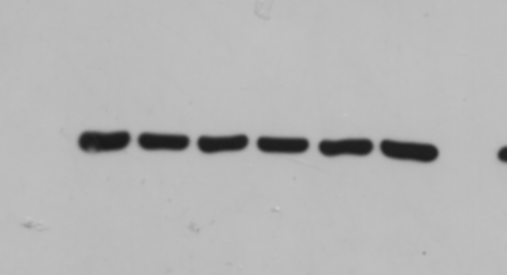

Supplement: Supplementary file 4 — Dataset 3 [file 41417_2024_729_MOESM4_ESM.zip › Figure 3/Figure 3b/SKOV3/SKOV3 tub.tif]

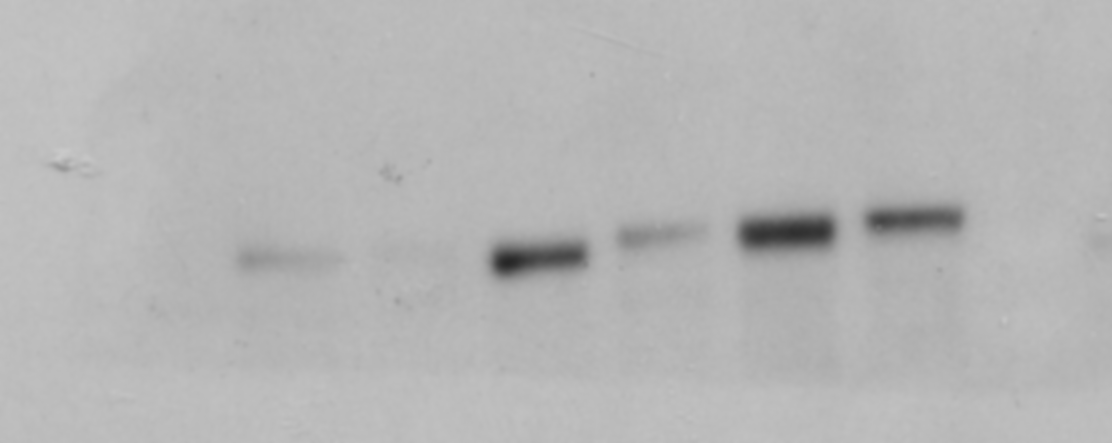

Supplement: Supplementary file 4 — Dataset 3 [file 41417_2024_729_MOESM4_ESM.zip › Figure 3/Figure 3b/U373/U373 IRS1.tif]

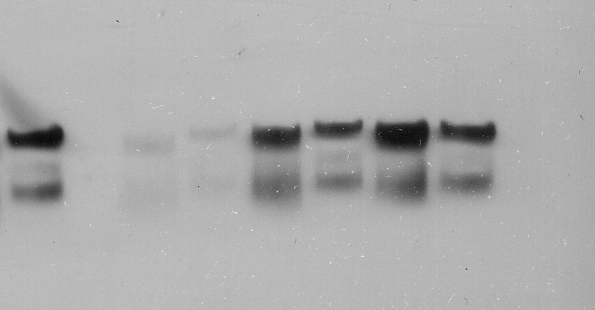

Supplement: Supplementary file 4 — Dataset 3 [file 41417_2024_729_MOESM4_ESM.zip › Figure 3/Figure 3b/U373/U373 IRS2.tif]

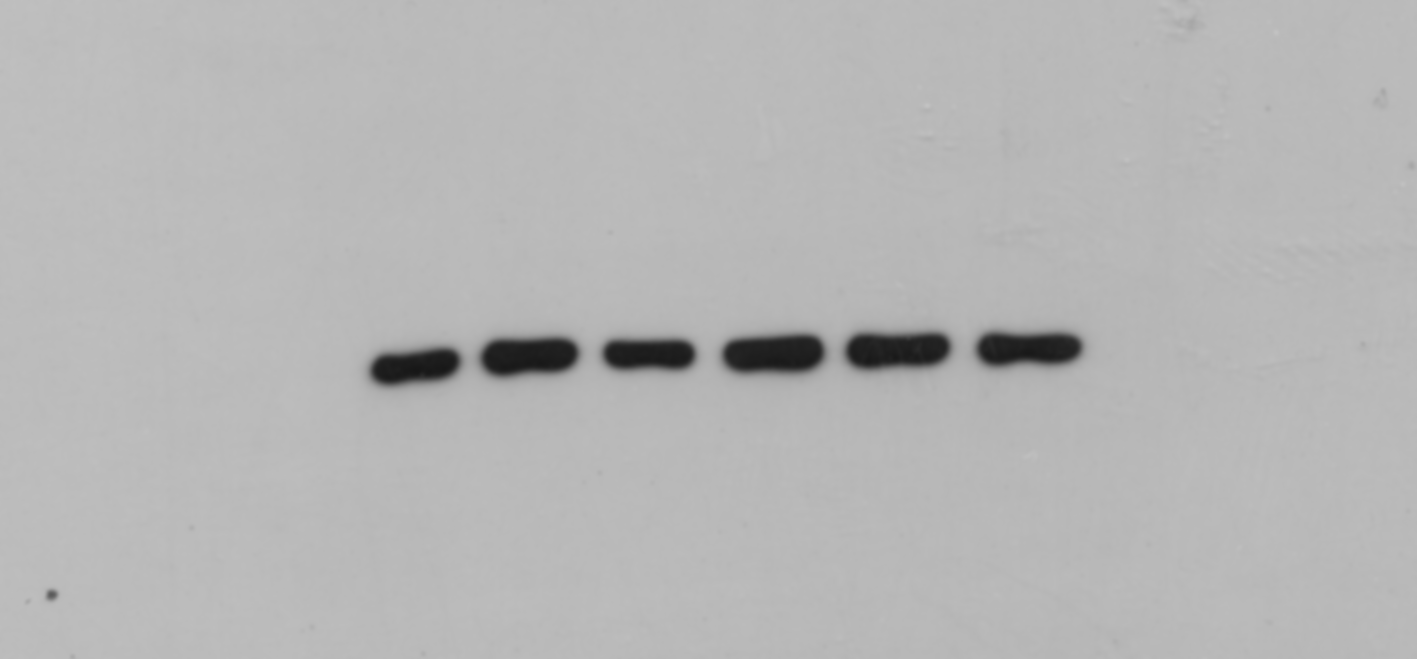

Supplement: Supplementary file 4 — Dataset 3 [file 41417_2024_729_MOESM4_ESM.zip › Figure 3/Figure 3b/U373/U373 Tub.tif]

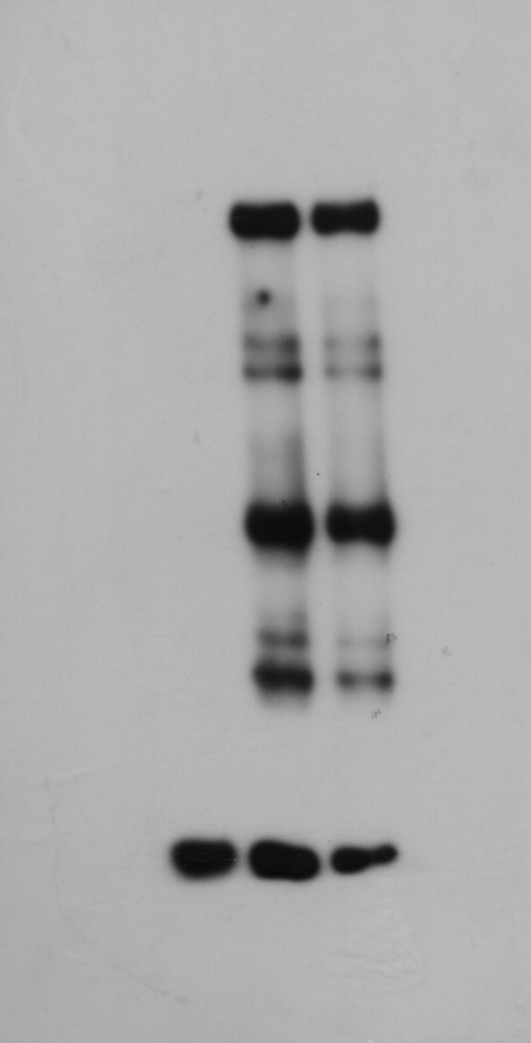

Supplement: Supplementary file 4 — Dataset 3 [file 41417_2024_729_MOESM4_ESM.zip › Figure 3/Figure 3c/flag-irs1 flag.tif]

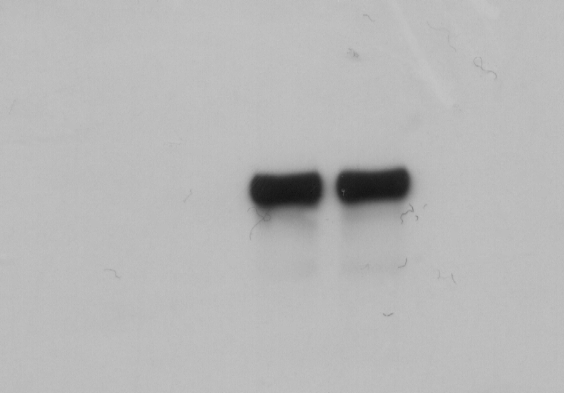

Supplement: Supplementary file 4 — Dataset 3 [file 41417_2024_729_MOESM4_ESM.zip › Figure 3/Figure 3c/flag-irs1 input irs1.tif]

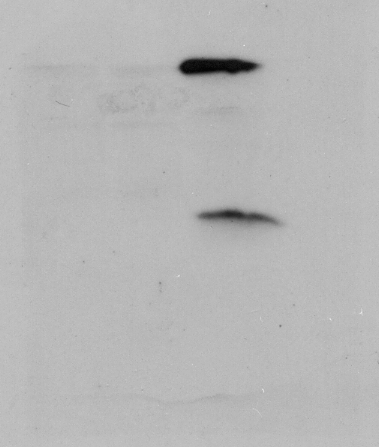

Supplement: Supplementary file 4 — Dataset 3 [file 41417_2024_729_MOESM4_ESM.zip › Figure 3/Figure 3c/flag-irs1 input n8.tif]

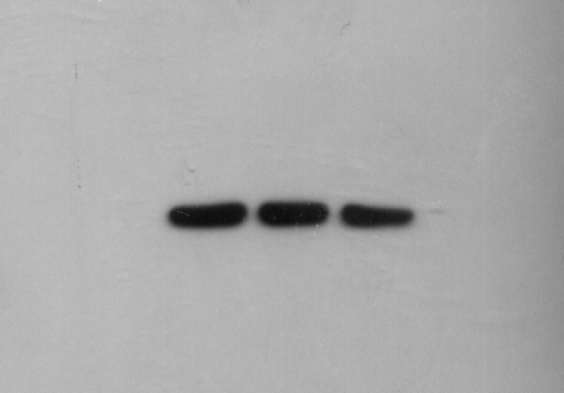

Supplement: Supplementary file 4 — Dataset 3 [file 41417_2024_729_MOESM4_ESM.zip › Figure 3/Figure 3c/flag-irs1 input tub.tif]

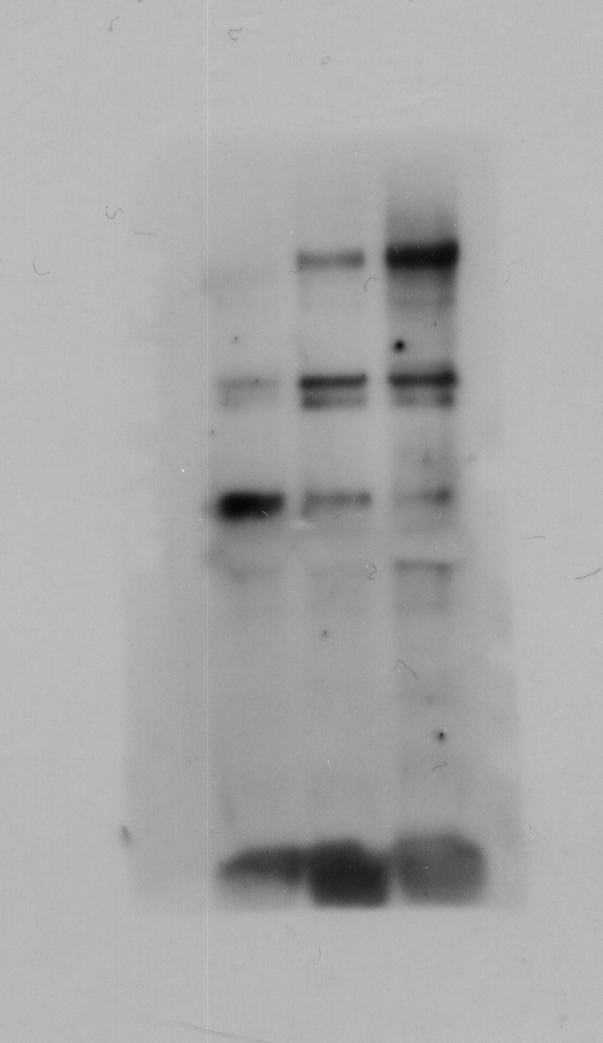

Supplement: Supplementary file 4 — Dataset 3 [file 41417_2024_729_MOESM4_ESM.zip › Figure 3/Figure 3c/flag-irs1 N8.tif]

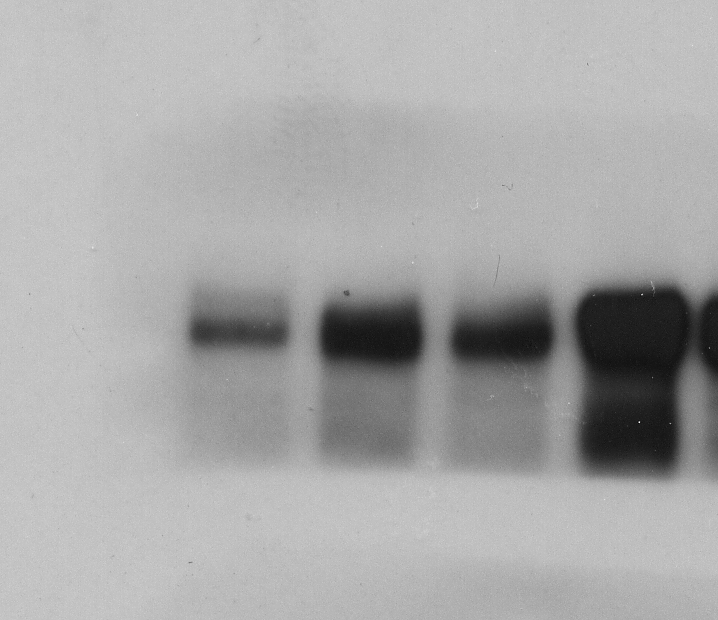

Supplement: Supplementary file 4 — Dataset 3 [file 41417_2024_729_MOESM4_ESM.zip › Figure 3/Figure 3c/myc-irs2 input irs2.tif]

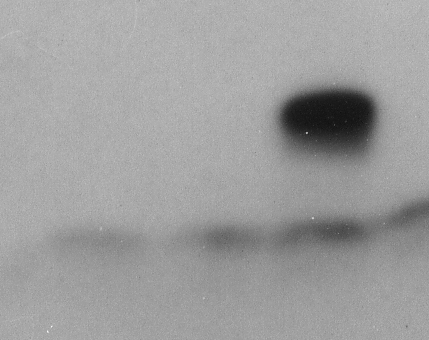

Supplement: Supplementary file 4 — Dataset 3 [file 41417_2024_729_MOESM4_ESM.zip › Figure 3/Figure 3c/myc-irs2 input n8.tif]

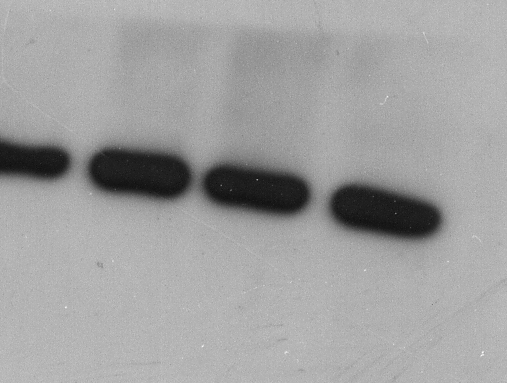

Supplement: Supplementary file 4 — Dataset 3 [file 41417_2024_729_MOESM4_ESM.zip › Figure 3/Figure 3c/myc-irs2 input tub.tif]

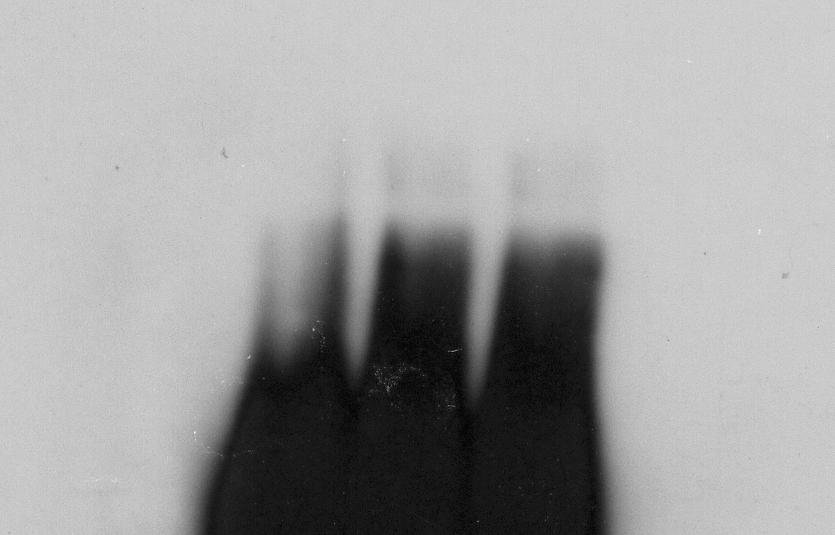

Supplement: Supplementary file 4 — Dataset 3 [file 41417_2024_729_MOESM4_ESM.zip › Figure 3/Figure 3c/myc-irs2 myc.tif]

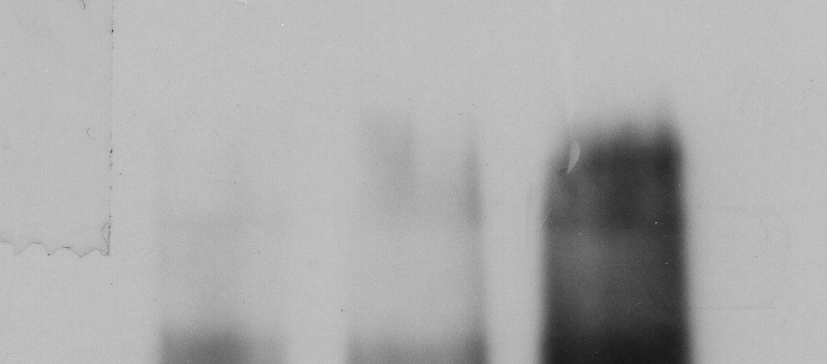

Supplement: Supplementary file 4 — Dataset 3 [file 41417_2024_729_MOESM4_ESM.zip › Figure 3/Figure 3c/myc-irs2 N8.tif]

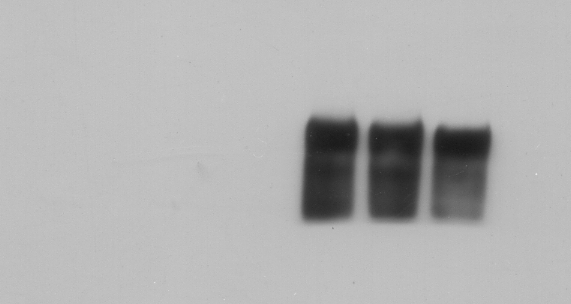

Supplement: Supplementary file 4 — Dataset 3 [file 41417_2024_729_MOESM4_ESM.zip › Figure 3/Figure 3d/flag-irs1 nibinding input irs1.tif]

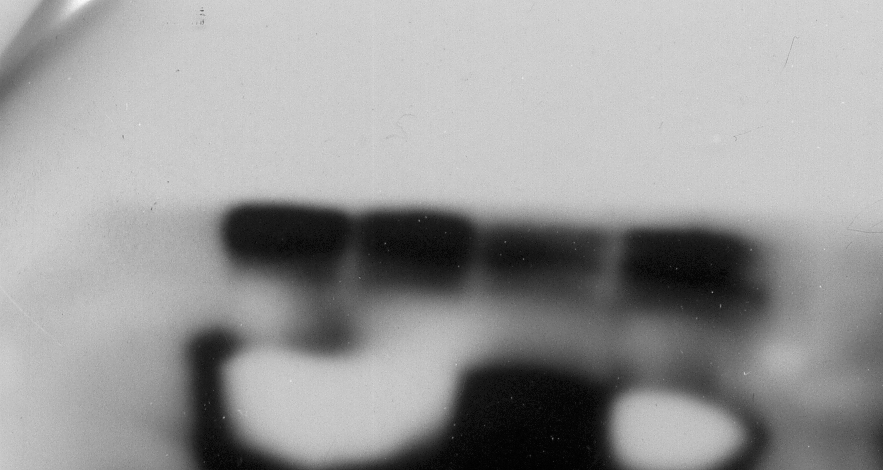

Supplement: Supplementary file 4 — Dataset 3 [file 41417_2024_729_MOESM4_ESM.zip › Figure 3/Figure 3d/flag-irs1 nibinding input n8.tif]

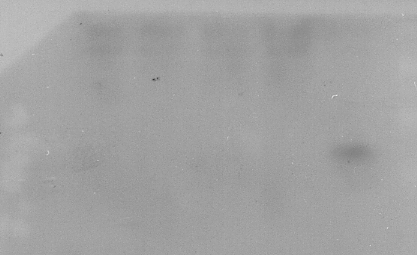

Supplement: Supplementary file 4 — Dataset 3 [file 41417_2024_729_MOESM4_ESM.zip › Figure 3/Figure 3d/flag-irs1 nibinding input senp8.tif]

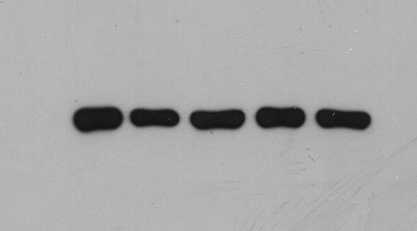

Supplement: Supplementary file 4 — Dataset 3 [file 41417_2024_729_MOESM4_ESM.zip › Figure 3/Figure 3d/flag-irs1 nibinding input tub.tif]

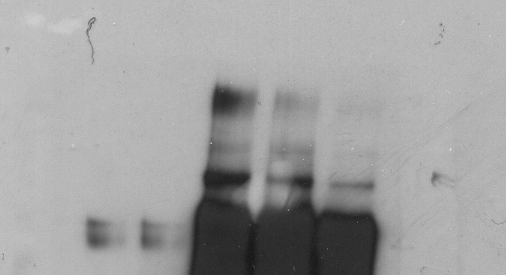

Supplement: Supplementary file 4 — Dataset 3 [file 41417_2024_729_MOESM4_ESM.zip › Figure 3/Figure 3d/flag-irs1 nibinding irs1.tif]

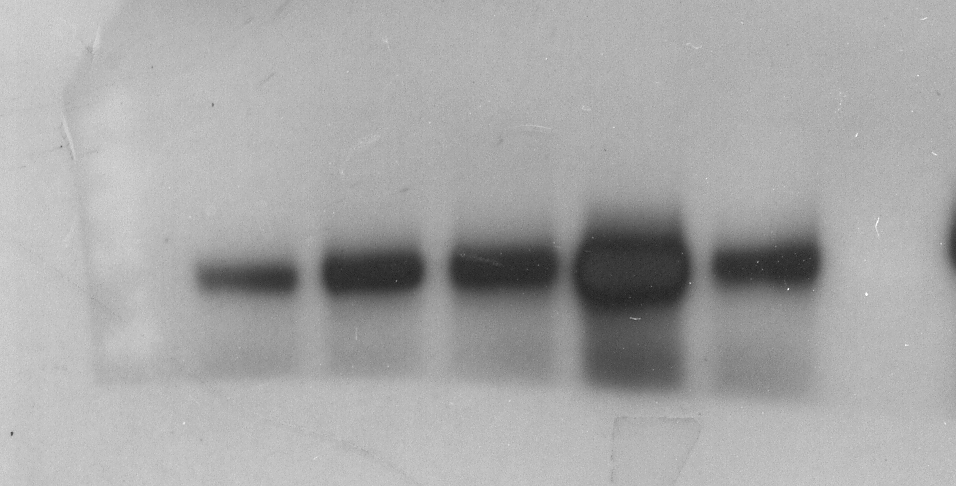

Supplement: Supplementary file 4 — Dataset 3 [file 41417_2024_729_MOESM4_ESM.zip › Figure 3/Figure 3d/myc-irs2 nibinding input irs2.tif]
