# Supplementary figures and images for: Neddylation of insulin receptor substrate acts as a bona fide regulator of insulin signaling and its implications for cancer cell migration (part 2 of 3)
Source: Cancer Gene Ther. 2024 Jan 25;31(4):599–611. doi: 10.1038/s41417-024-00729-z (PMC11016467; doi:10.1038/s41417-024-00729-z)

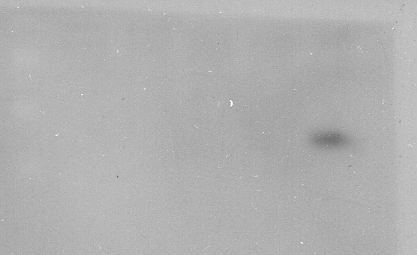

Supplement: Supplementary file 4 — Dataset 3 [file 41417_2024_729_MOESM4_ESM.zip › Figure 3/Figure 3d/myc-irs2 nibinding input myc.tif]

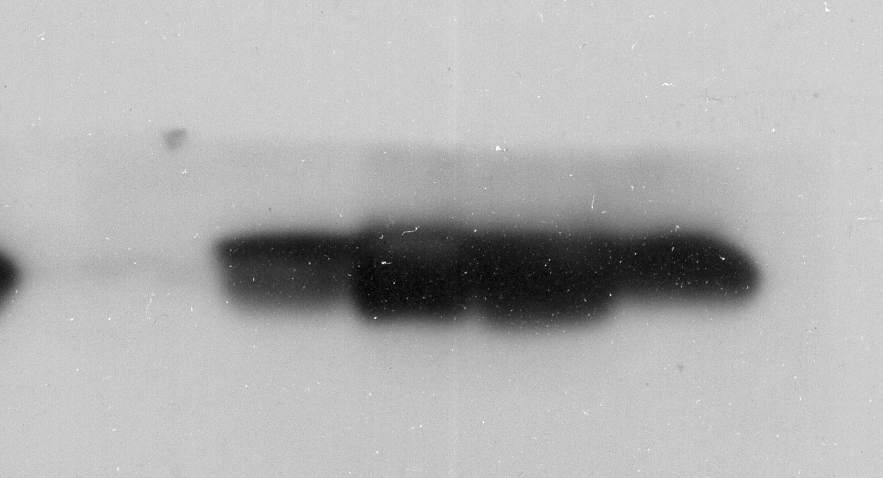

Supplement: Supplementary file 4 — Dataset 3 [file 41417_2024_729_MOESM4_ESM.zip › Figure 3/Figure 3d/myc-irs2 nibinding input n8.tif]

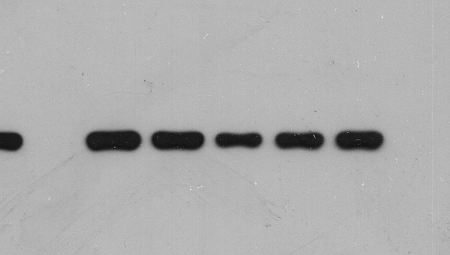

Supplement: Supplementary file 4 — Dataset 3 [file 41417_2024_729_MOESM4_ESM.zip › Figure 3/Figure 3d/myc-irs2 nibinding input tub.tif]

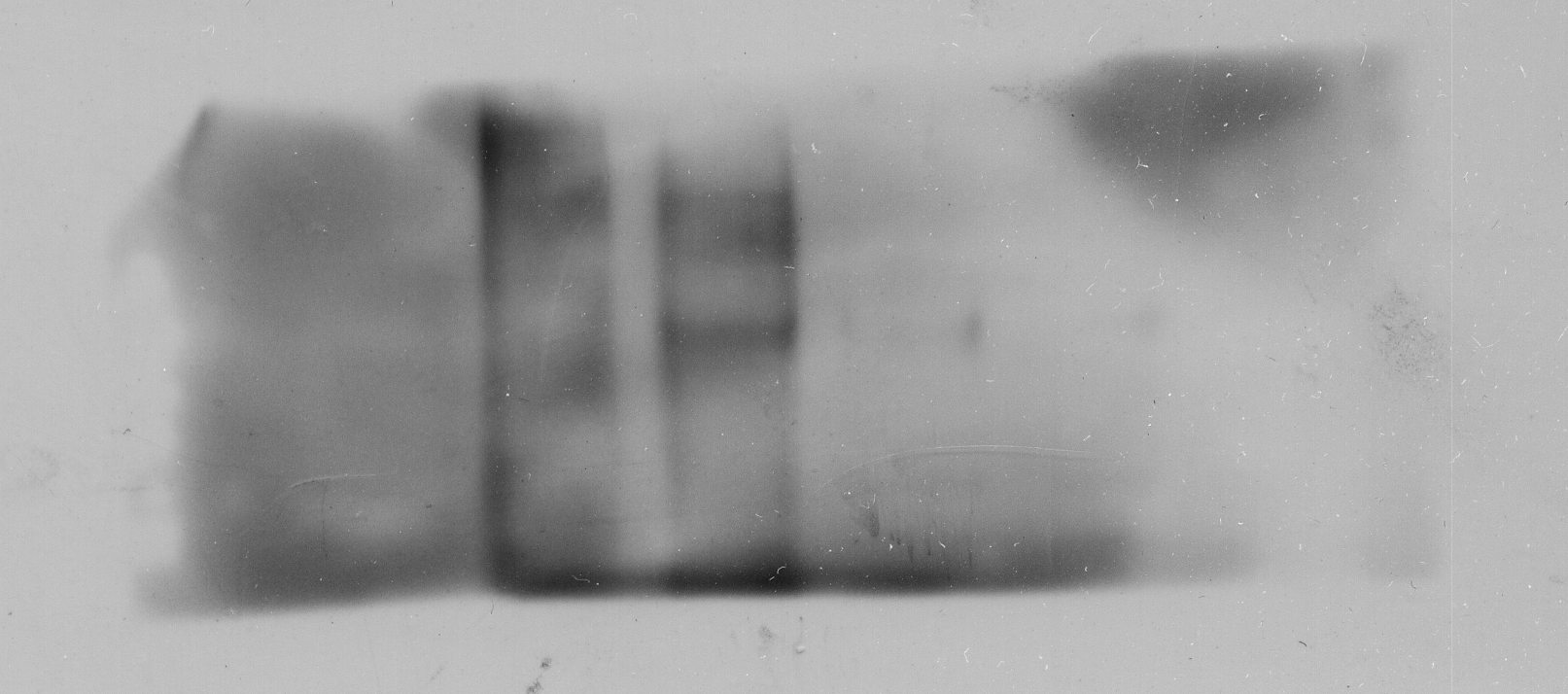

Supplement: Supplementary file 4 — Dataset 3 [file 41417_2024_729_MOESM4_ESM.zip › Figure 3/Figure 3d/myc-irs2 nibinding irs2.tif]

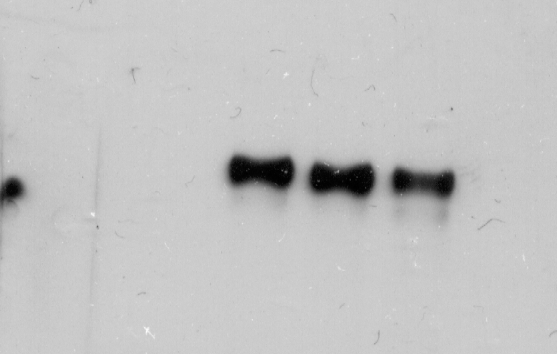

Supplement: Supplementary file 4 — Dataset 3 [file 41417_2024_729_MOESM4_ESM.zip › Figure 3/Figure 3e/flagirs1 input irs1.tif]

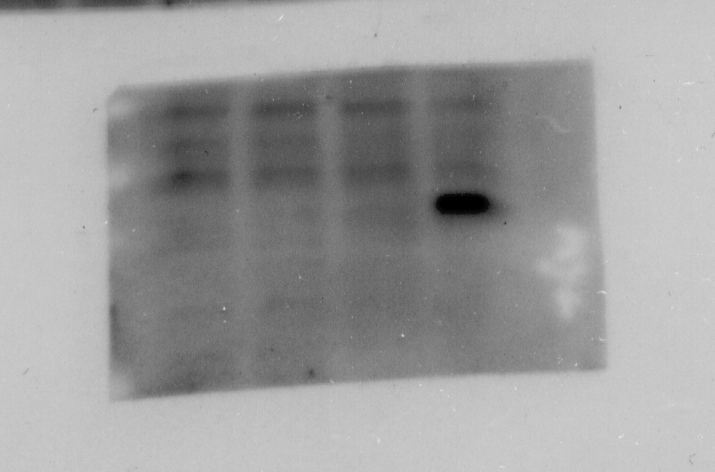

Supplement: Supplementary file 4 — Dataset 3 [file 41417_2024_729_MOESM4_ESM.zip › Figure 3/Figure 3e/flagirs1 input myc.tif]

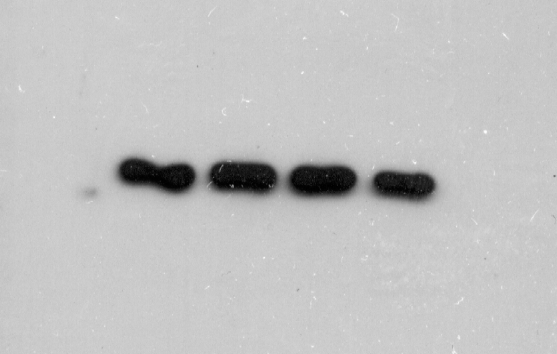

Supplement: Supplementary file 4 — Dataset 3 [file 41417_2024_729_MOESM4_ESM.zip › Figure 3/Figure 3e/flagirs1 input tub.tif]

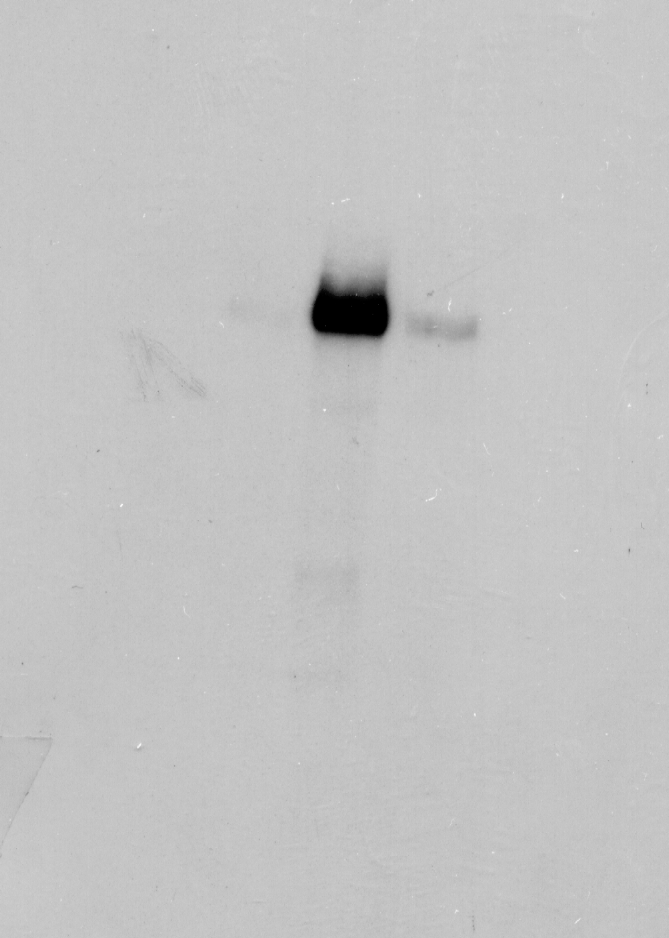

Supplement: Supplementary file 4 — Dataset 3 [file 41417_2024_729_MOESM4_ESM.zip › Figure 3/Figure 3e/haub ip irs1.tif]

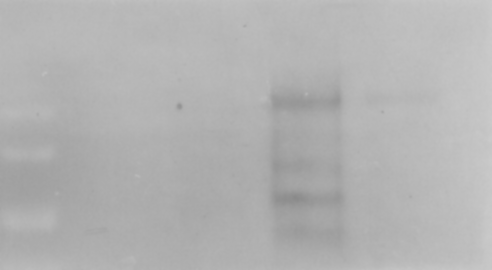

Supplement: Supplementary file 4 — Dataset 3 [file 41417_2024_729_MOESM4_ESM.zip › Figure 3/Figure 3e/haub ip irs2.tif]

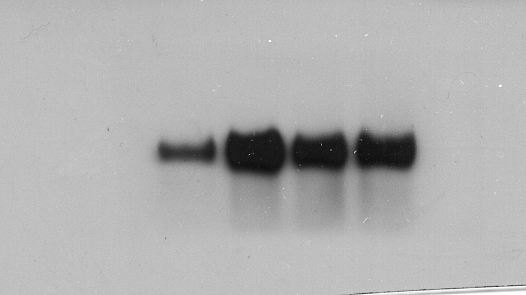

Supplement: Supplementary file 4 — Dataset 3 [file 41417_2024_729_MOESM4_ESM.zip › Figure 3/Figure 3e/mycirs2 input irs2.tif]

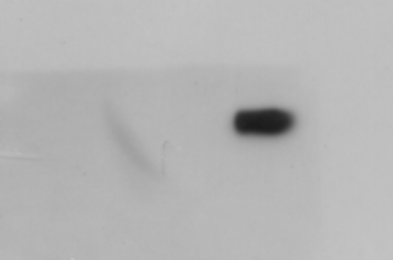

Supplement: Supplementary file 4 — Dataset 3 [file 41417_2024_729_MOESM4_ESM.zip › Figure 3/Figure 3e/mycirs2 input senp8.tif]

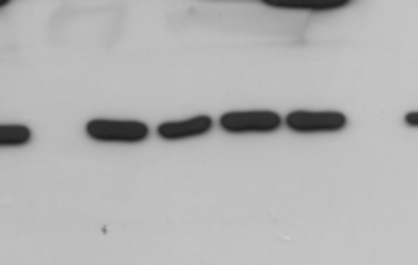

Supplement: Supplementary file 4 — Dataset 3 [file 41417_2024_729_MOESM4_ESM.zip › Figure 3/Figure 3e/mycirs2 input tub.tif]

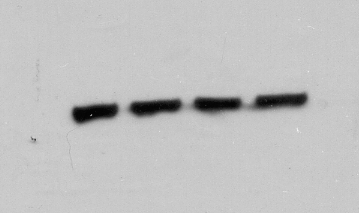

Supplement: Supplementary file 4 — Dataset 3 [file 41417_2024_729_MOESM4_ESM.zip › Figure 3/Figure 3f/011023 293t its2haubmln ip input tub135.tif]

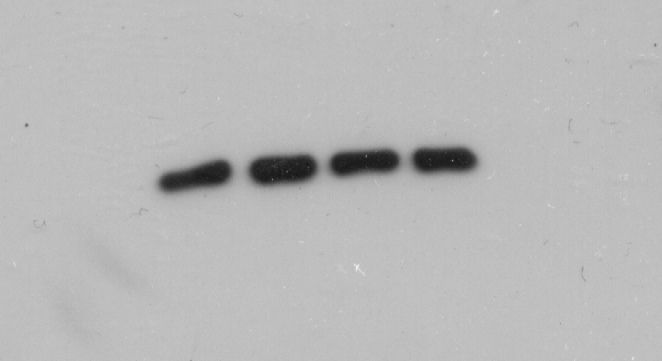

Supplement: Supplementary file 4 — Dataset 3 [file 41417_2024_729_MOESM4_ESM.zip › Figure 3/Figure 3f/flag-irs1 tub input.tif]

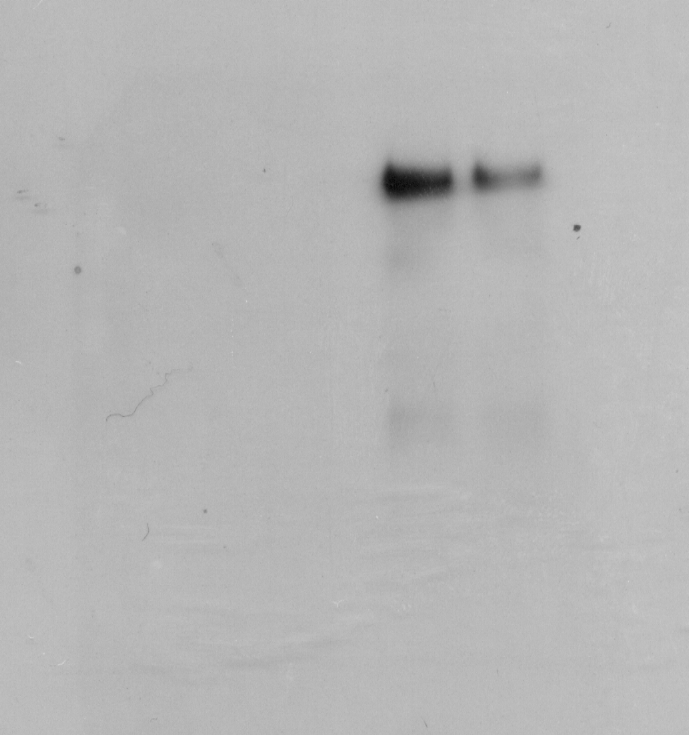

Supplement: Supplementary file 4 — Dataset 3 [file 41417_2024_729_MOESM4_ESM.zip › Figure 3/Figure 3f/haubmln irs1.tif]

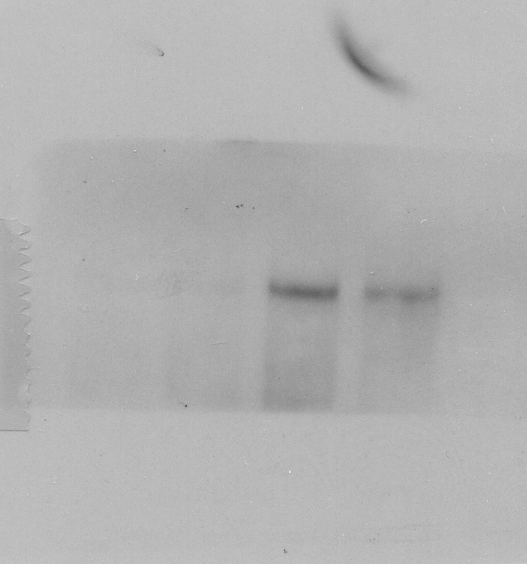

Supplement: Supplementary file 4 — Dataset 3 [file 41417_2024_729_MOESM4_ESM.zip › Figure 3/Figure 3f/haubmln irs2.tif]

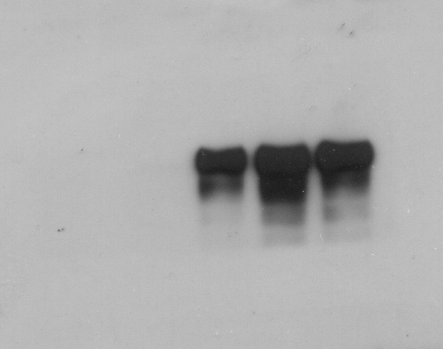

Supplement: Supplementary file 4 — Dataset 3 [file 41417_2024_729_MOESM4_ESM.zip › Figure 3/Figure 3f/irs1 input.tif]

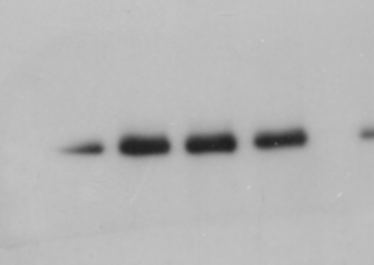

Supplement: Supplementary file 4 — Dataset 3 [file 41417_2024_729_MOESM4_ESM.zip › Figure 3/Figure 3f/irs2 input.tif]

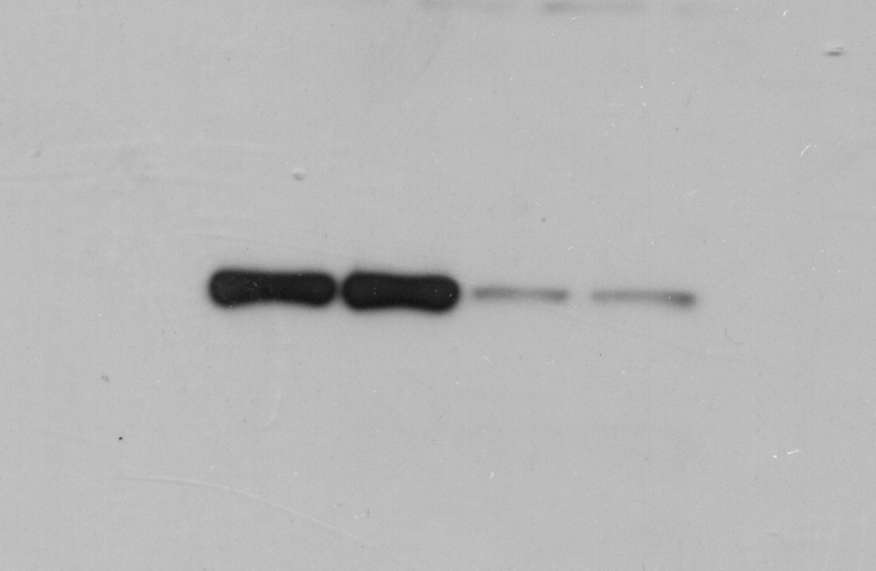

Supplement: Supplementary file 5 — Dataset 4 [file 41417_2024_729_MOESM5_ESM.zip › Figure 4/Figure 4a/rcc/rcc cbl.tif]

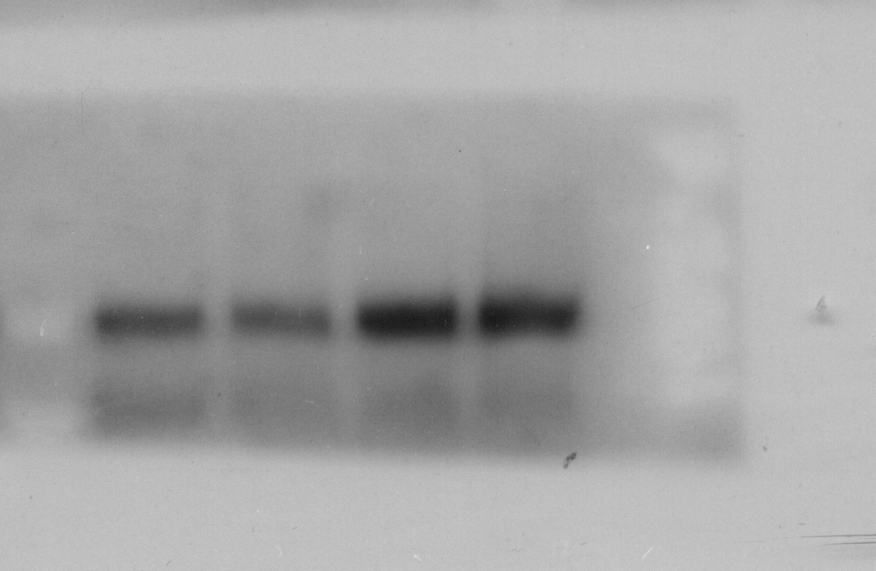

Supplement: Supplementary file 5 — Dataset 4 [file 41417_2024_729_MOESM5_ESM.zip › Figure 4/Figure 4a/rcc/rcc irs1.tif]

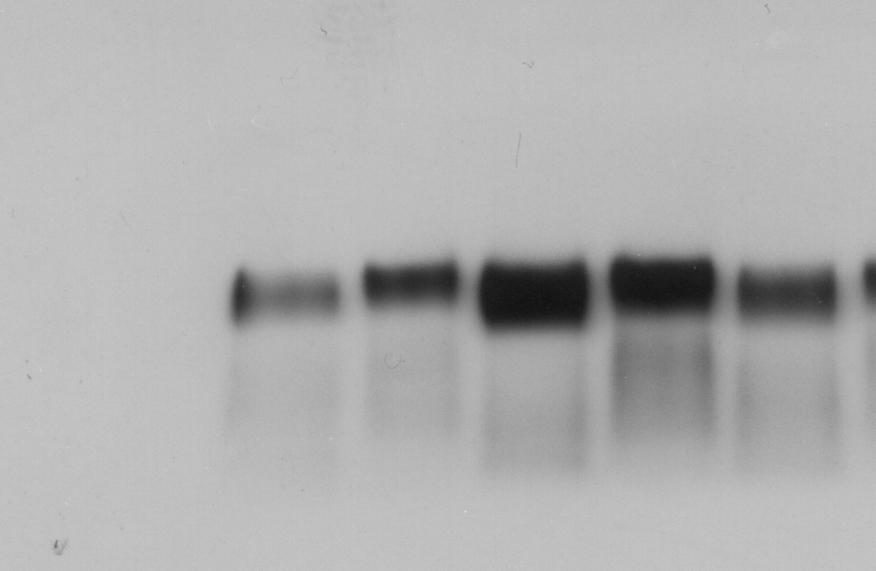

Supplement: Supplementary file 5 — Dataset 4 [file 41417_2024_729_MOESM5_ESM.zip › Figure 4/Figure 4a/rcc/rcc irs2.tif]

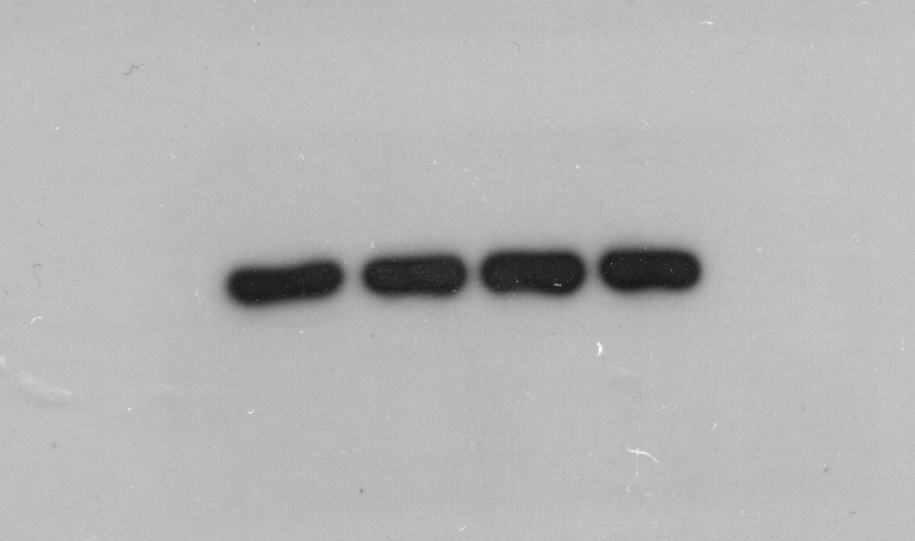

Supplement: Supplementary file 5 — Dataset 4 [file 41417_2024_729_MOESM5_ESM.zip › Figure 4/Figure 4a/rcc/rcc tub.tif]

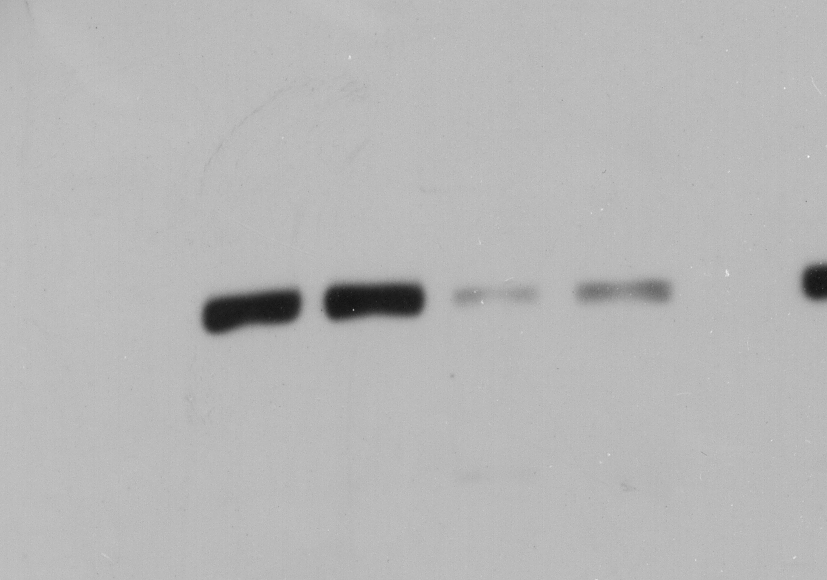

Supplement: Supplementary file 5 — Dataset 4 [file 41417_2024_729_MOESM5_ESM.zip › Figure 4/Figure 4a/skov/skov3 cbl.tif]

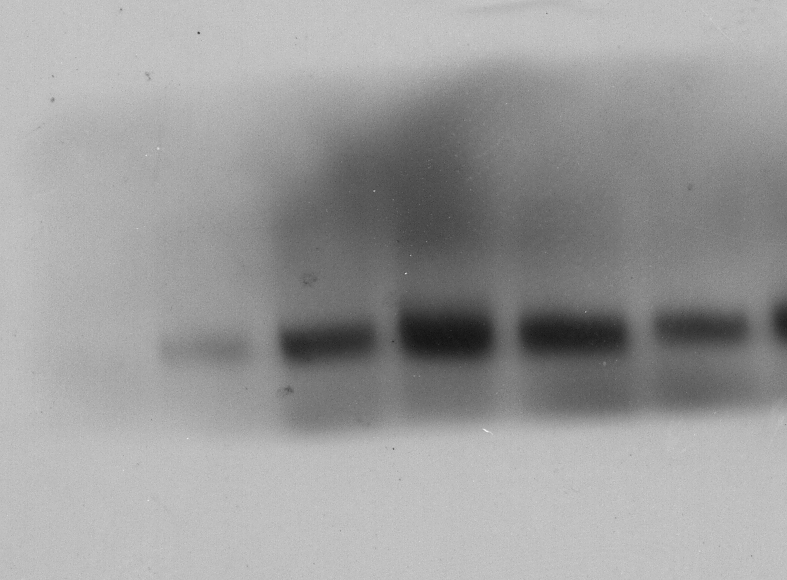

Supplement: Supplementary file 5 — Dataset 4 [file 41417_2024_729_MOESM5_ESM.zip › Figure 4/Figure 4a/skov/skov3 irs1.tif]

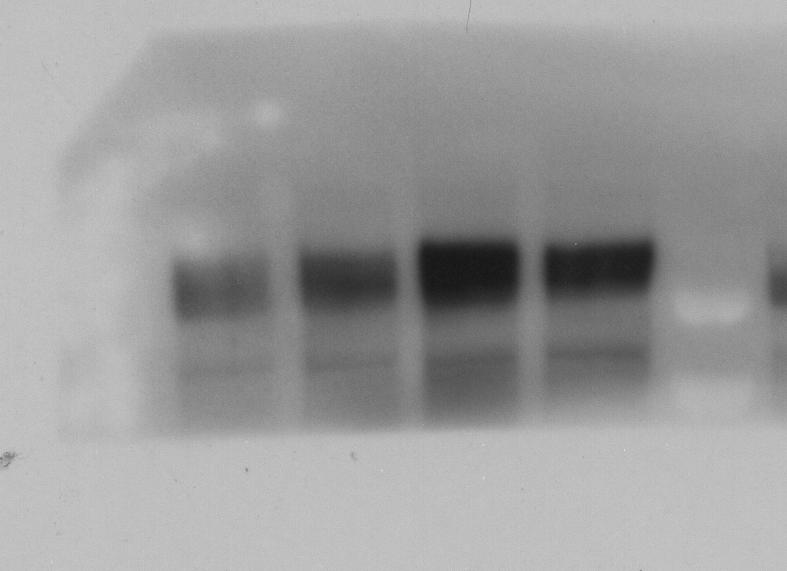

Supplement: Supplementary file 5 — Dataset 4 [file 41417_2024_729_MOESM5_ESM.zip › Figure 4/Figure 4a/skov/skov3 irs2.tif]

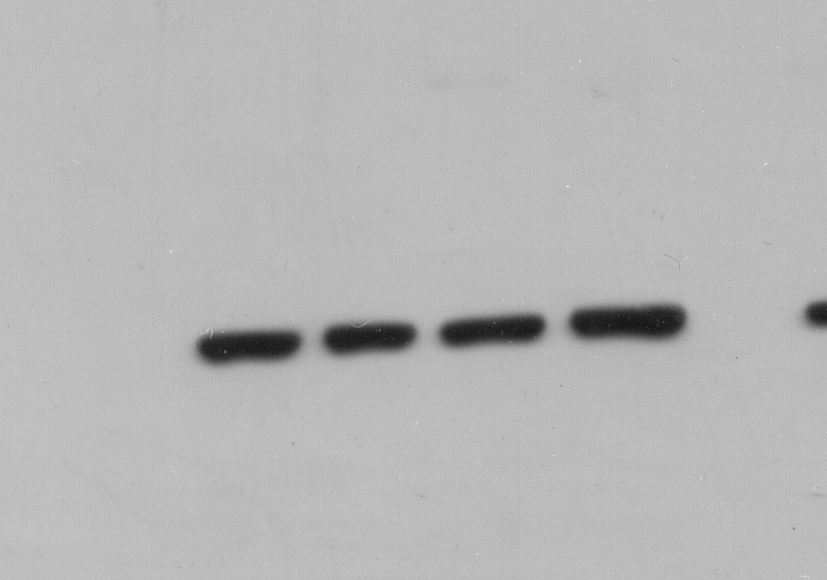

Supplement: Supplementary file 5 — Dataset 4 [file 41417_2024_729_MOESM5_ESM.zip › Figure 4/Figure 4a/skov/skov3 tub.tif]

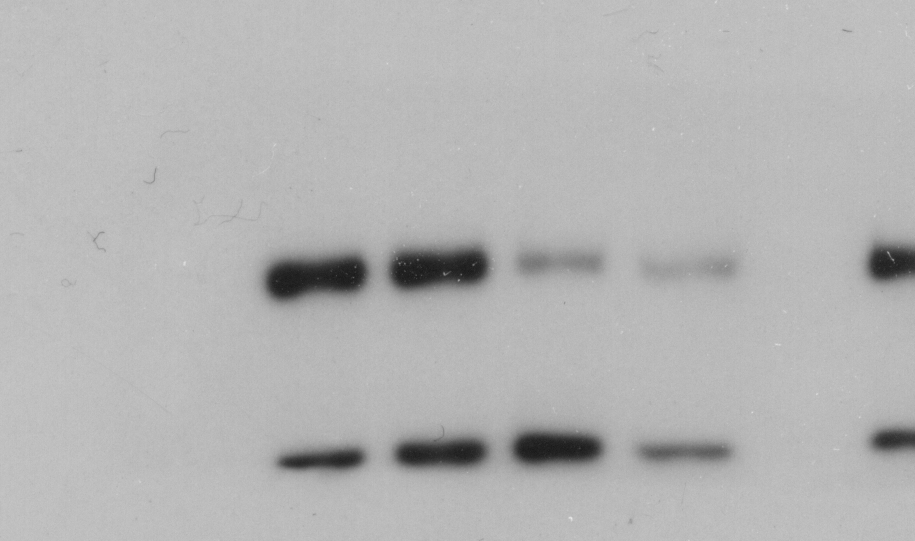

Supplement: Supplementary file 5 — Dataset 4 [file 41417_2024_729_MOESM5_ESM.zip › Figure 4/Figure 4a/u373/u373 cbl.tif]

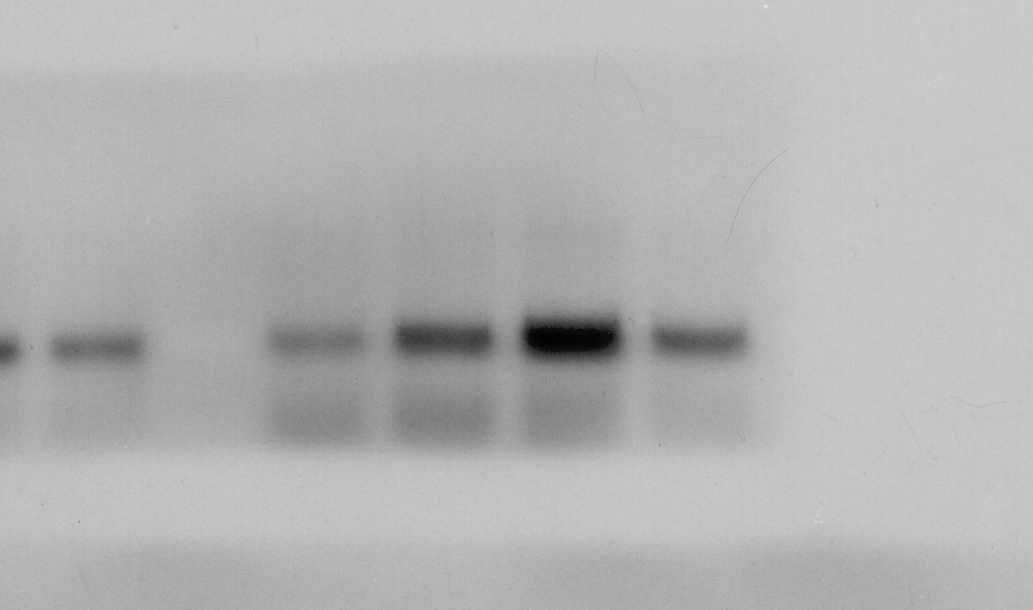

Supplement: Supplementary file 5 — Dataset 4 [file 41417_2024_729_MOESM5_ESM.zip › Figure 4/Figure 4a/u373/u373 irs1.tif]

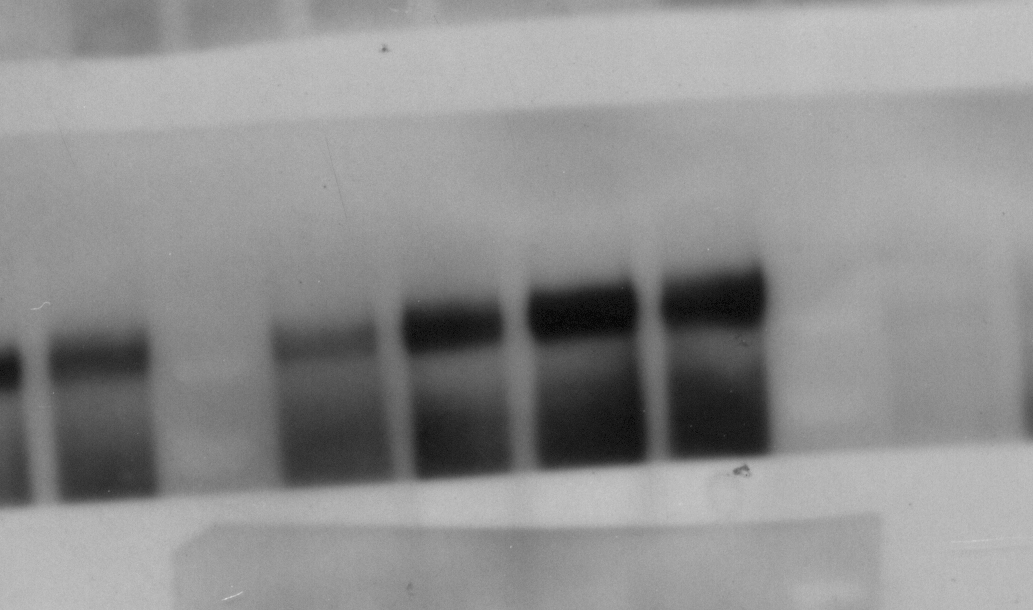

Supplement: Supplementary file 5 — Dataset 4 [file 41417_2024_729_MOESM5_ESM.zip › Figure 4/Figure 4a/u373/u373 irs2.tif]

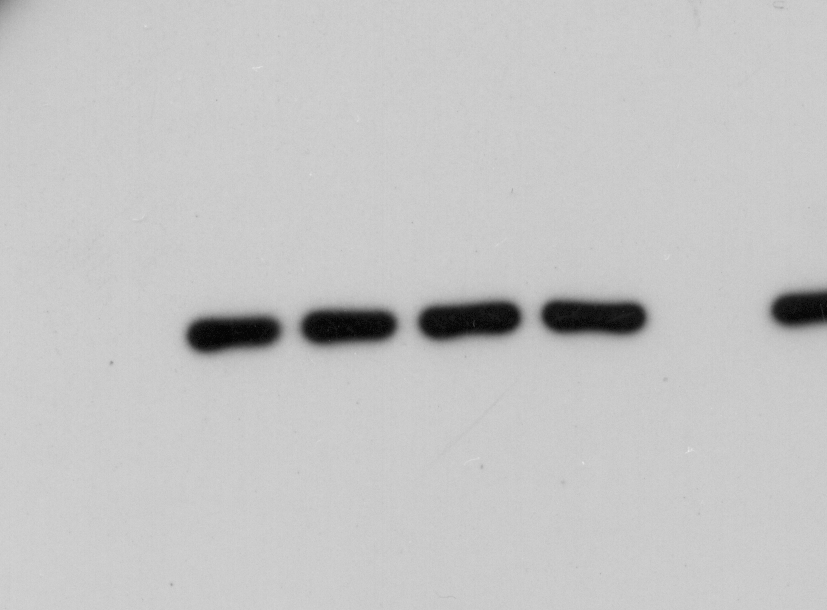

Supplement: Supplementary file 5 — Dataset 4 [file 41417_2024_729_MOESM5_ESM.zip › Figure 4/Figure 4a/u373/u373 tub.tif]

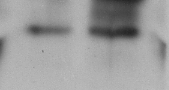

Supplement: Supplementary file 5 — Dataset 4 [file 41417_2024_729_MOESM5_ESM.zip › Figure 4/Figure 4b/flag cbl input tub.tif]

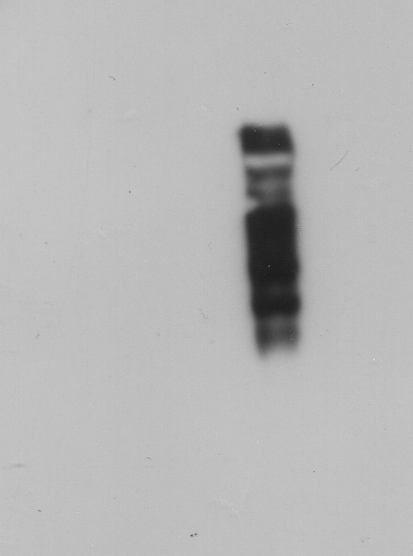

Supplement: Supplementary file 5 — Dataset 4 [file 41417_2024_729_MOESM5_ESM.zip › Figure 4/Figure 4b/flag cbl ip flag.tif]

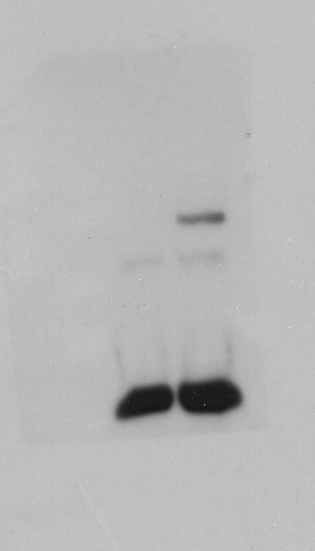

Supplement: Supplementary file 5 — Dataset 4 [file 41417_2024_729_MOESM5_ESM.zip › Figure 4/Figure 4b/flag cbl ip irs1.tif]

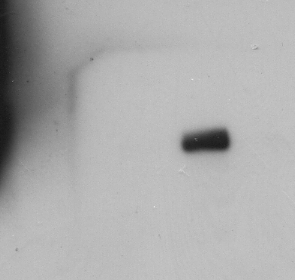

Supplement: Supplementary file 5 — Dataset 4 [file 41417_2024_729_MOESM5_ESM.zip › Figure 4/Figure 4b/flag irs1 input irs1.tif]

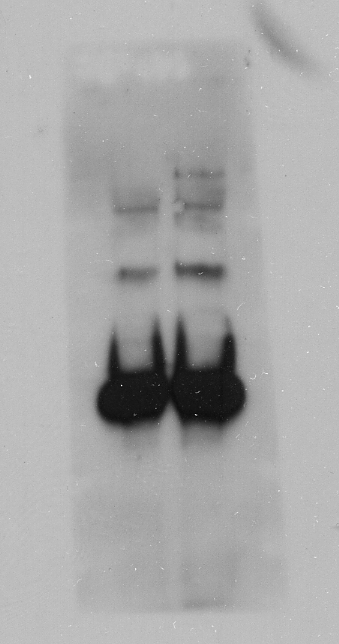

Supplement: Supplementary file 5 — Dataset 4 [file 41417_2024_729_MOESM5_ESM.zip › Figure 4/Figure 4b/flag irs1 ip cbl.tif]

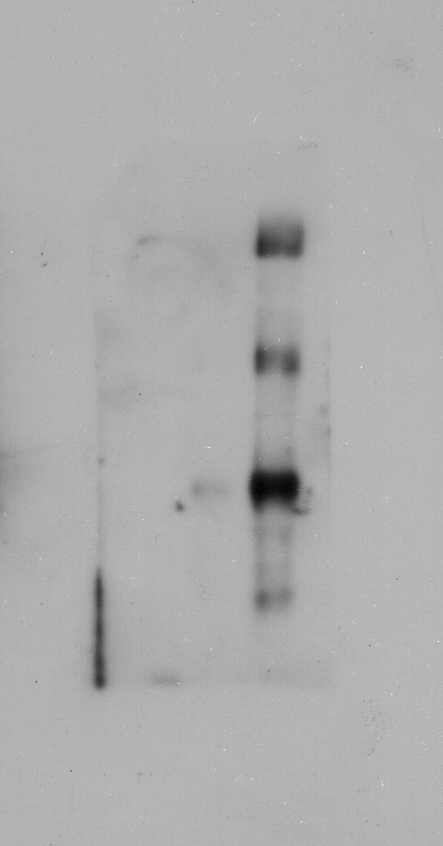

Supplement: Supplementary file 5 — Dataset 4 [file 41417_2024_729_MOESM5_ESM.zip › Figure 4/Figure 4b/flag irs1 ip flag.tif]

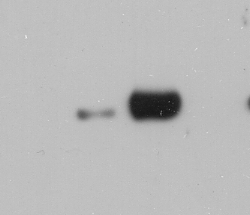

Supplement: Supplementary file 5 — Dataset 4 [file 41417_2024_729_MOESM5_ESM.zip › Figure 4/Figure 4b/flagcbl input cbl.tif]

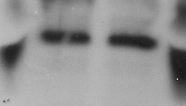

Supplement: Supplementary file 5 — Dataset 4 [file 41417_2024_729_MOESM5_ESM.zip › Figure 4/Figure 4b/flagirs1 input tub.tif]

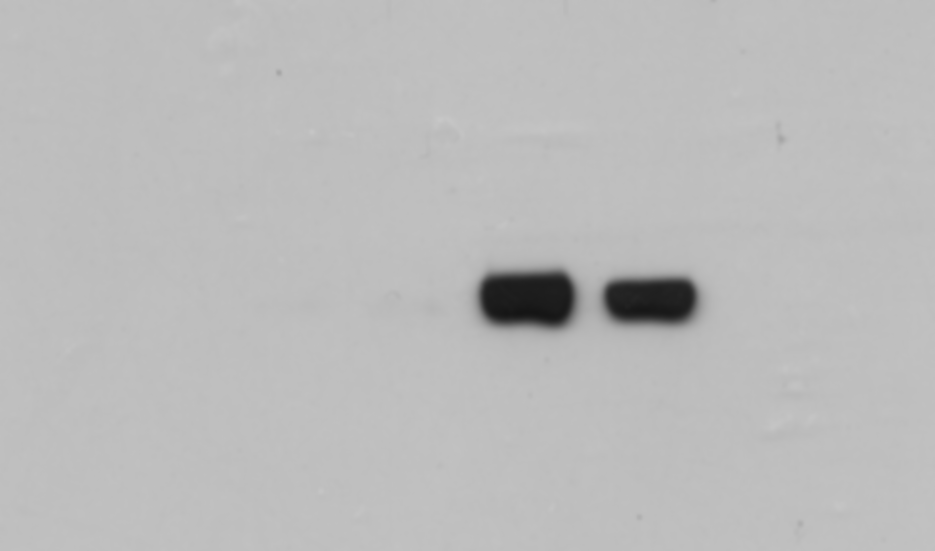

Supplement: Supplementary file 5 — Dataset 4 [file 41417_2024_729_MOESM5_ESM.zip › Figure 4/Figure 4c/ccbl input.tif]

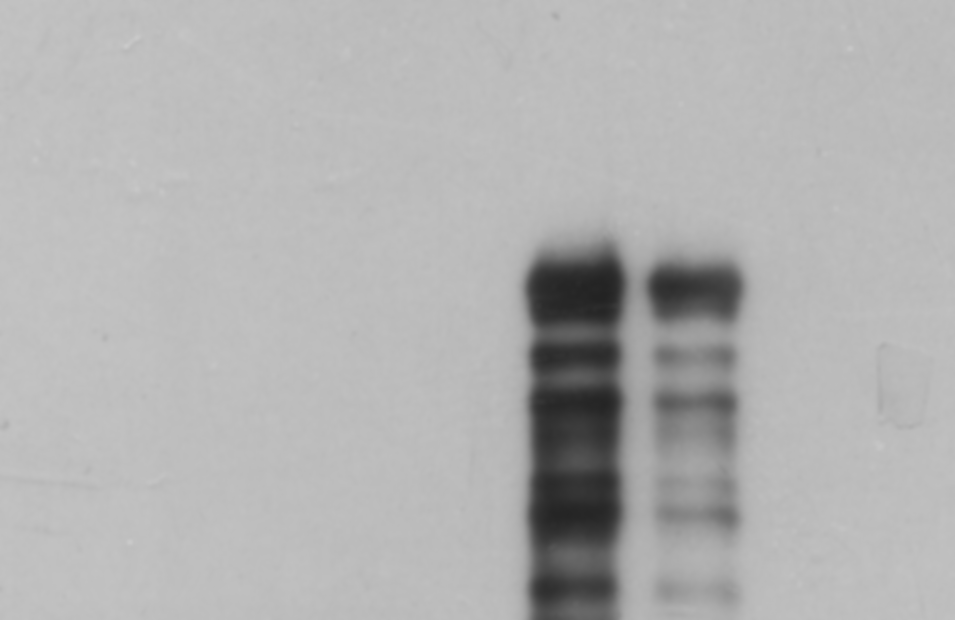

Supplement: Supplementary file 5 — Dataset 4 [file 41417_2024_729_MOESM5_ESM.zip › Figure 4/Figure 4c/ip flag.tif]

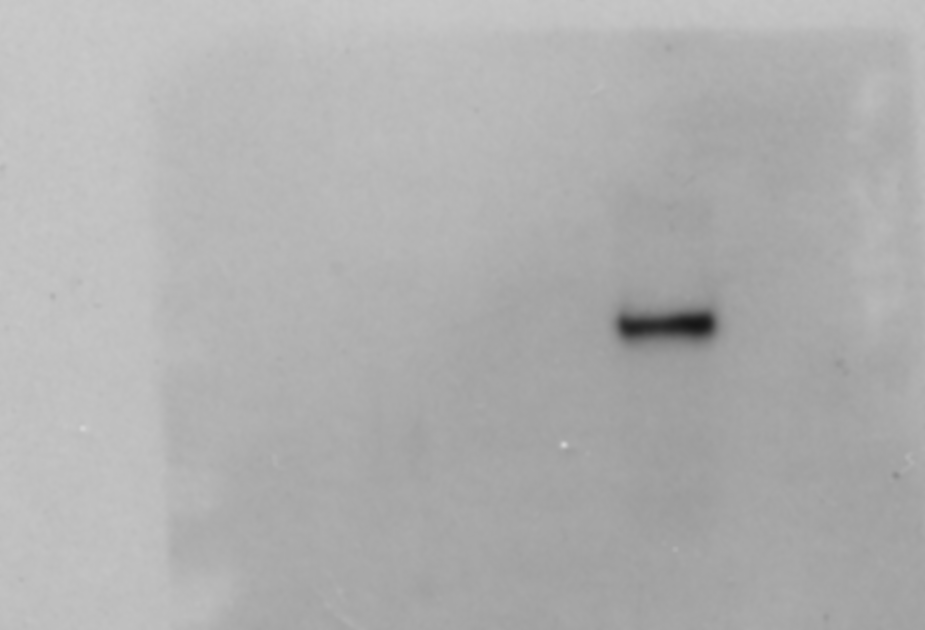

Supplement: Supplementary file 5 — Dataset 4 [file 41417_2024_729_MOESM5_ESM.zip › Figure 4/Figure 4c/ip irs2.tif]

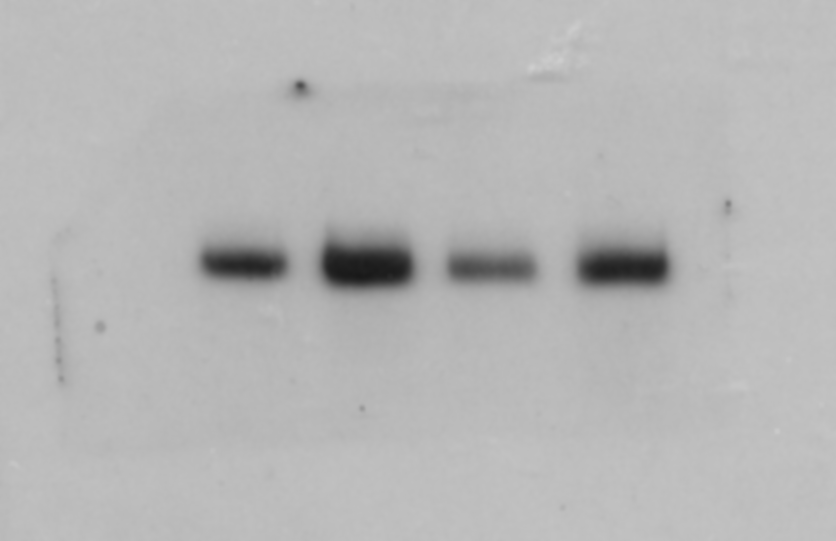

Supplement: Supplementary file 5 — Dataset 4 [file 41417_2024_729_MOESM5_ESM.zip › Figure 4/Figure 4c/irs2 input.tif]

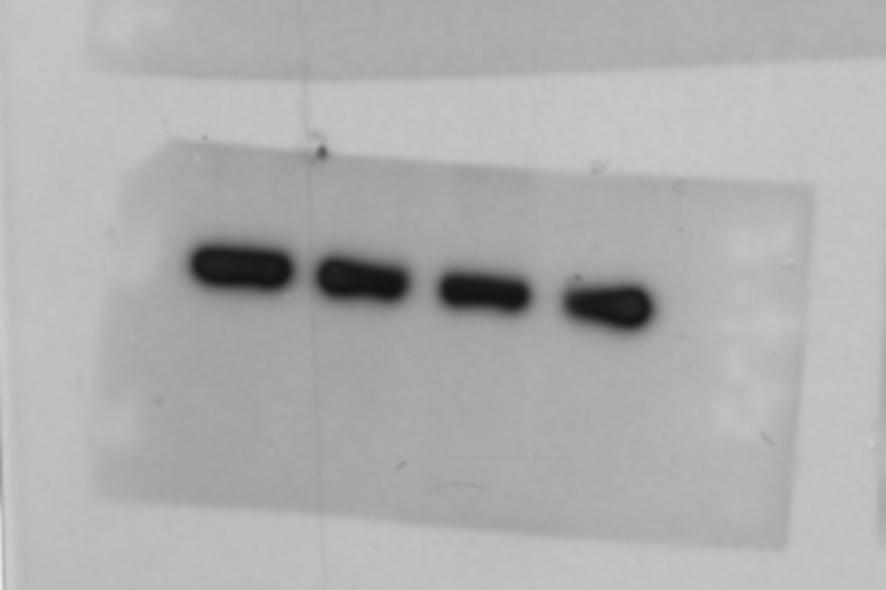

Supplement: Supplementary file 5 — Dataset 4 [file 41417_2024_729_MOESM5_ESM.zip › Figure 4/Figure 4c/tub input.tif]

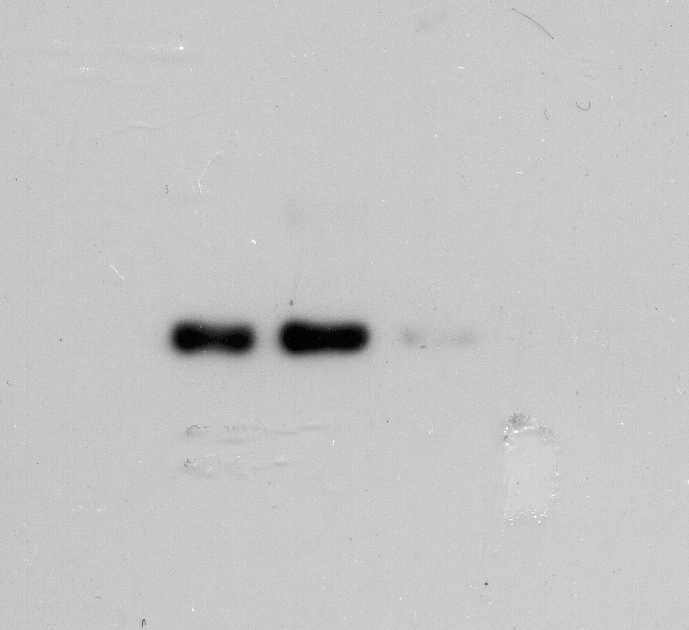

Supplement: Supplementary file 5 — Dataset 4 [file 41417_2024_729_MOESM5_ESM.zip › Figure 4/Figure 4d/input cbl for irs1 ip.tif]

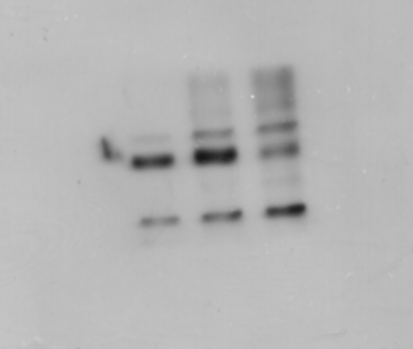

Supplement: Supplementary file 5 — Dataset 4 [file 41417_2024_729_MOESM5_ESM.zip › Figure 4/Figure 4d/input cbl for irs2 ip.tif]

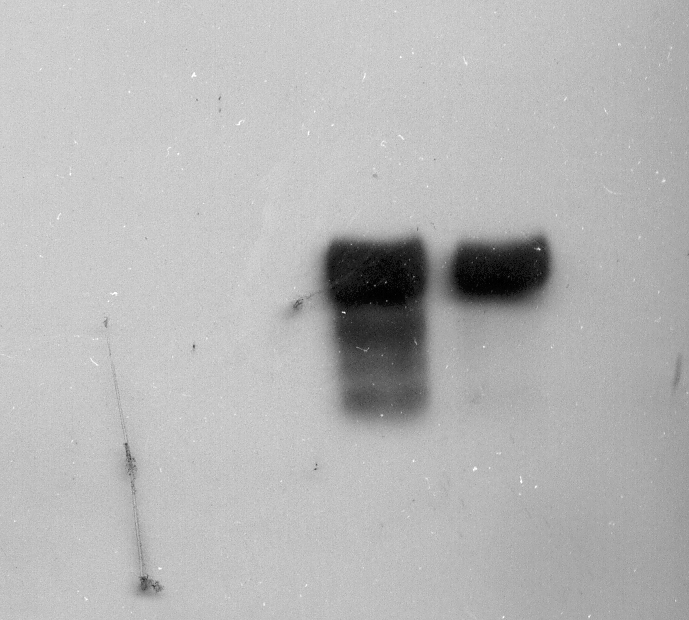

Supplement: Supplementary file 5 — Dataset 4 [file 41417_2024_729_MOESM5_ESM.zip › Figure 4/Figure 4d/input irs1.tif]

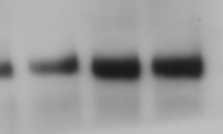

Supplement: Supplementary file 5 — Dataset 4 [file 41417_2024_729_MOESM5_ESM.zip › Figure 4/Figure 4d/input irs2.tif]

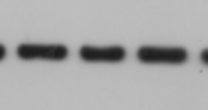

Supplement: Supplementary file 5 — Dataset 4 [file 41417_2024_729_MOESM5_ESM.zip › Figure 4/Figure 4d/input tub for irs2.tif]

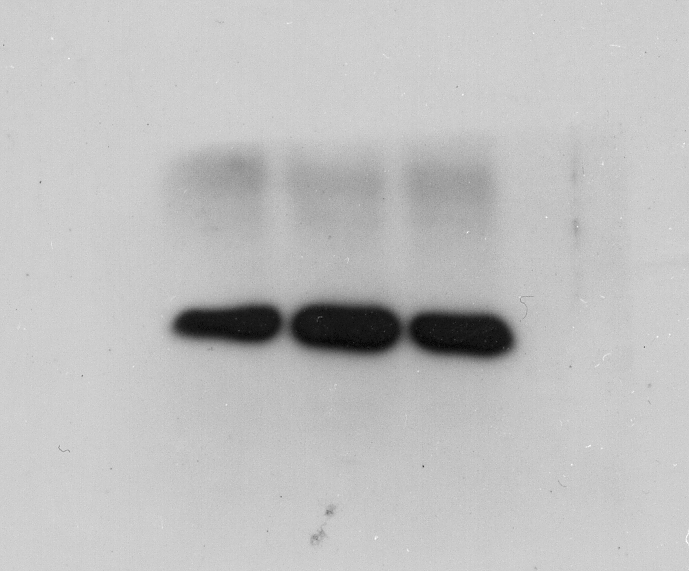

Supplement: Supplementary file 5 — Dataset 4 [file 41417_2024_729_MOESM5_ESM.zip › Figure 4/Figure 4d/input tub.tif]

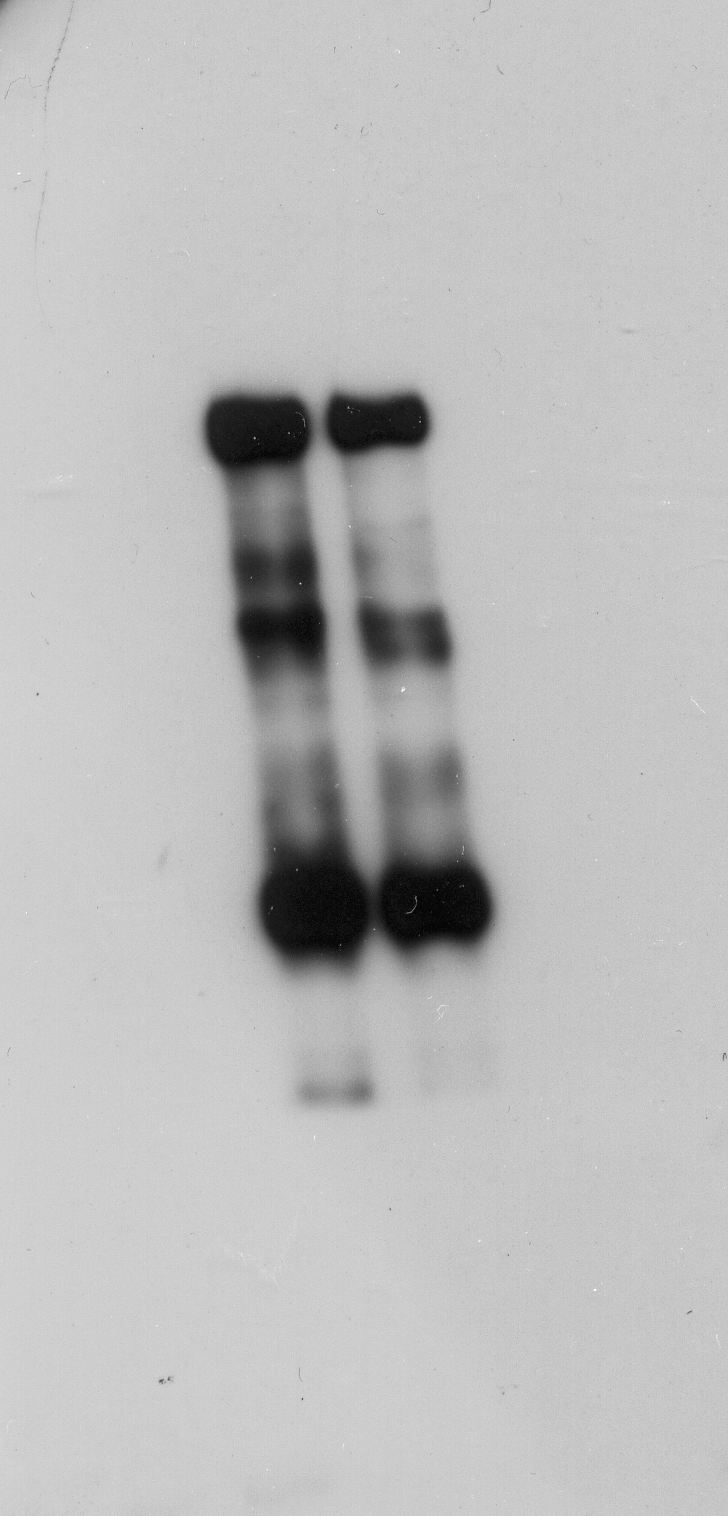

Supplement: Supplementary file 5 — Dataset 4 [file 41417_2024_729_MOESM5_ESM.zip › Figure 4/Figure 4d/ip flag irs1.tif]

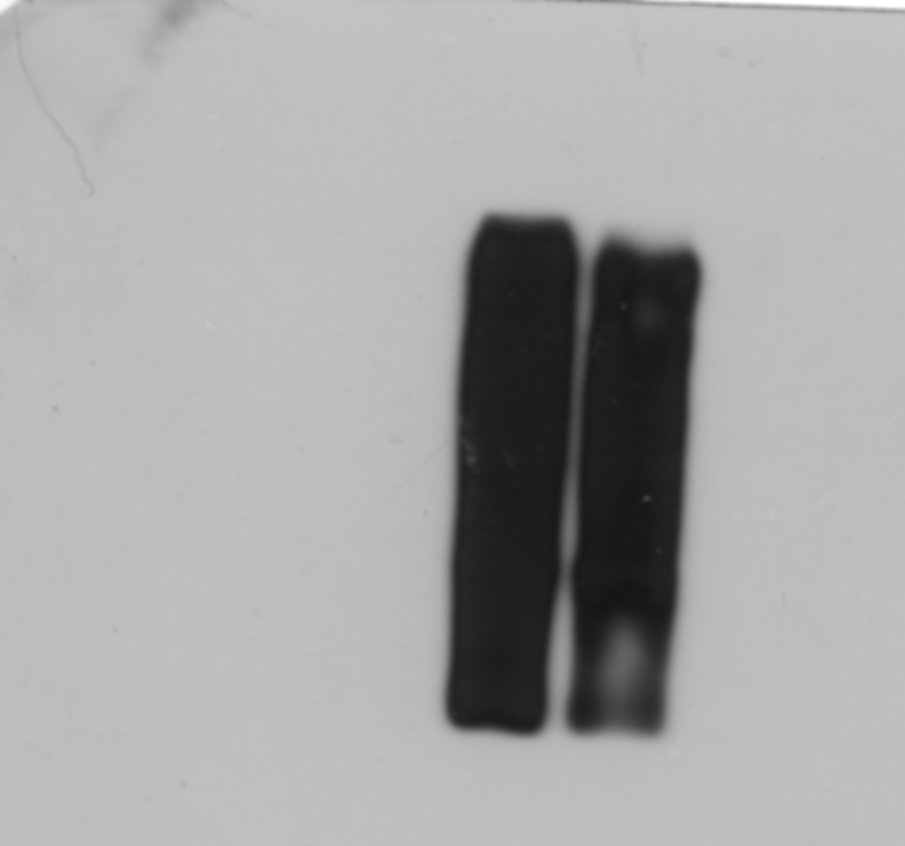

Supplement: Supplementary file 5 — Dataset 4 [file 41417_2024_729_MOESM5_ESM.zip › Figure 4/Figure 4d/ip flag n8.tif]

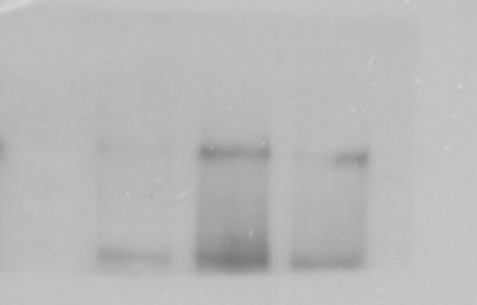

Supplement: Supplementary file 5 — Dataset 4 [file 41417_2024_729_MOESM5_ESM.zip › Figure 4/Figure 4d/ip irs2.tif]

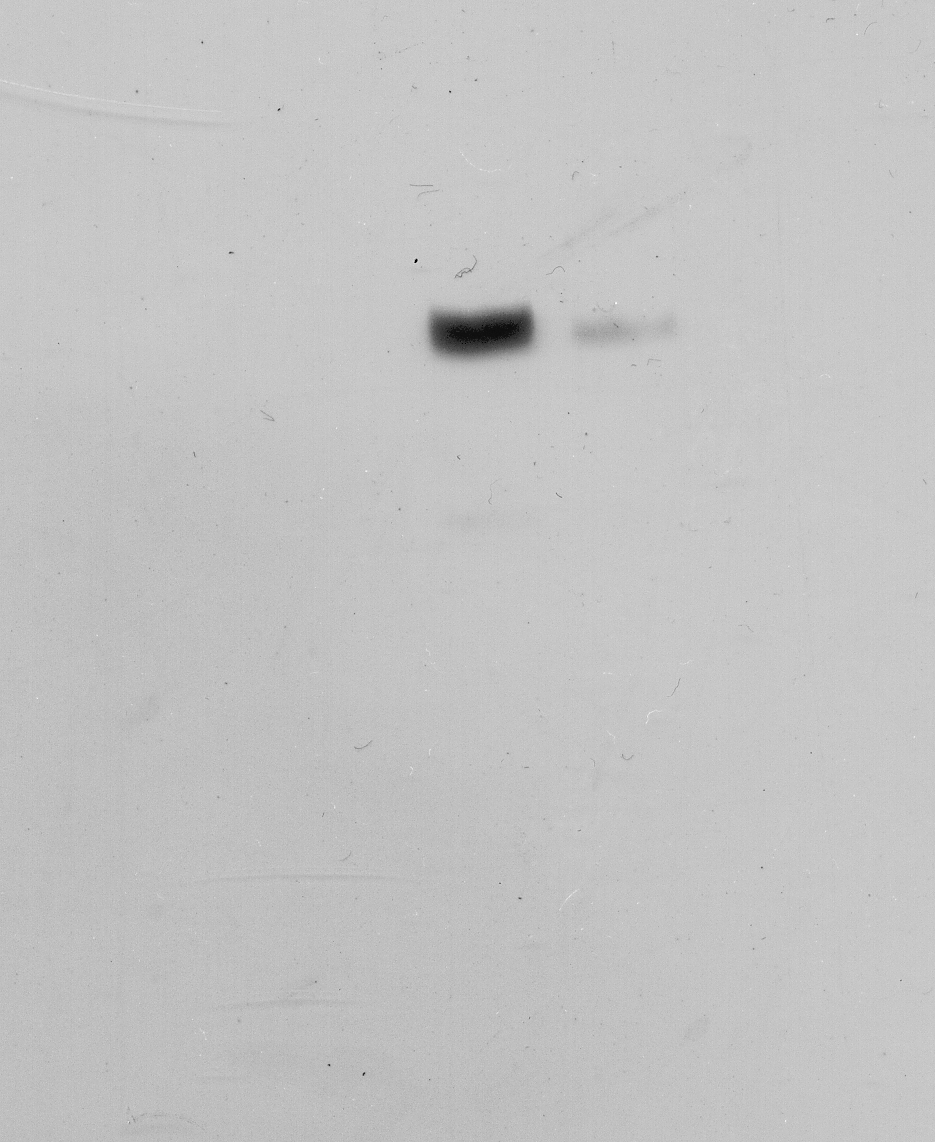

Supplement: Supplementary file 5 — Dataset 4 [file 41417_2024_729_MOESM5_ESM.zip › Figure 4/Figure 4d/ip n8.tif]

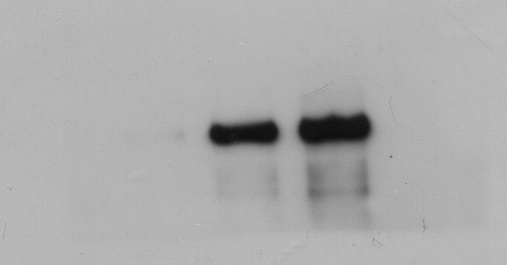

Supplement: Supplementary file 5 — Dataset 4 [file 41417_2024_729_MOESM5_ESM.zip › Figure 4/Figure 4e/rcc4/endo irs1.tif]

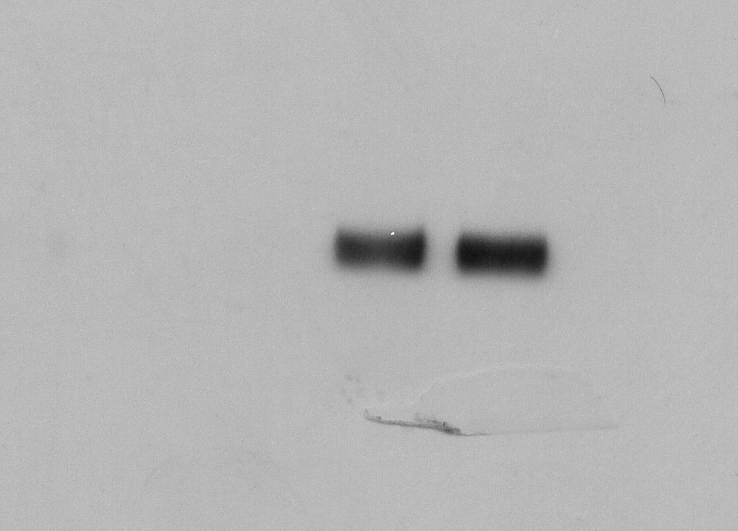

Supplement: Supplementary file 5 — Dataset 4 [file 41417_2024_729_MOESM5_ESM.zip › Figure 4/Figure 4e/rcc4/endo irs2.tif]

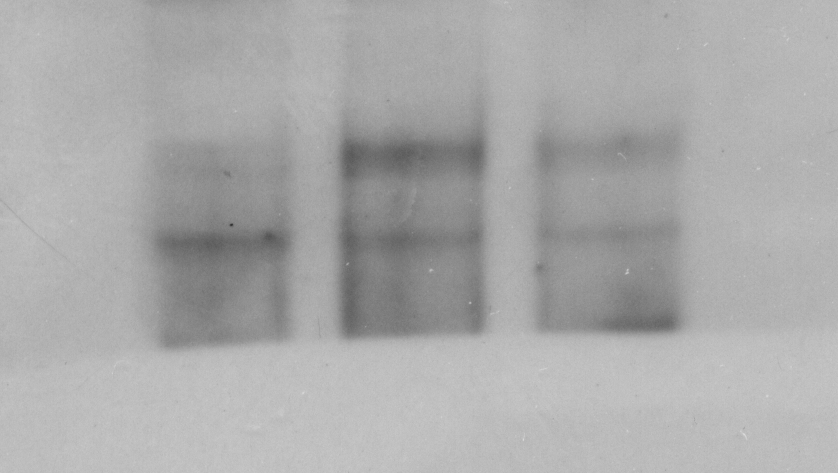

Supplement: Supplementary file 5 — Dataset 4 [file 41417_2024_729_MOESM5_ESM.zip › Figure 4/Figure 4e/rcc4/endo n8 for irs1.tif]

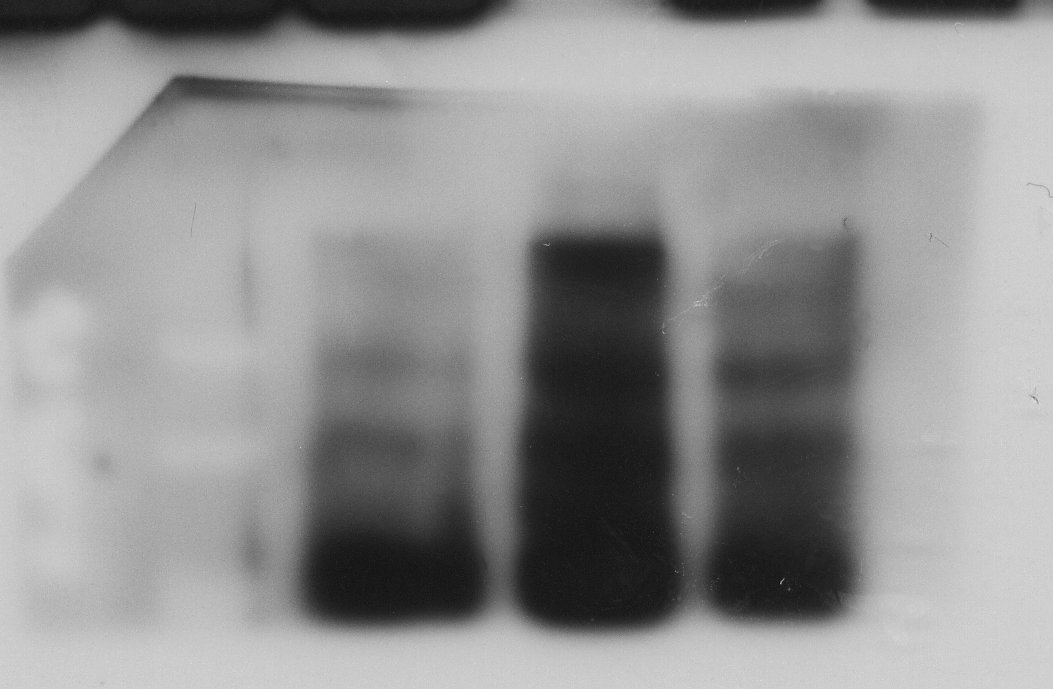

Supplement: Supplementary file 5 — Dataset 4 [file 41417_2024_729_MOESM5_ESM.zip › Figure 4/Figure 4e/rcc4/endo n8 for irs2.tif]

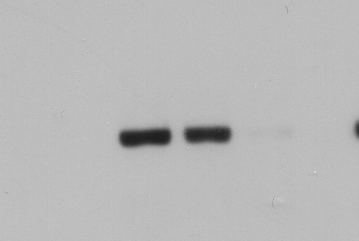

Supplement: Supplementary file 5 — Dataset 4 [file 41417_2024_729_MOESM5_ESM.zip › Figure 4/Figure 4e/rcc4/input cbl for irs1.tif]

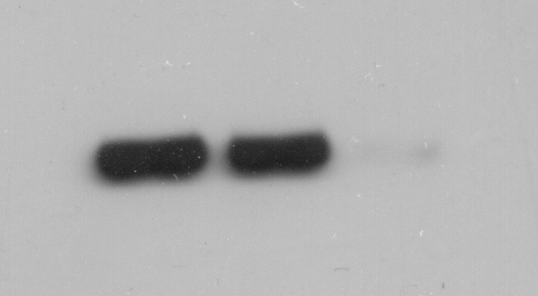

Supplement: Supplementary file 5 — Dataset 4 [file 41417_2024_729_MOESM5_ESM.zip › Figure 4/Figure 4e/rcc4/input cbl for irs2.tif]

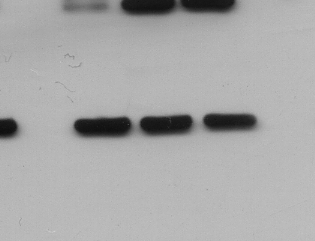

Supplement: Supplementary file 5 — Dataset 4 [file 41417_2024_729_MOESM5_ESM.zip › Figure 4/Figure 4e/rcc4/input tub for irs1.tif]

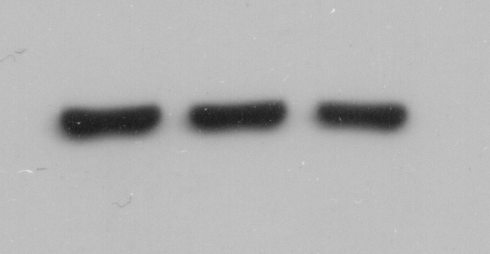

Supplement: Supplementary file 5 — Dataset 4 [file 41417_2024_729_MOESM5_ESM.zip › Figure 4/Figure 4e/rcc4/input tub for irs2.tif]

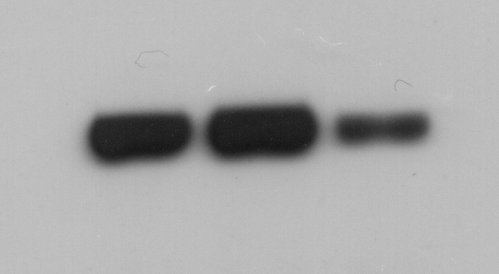

Supplement: Supplementary file 5 — Dataset 4 [file 41417_2024_729_MOESM5_ESM.zip › Figure 4/Figure 4e/skov/022123 skov3u373rcc4 sicblins endo ip input cbl062.tif]

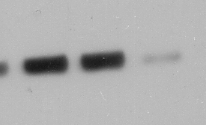

Supplement: Supplementary file 5 — Dataset 4 [file 41417_2024_729_MOESM5_ESM.zip › Figure 4/Figure 4e/skov/input cbl for irs1 endo.tif]

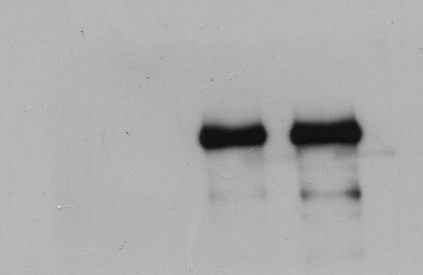

Supplement: Supplementary file 5 — Dataset 4 [file 41417_2024_729_MOESM5_ESM.zip › Figure 4/Figure 4e/skov/skov3 endo irs11.tif]

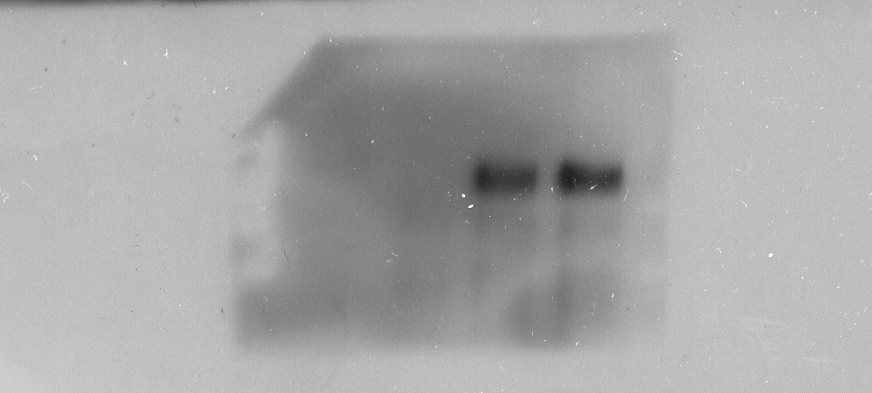

Supplement: Supplementary file 5 — Dataset 4 [file 41417_2024_729_MOESM5_ESM.zip › Figure 4/Figure 4e/skov/skov3 endo irs22.tif]

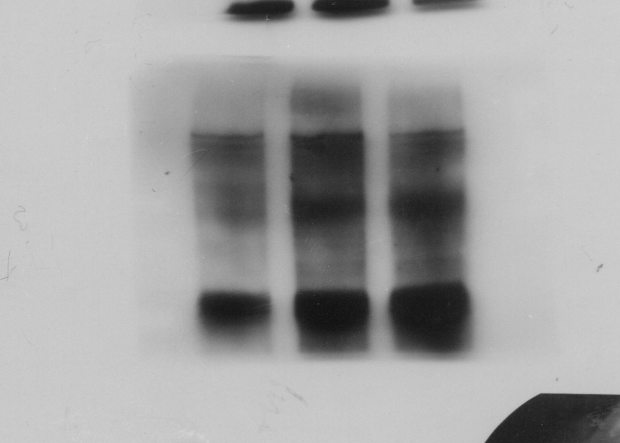

Supplement: Supplementary file 5 — Dataset 4 [file 41417_2024_729_MOESM5_ESM.zip › Figure 4/Figure 4e/skov/skov3 endo n8 for irs1.tif]

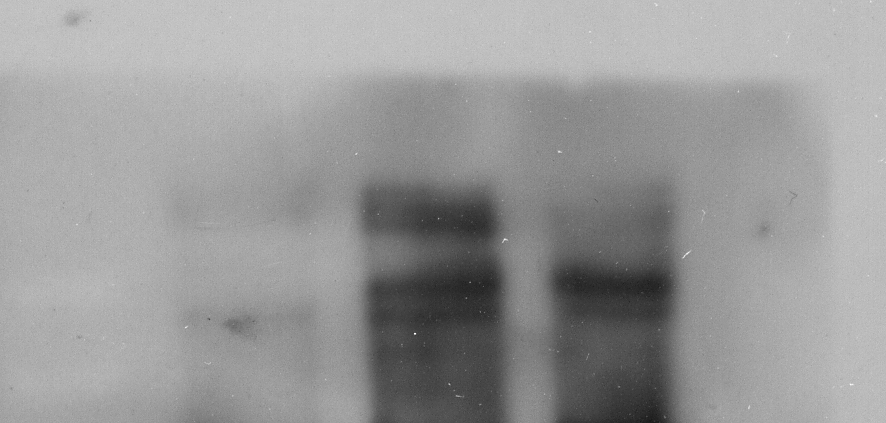

Supplement: Supplementary file 5 — Dataset 4 [file 41417_2024_729_MOESM5_ESM.zip › Figure 4/Figure 4e/skov/skov3 endo n8 for irs2.tif]

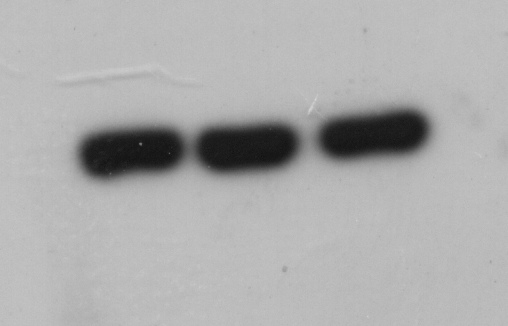

Supplement: Supplementary file 5 — Dataset 4 [file 41417_2024_729_MOESM5_ESM.zip › Figure 4/Figure 4e/skov/skov3 input tub for irs2.tif]

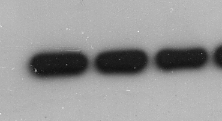

Supplement: Supplementary file 5 — Dataset 4 [file 41417_2024_729_MOESM5_ESM.zip › Figure 4/Figure 4e/skov/skov3 input tub.tif]

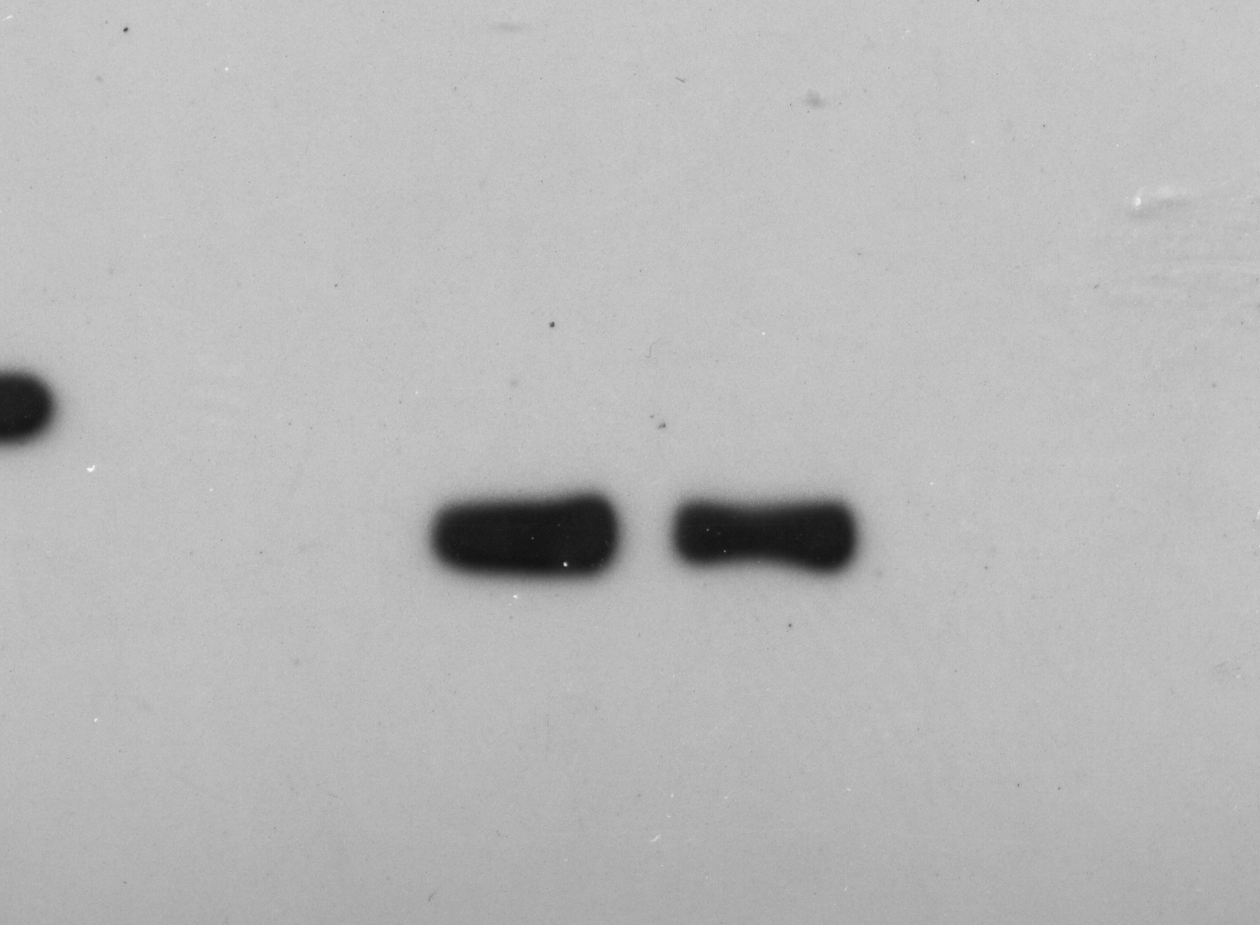

Supplement: Supplementary file 5 — Dataset 4 [file 41417_2024_729_MOESM5_ESM.zip › Figure 4/Figure 4e/u373/endo input cbl for irs1.tif]

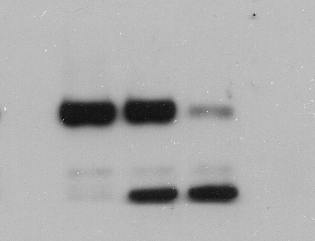

Supplement: Supplementary file 5 — Dataset 4 [file 41417_2024_729_MOESM5_ESM.zip › Figure 4/Figure 4e/u373/endo input cbl for irs2.tif]

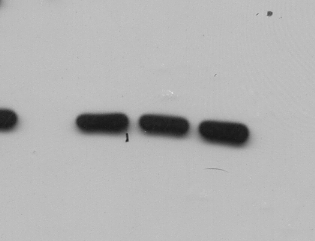

Supplement: Supplementary file 5 — Dataset 4 [file 41417_2024_729_MOESM5_ESM.zip › Figure 4/Figure 4e/u373/endo input tub for irs1.tif]

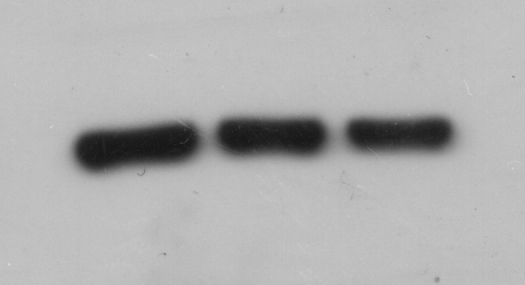

Supplement: Supplementary file 5 — Dataset 4 [file 41417_2024_729_MOESM5_ESM.zip › Figure 4/Figure 4e/u373/endo input tub for irs2.tif]

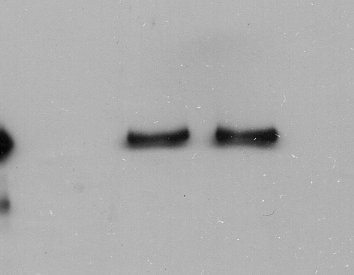

Supplement: Supplementary file 5 — Dataset 4 [file 41417_2024_729_MOESM5_ESM.zip › Figure 4/Figure 4e/u373/endo irs1.tif]

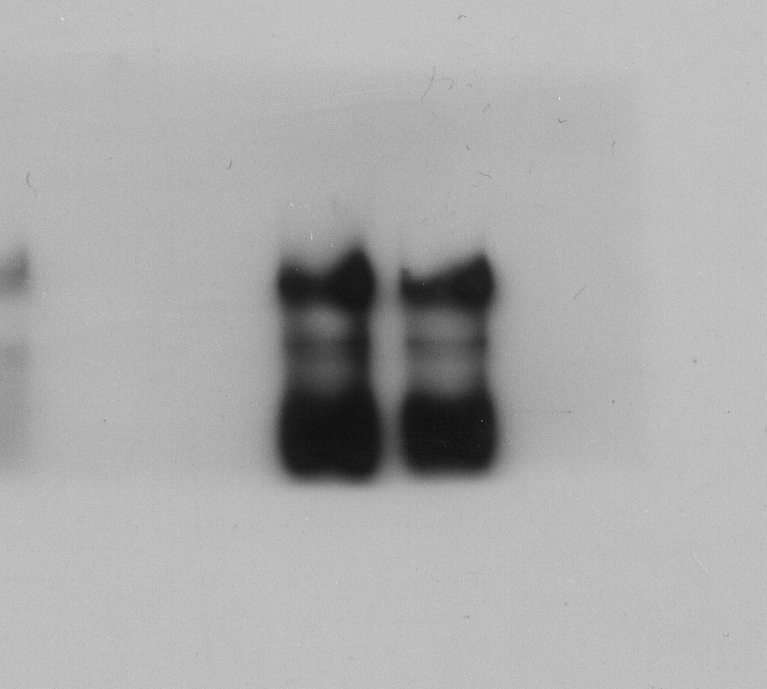

Supplement: Supplementary file 5 — Dataset 4 [file 41417_2024_729_MOESM5_ESM.zip › Figure 4/Figure 4e/u373/endo irs2.tif]

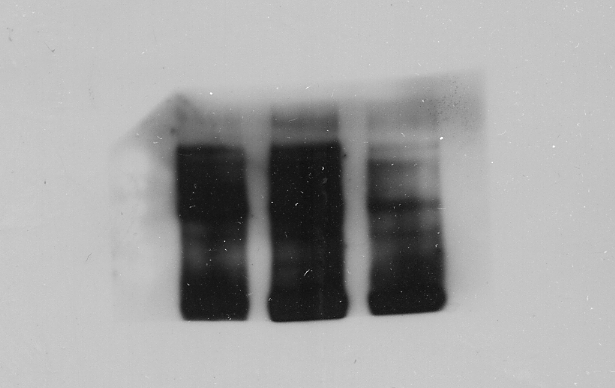

Supplement: Supplementary file 5 — Dataset 4 [file 41417_2024_729_MOESM5_ESM.zip › Figure 4/Figure 4e/u373/endo n8 for irs1.tif]

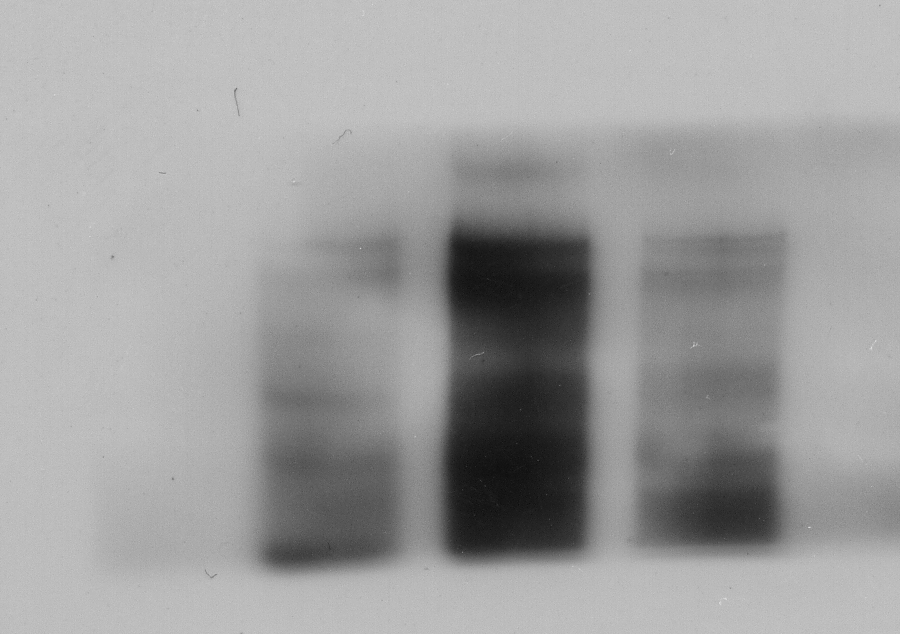

Supplement: Supplementary file 5 — Dataset 4 [file 41417_2024_729_MOESM5_ESM.zip › Figure 4/Figure 4e/u373/endo n8 for irs2.tif]

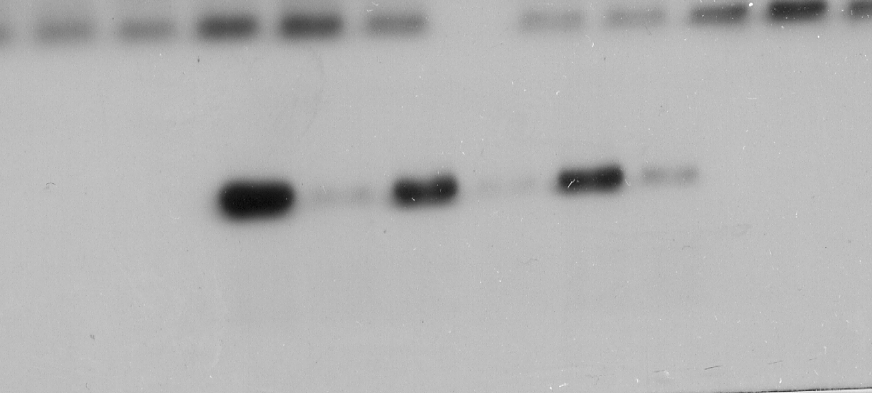

Supplement: Supplementary file 5 — Dataset 4 [file 41417_2024_729_MOESM5_ESM.zip › Figure 4/Figure 4f/rcc/cbl.tif]

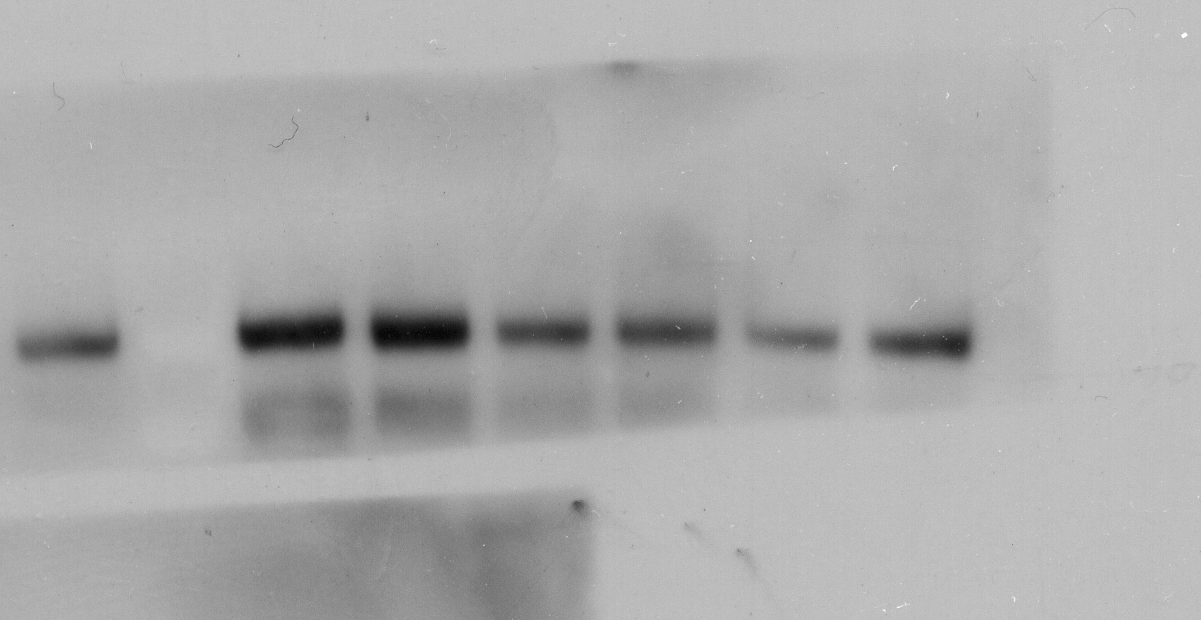

Supplement: Supplementary file 5 — Dataset 4 [file 41417_2024_729_MOESM5_ESM.zip › Figure 4/Figure 4f/rcc/irs1.tif]

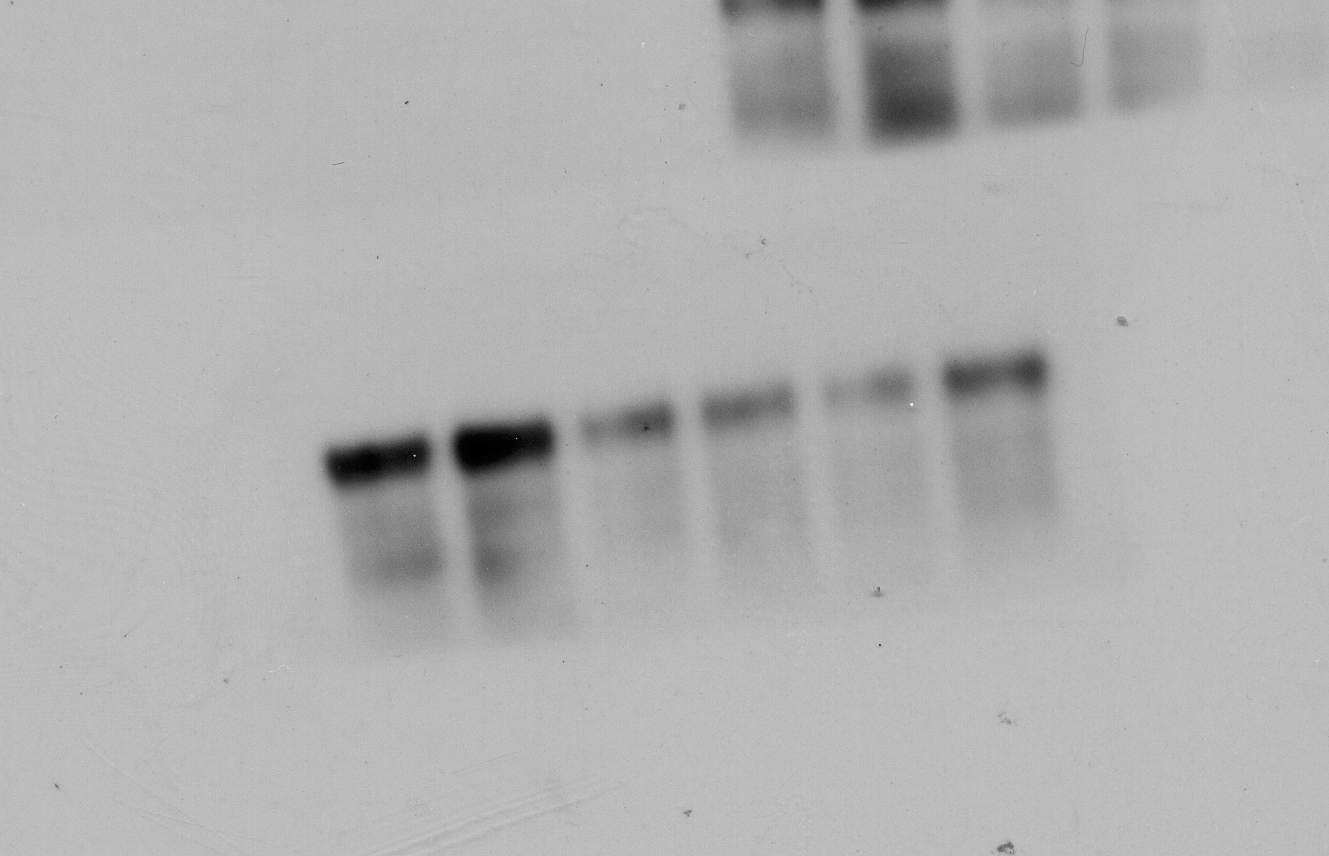

Supplement: Supplementary file 5 — Dataset 4 [file 41417_2024_729_MOESM5_ESM.zip › Figure 4/Figure 4f/rcc/irs2.tif]

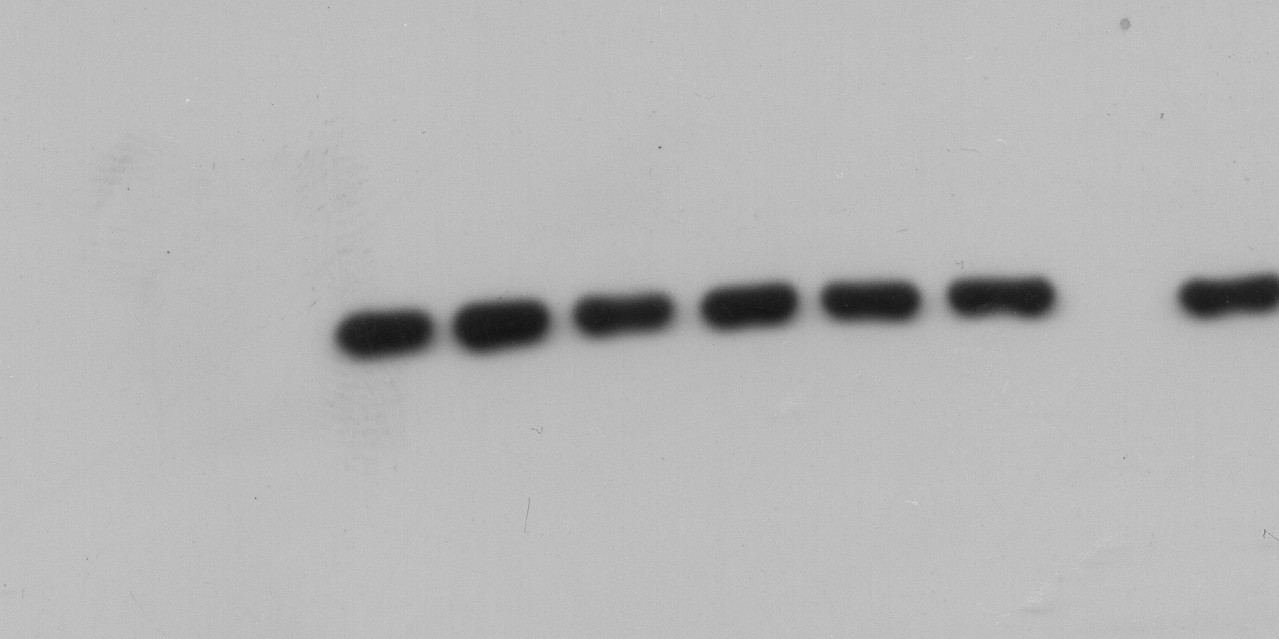

Supplement: Supplementary file 5 — Dataset 4 [file 41417_2024_729_MOESM5_ESM.zip › Figure 4/Figure 4f/rcc/tub.tif]

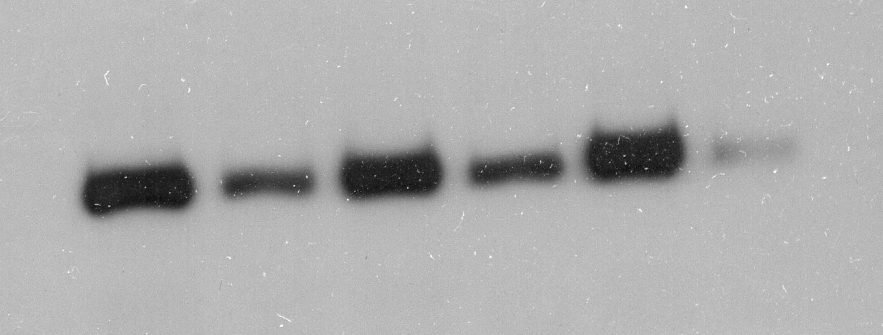

Supplement: Supplementary file 5 — Dataset 4 [file 41417_2024_729_MOESM5_ESM.zip › Figure 4/Figure 4f/skov/cbl.tif]

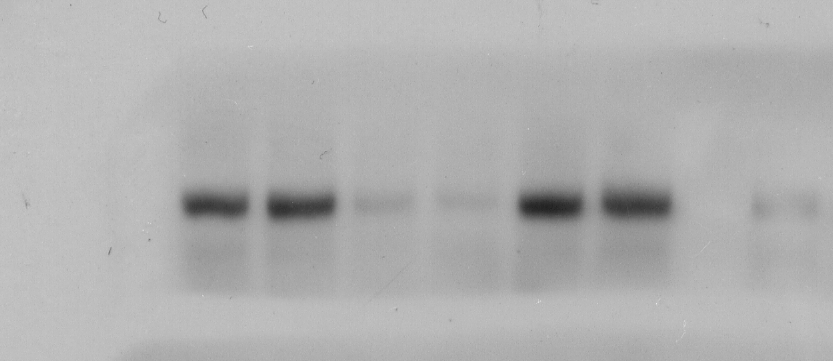

Supplement: Supplementary file 5 — Dataset 4 [file 41417_2024_729_MOESM5_ESM.zip › Figure 4/Figure 4f/skov/irs1.tif]

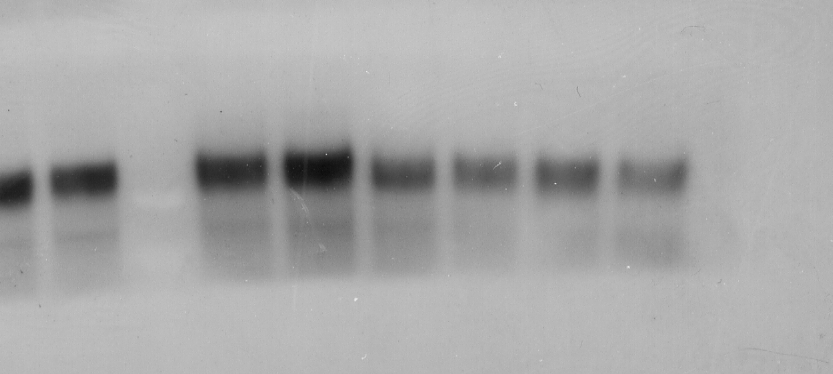

Supplement: Supplementary file 5 — Dataset 4 [file 41417_2024_729_MOESM5_ESM.zip › Figure 4/Figure 4f/skov/irs2.tif]

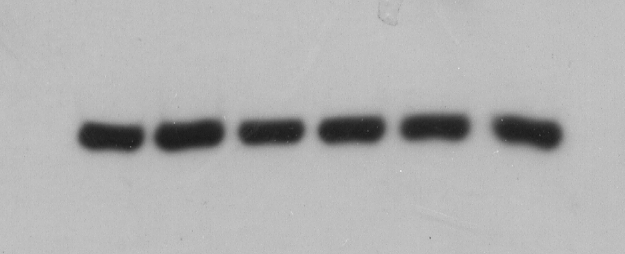

Supplement: Supplementary file 5 — Dataset 4 [file 41417_2024_729_MOESM5_ESM.zip › Figure 4/Figure 4f/skov/tub.tif]

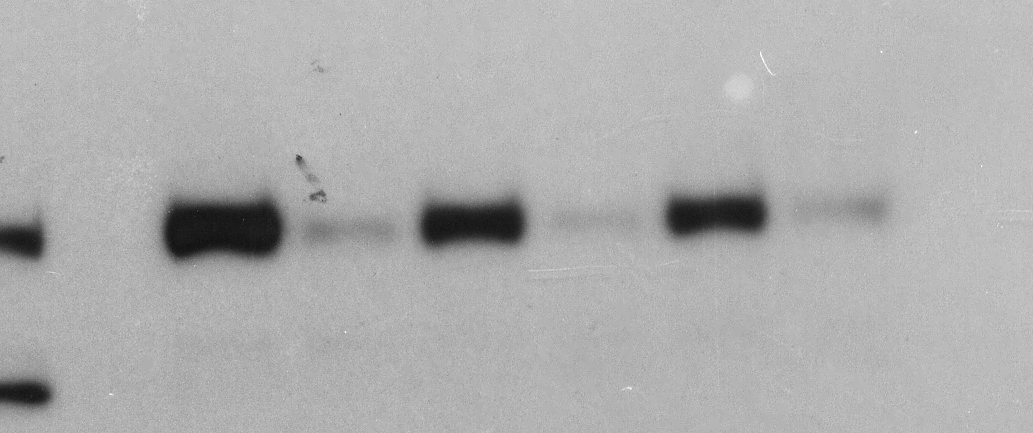

Supplement: Supplementary file 5 — Dataset 4 [file 41417_2024_729_MOESM5_ESM.zip › Figure 4/Figure 4f/u373/cbl.tif]

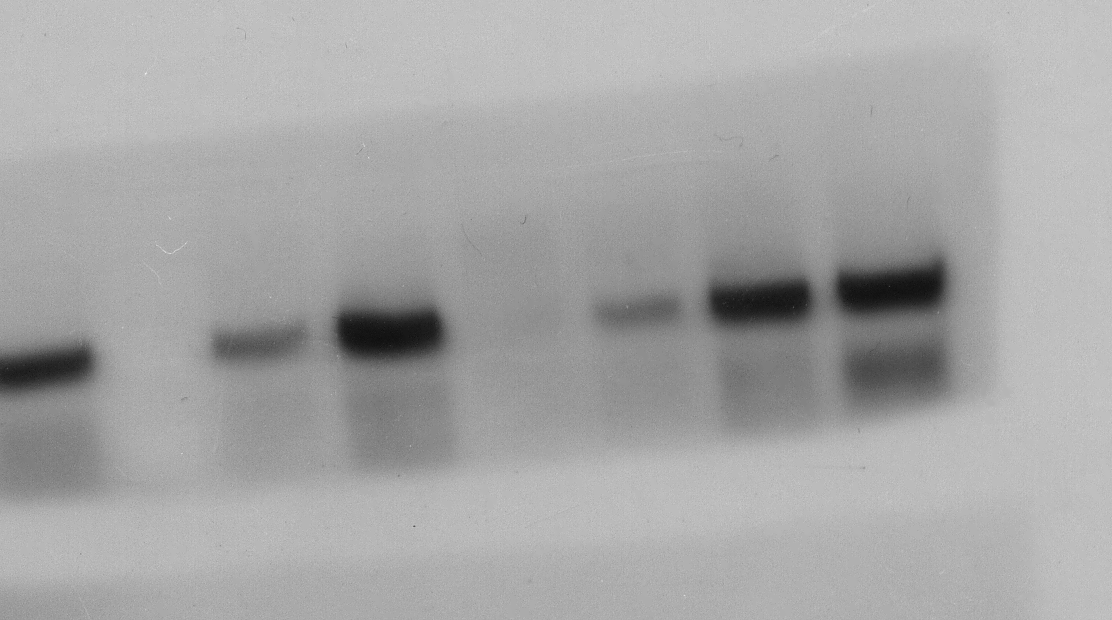

Supplement: Supplementary file 5 — Dataset 4 [file 41417_2024_729_MOESM5_ESM.zip › Figure 4/Figure 4f/u373/irs1.tif]

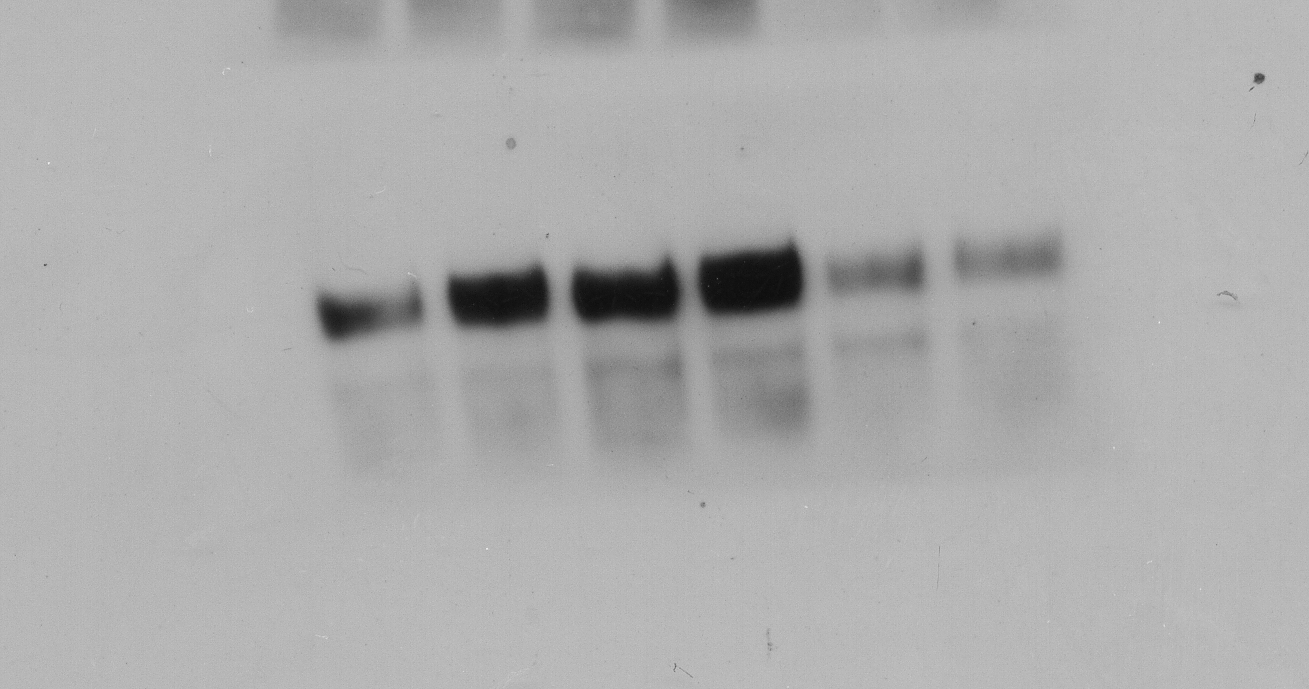

Supplement: Supplementary file 5 — Dataset 4 [file 41417_2024_729_MOESM5_ESM.zip › Figure 4/Figure 4f/u373/irs2.tif]

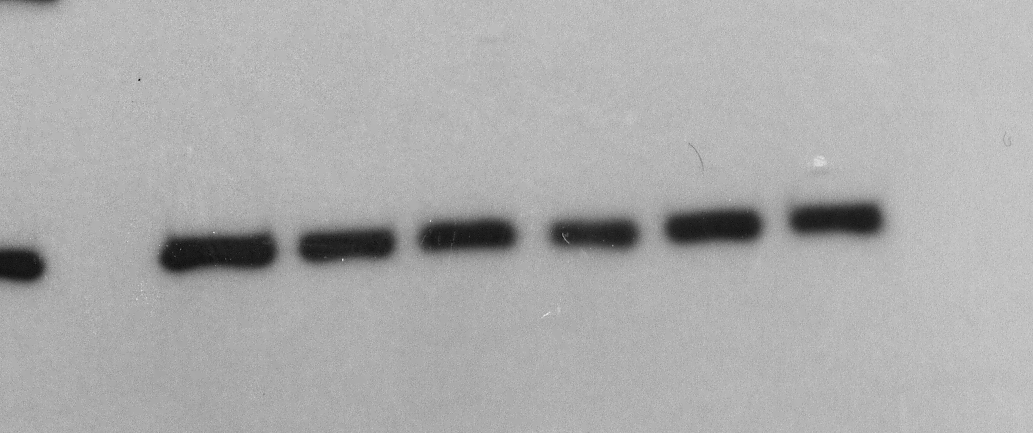

Supplement: Supplementary file 5 — Dataset 4 [file 41417_2024_729_MOESM5_ESM.zip › Figure 4/Figure 4f/u373/tub.tif]

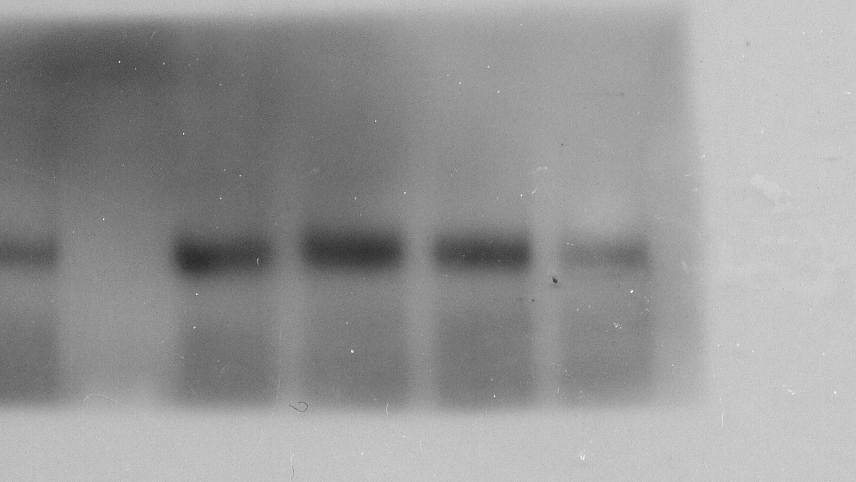

Supplement: Supplementary file 6 — Supplementary Dataset [file 41417_2024_729_MOESM6_ESM.zip › Supplementary Dataset/Supplementary Figures/Figure S1/RCC4/rcc4 ins irs1.tif]

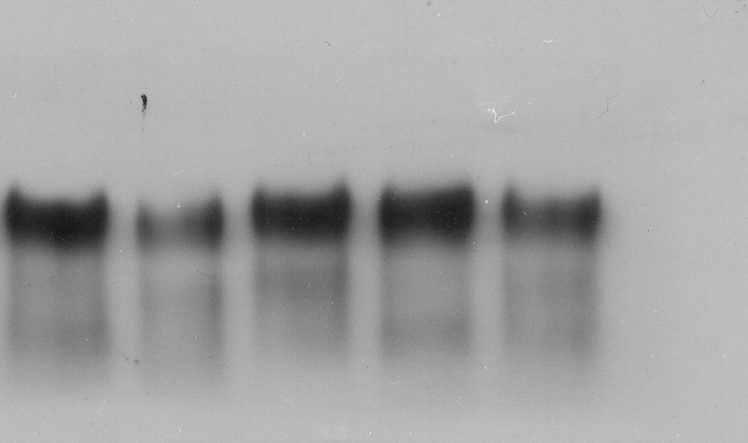

Supplement: Supplementary file 6 — Supplementary Dataset [file 41417_2024_729_MOESM6_ESM.zip › Supplementary Dataset/Supplementary Figures/Figure S1/RCC4/rcc4 ins irs2.tif]

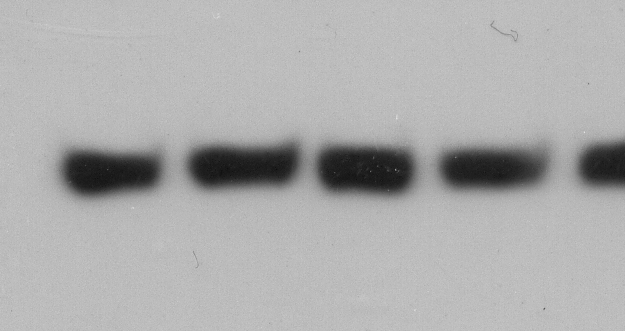

Supplement: Supplementary file 6 — Supplementary Dataset [file 41417_2024_729_MOESM6_ESM.zip › Supplementary Dataset/Supplementary Figures/Figure S1/RCC4/rcc4 ins tub.tif]

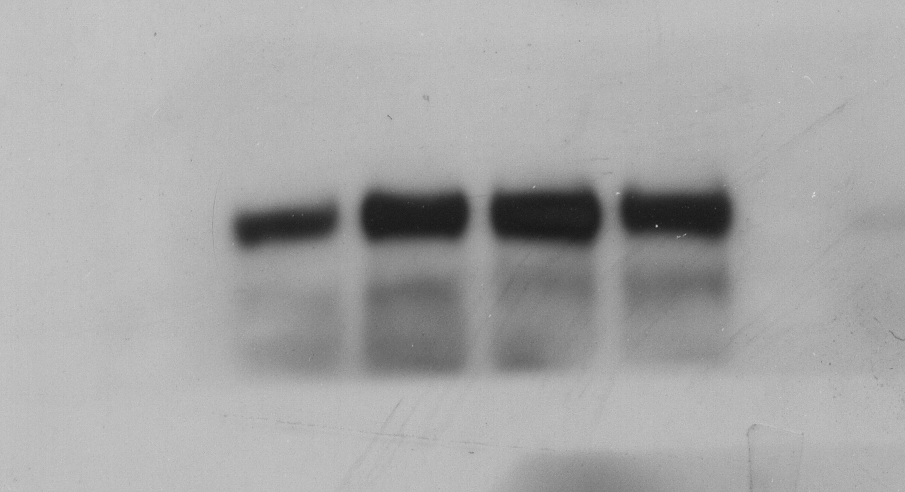

Supplement: Supplementary file 6 — Supplementary Dataset [file 41417_2024_729_MOESM6_ESM.zip › Supplementary Dataset/Supplementary Figures/Figure S1/RCC4/rcc4 mln irs1.tif]

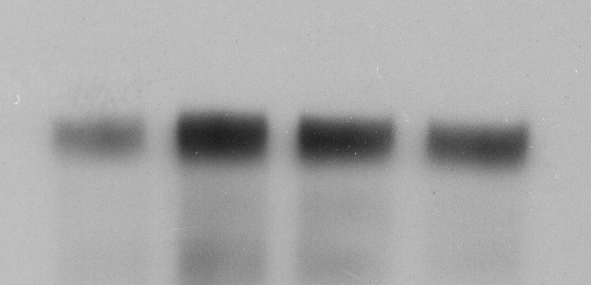

Supplement: Supplementary file 6 — Supplementary Dataset [file 41417_2024_729_MOESM6_ESM.zip › Supplementary Dataset/Supplementary Figures/Figure S1/RCC4/rcc4 mln irs2.tif]

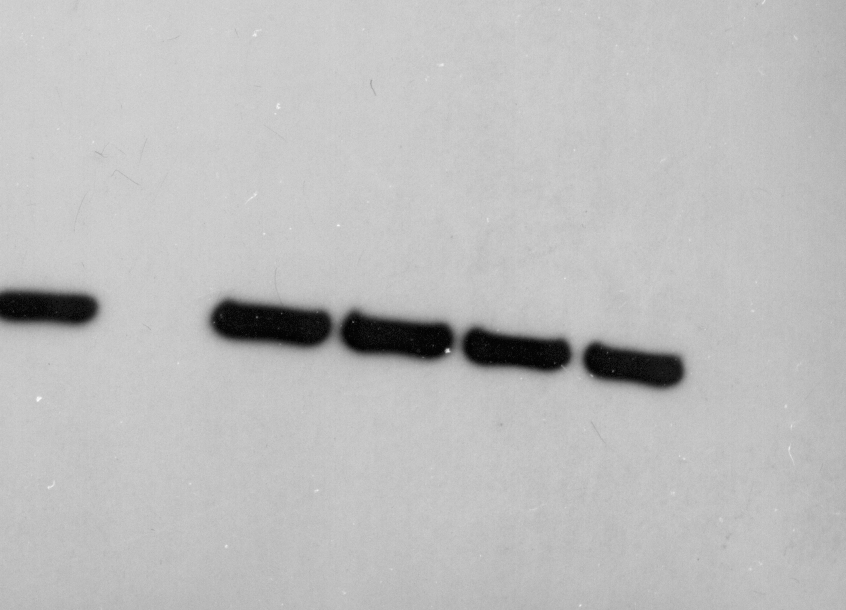

Supplement: Supplementary file 6 — Supplementary Dataset [file 41417_2024_729_MOESM6_ESM.zip › Supplementary Dataset/Supplementary Figures/Figure S1/RCC4/rcc4 mln tub.tif]

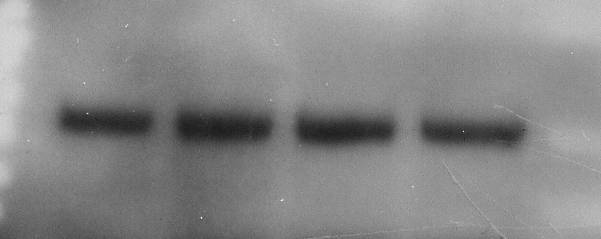

Supplement: Supplementary file 6 — Supplementary Dataset [file 41417_2024_729_MOESM6_ESM.zip › Supplementary Dataset/Supplementary Figures/Figure S1/SKOV3/skov3 ins IRS1.tif]

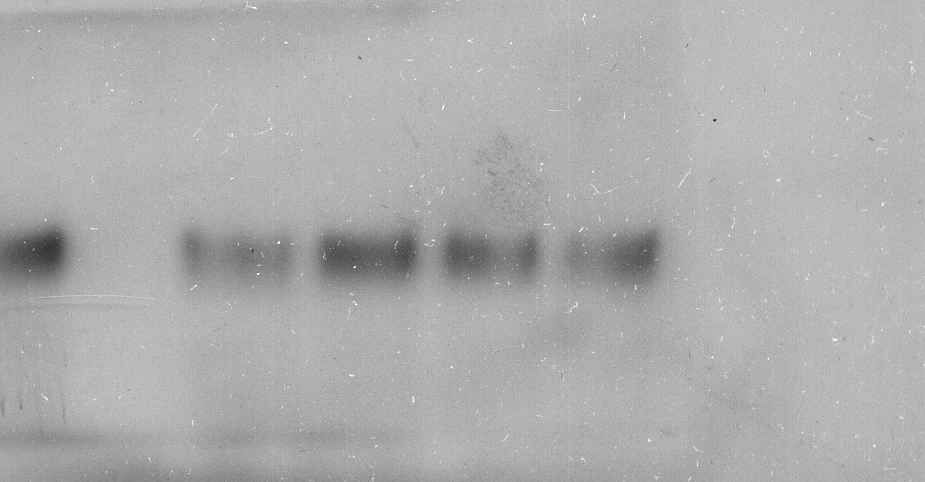

Supplement: Supplementary file 6 — Supplementary Dataset [file 41417_2024_729_MOESM6_ESM.zip › Supplementary Dataset/Supplementary Figures/Figure S1/SKOV3/skov3 ins IRS2.tif]

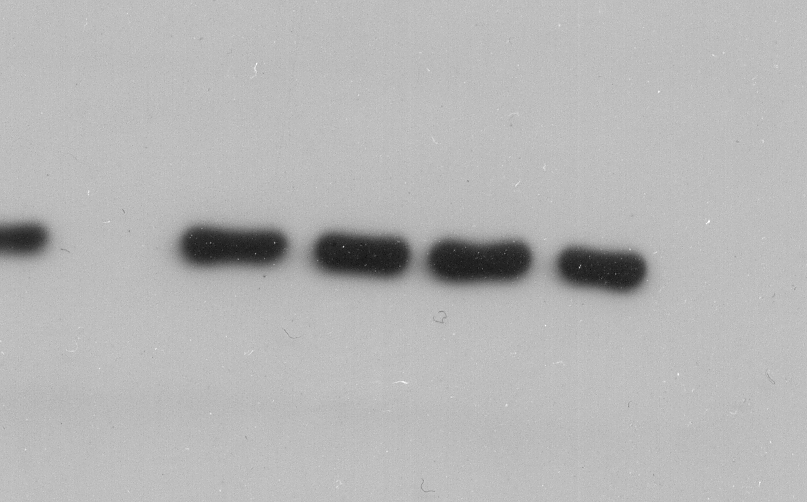

Supplement: Supplementary file 6 — Supplementary Dataset [file 41417_2024_729_MOESM6_ESM.zip › Supplementary Dataset/Supplementary Figures/Figure S1/SKOV3/skov3 ins tub.tif]

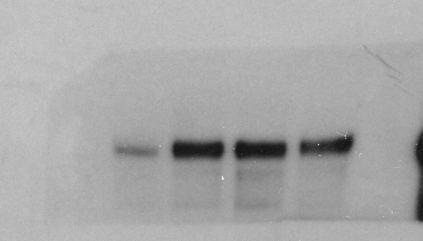

Supplement: Supplementary file 6 — Supplementary Dataset [file 41417_2024_729_MOESM6_ESM.zip › Supplementary Dataset/Supplementary Figures/Figure S1/SKOV3/skov3 mln IRS1.tif]

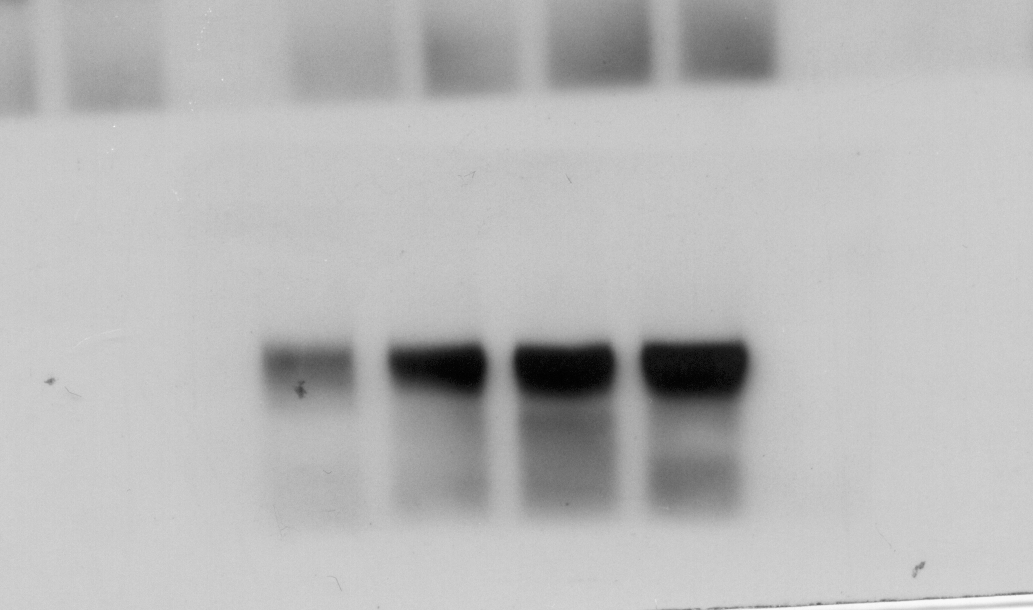

Supplement: Supplementary file 6 — Supplementary Dataset [file 41417_2024_729_MOESM6_ESM.zip › Supplementary Dataset/Supplementary Figures/Figure S1/SKOV3/SKOV3 mln IRS2.tif]
