# Supplementary figures and images for: Neddylation of insulin receptor substrate acts as a bona fide regulator of insulin signaling and its implications for cancer cell migration (part 3 of 3)
Source: Cancer Gene Ther. 2024 Jan 25;31(4):599–611. doi: 10.1038/s41417-024-00729-z (PMC11016467; doi:10.1038/s41417-024-00729-z)

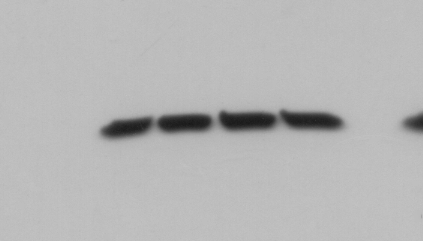

Supplement: Supplementary file 6 — Supplementary Dataset [file 41417_2024_729_MOESM6_ESM.zip › Supplementary Dataset/Supplementary Figures/Figure S1/SKOV3/SKOV3 mln tub.tif]

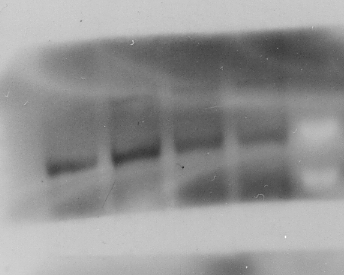

Supplement: Supplementary file 6 — Supplementary Dataset [file 41417_2024_729_MOESM6_ESM.zip › Supplementary Dataset/Supplementary Figures/Figure S1/U373/U373 ins IRS1.tif]

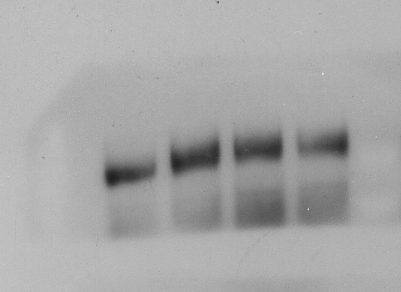

Supplement: Supplementary file 6 — Supplementary Dataset [file 41417_2024_729_MOESM6_ESM.zip › Supplementary Dataset/Supplementary Figures/Figure S1/U373/U373 ins IRS2.tif]

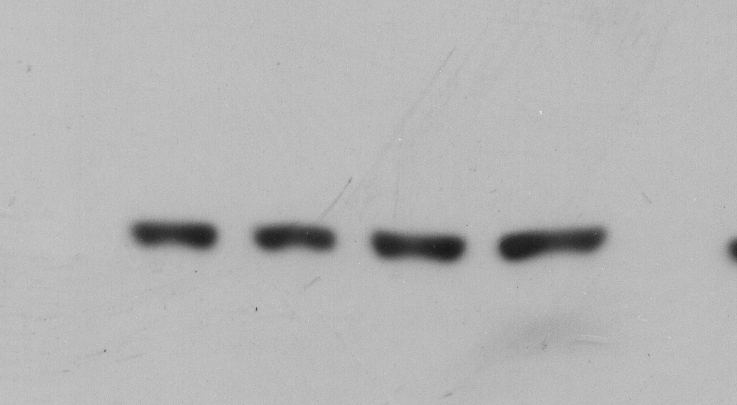

Supplement: Supplementary file 6 — Supplementary Dataset [file 41417_2024_729_MOESM6_ESM.zip › Supplementary Dataset/Supplementary Figures/Figure S1/U373/U373 ins Tubulin.tif]

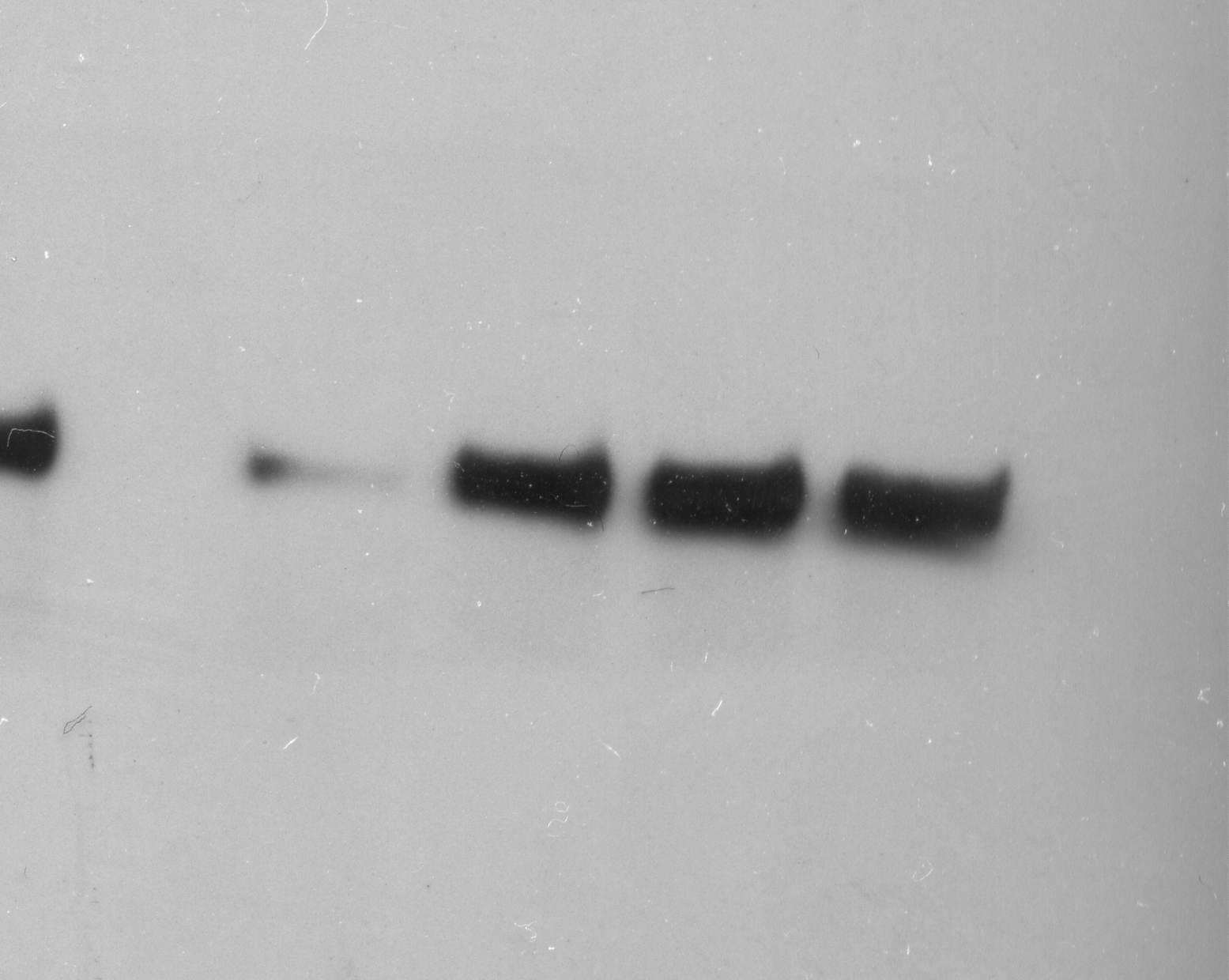

Supplement: Supplementary file 6 — Supplementary Dataset [file 41417_2024_729_MOESM6_ESM.zip › Supplementary Dataset/Supplementary Figures/Figure S1/U373/u373 mln IRS1.tif]

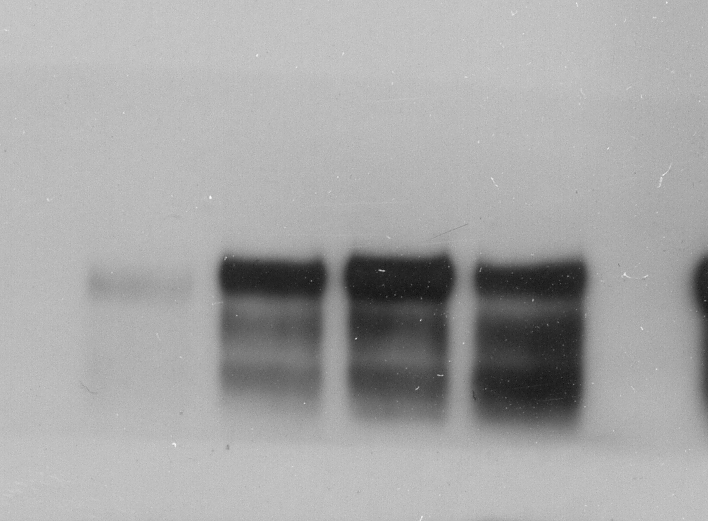

Supplement: Supplementary file 6 — Supplementary Dataset [file 41417_2024_729_MOESM6_ESM.zip › Supplementary Dataset/Supplementary Figures/Figure S1/U373/U373 mln irs2.tif]

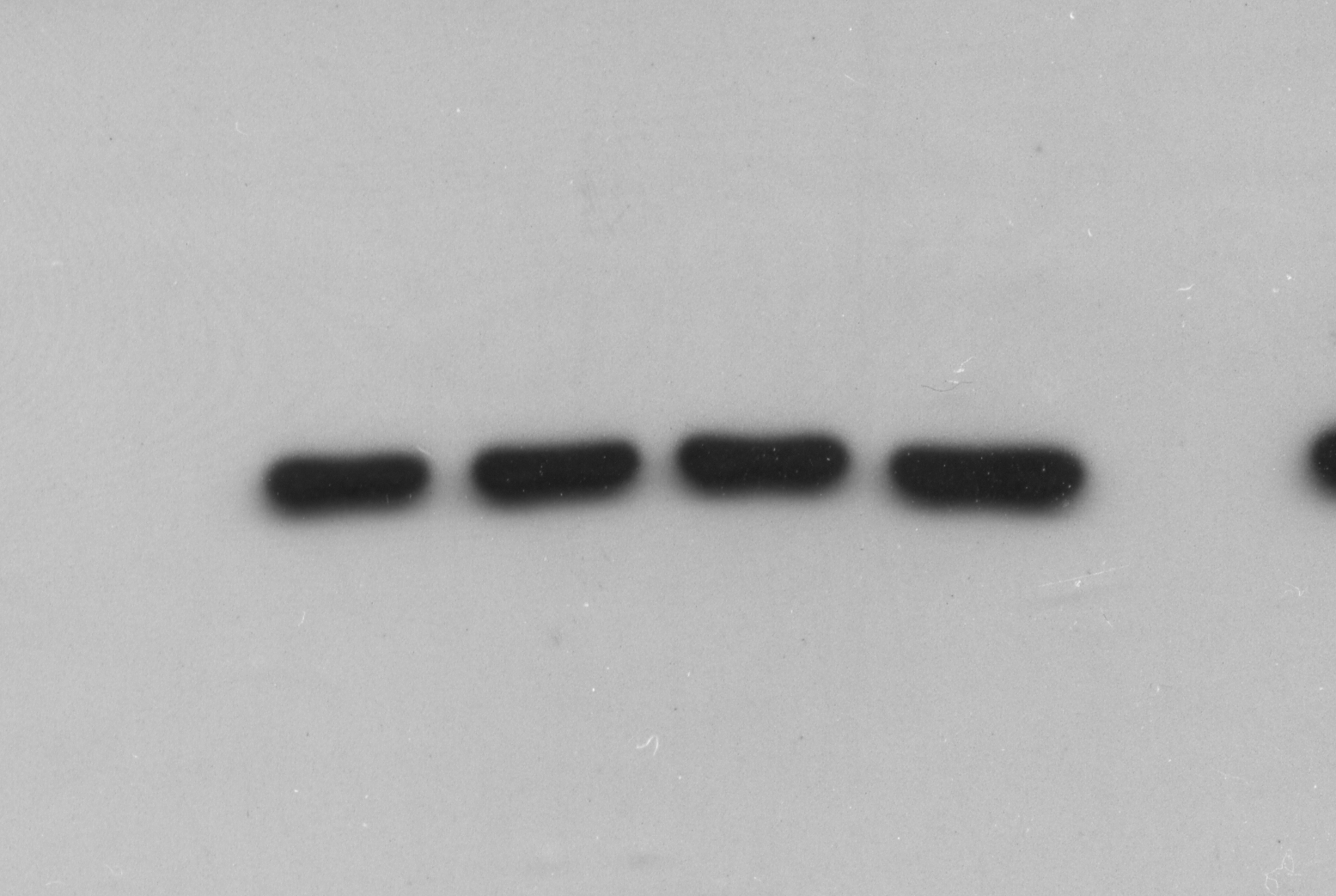

Supplement: Supplementary file 6 — Supplementary Dataset [file 41417_2024_729_MOESM6_ESM.zip › Supplementary Dataset/Supplementary Figures/Figure S1/U373/U373 mln tub.tif]

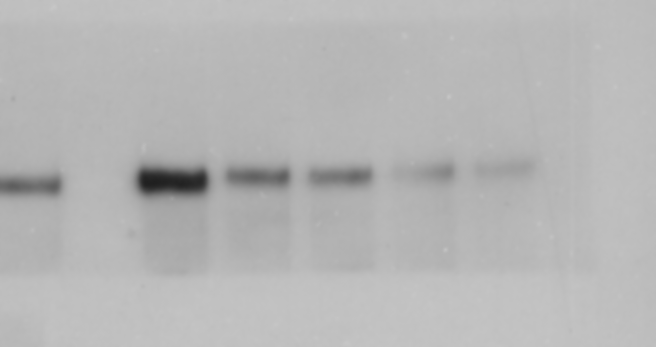

Supplement: Supplementary file 6 — Supplementary Dataset [file 41417_2024_729_MOESM6_ESM.zip › Supplementary Dataset/Supplementary Figures/Figure S3/RCC4/rcc4 irs1.tif]

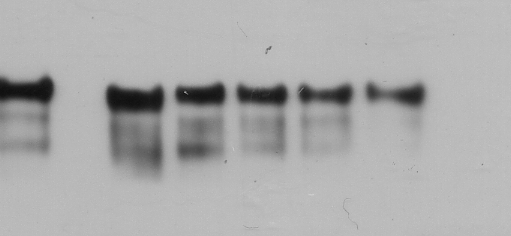

Supplement: Supplementary file 6 — Supplementary Dataset [file 41417_2024_729_MOESM6_ESM.zip › Supplementary Dataset/Supplementary Figures/Figure S3/RCC4/rcc4 irs2.tif]

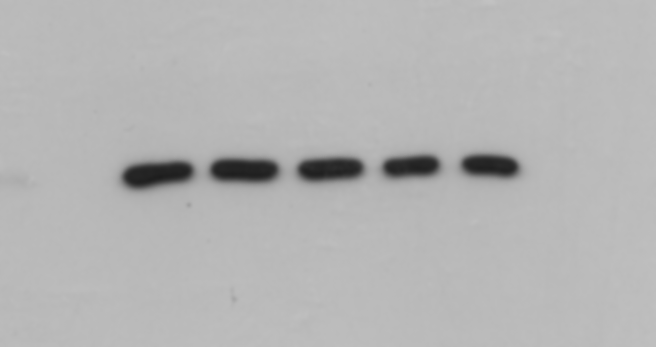

Supplement: Supplementary file 6 — Supplementary Dataset [file 41417_2024_729_MOESM6_ESM.zip › Supplementary Dataset/Supplementary Figures/Figure S3/RCC4/rcc4 tub.tif]

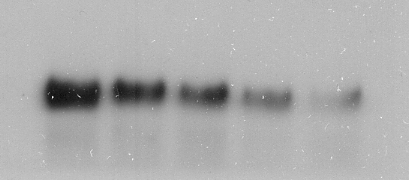

Supplement: Supplementary file 6 — Supplementary Dataset [file 41417_2024_729_MOESM6_ESM.zip › Supplementary Dataset/Supplementary Figures/Figure S3/SKOV3/skov3 IRS1.tif]

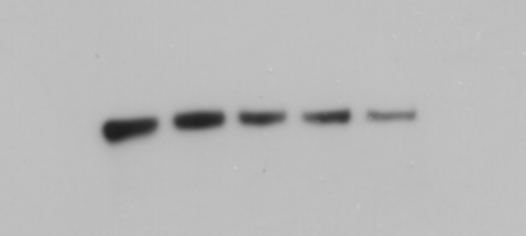

Supplement: Supplementary file 6 — Supplementary Dataset [file 41417_2024_729_MOESM6_ESM.zip › Supplementary Dataset/Supplementary Figures/Figure S3/SKOV3/skov3 IRS2.tif]

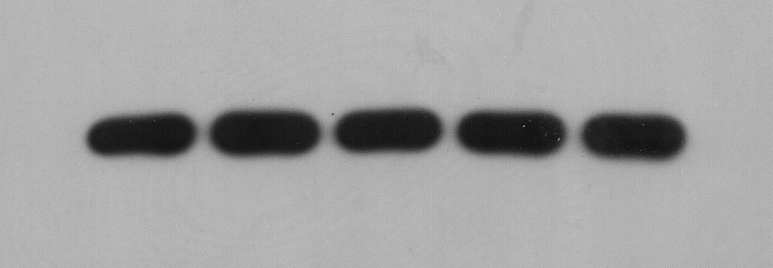

Supplement: Supplementary file 6 — Supplementary Dataset [file 41417_2024_729_MOESM6_ESM.zip › Supplementary Dataset/Supplementary Figures/Figure S3/SKOV3/SKOV3 tub.tif]

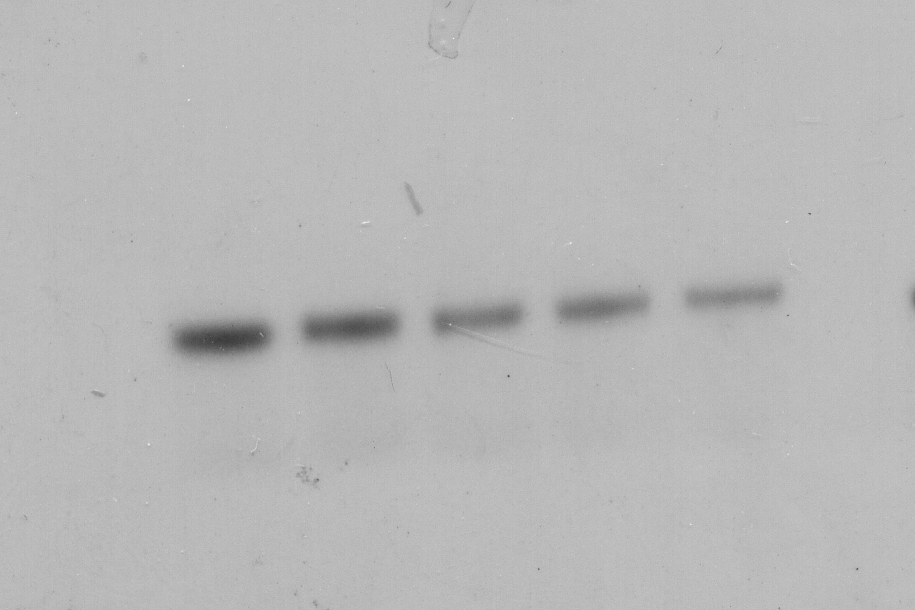

Supplement: Supplementary file 6 — Supplementary Dataset [file 41417_2024_729_MOESM6_ESM.zip › Supplementary Dataset/Supplementary Figures/Figure S3/U373/U373 IRS1.tif]

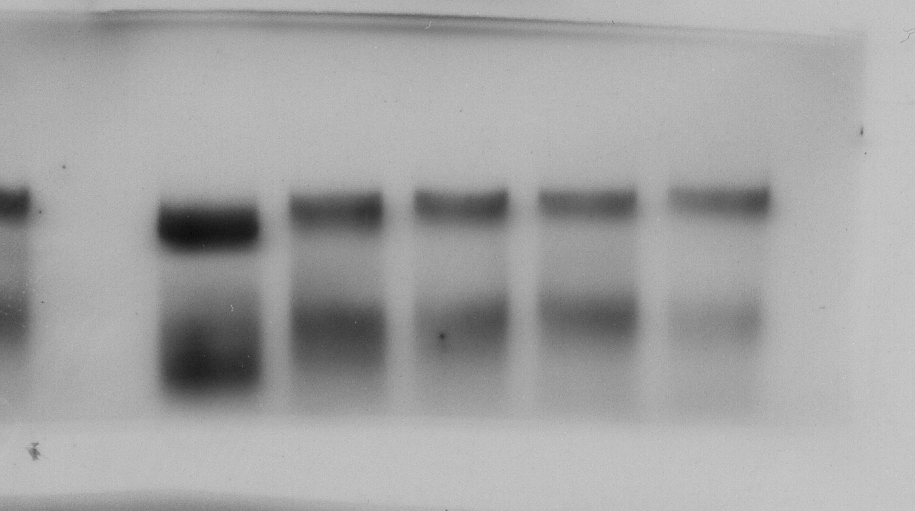

Supplement: Supplementary file 6 — Supplementary Dataset [file 41417_2024_729_MOESM6_ESM.zip › Supplementary Dataset/Supplementary Figures/Figure S3/U373/U373 IRS2.tif]

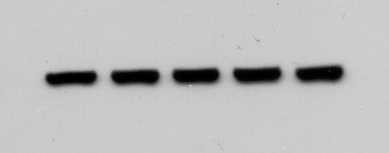

Supplement: Supplementary file 6 — Supplementary Dataset [file 41417_2024_729_MOESM6_ESM.zip › Supplementary Dataset/Supplementary Figures/Figure S3/U373/U373 TUB.tif]
